# Characterization of the *Fatty Acid Desaturase* Genes in Cucumber: Structure, Phylogeny, and Expression Patterns

**DOI:** 10.1371/journal.pone.0149917

**Published:** 2016-03-03

**Authors:** Chun-Juan Dong, Ning Cao, Zhi-Gang Zhang, Qing-Mao Shang

**Affiliations:** Institute of Vegetables and Flowers, Chinese Academy of Agricultural Sciences, Key Laboratory of Horticultural Crop Biology and Germplasm Innovation, Ministry of Agriculture, Beijing, People’s Republic of China; Hainan University, CHINA

## Abstract

Fatty acid desaturases (FADs) introduce double bonds into the hydrocarbon chains of fatty acids to produce unsaturated fatty acids, and therefore play a critical role in plant development and acclimation to environmental stresses. In this study, 23 full-length *FAD* genes in cucumber (*Cucumis sativus* L.) were identified through database searches, including three *CsFAB2* genes, two *CsFAD2* genes, fourteen *CsFAD5* genes, and one gene each for *CsFAD3*, *CsFAD4*, *CsFAD6* and *CsFAD7*. These cucumber *FAD* genes were distributed on all seven chromosomes and two additional scaffolds. Based on a phylogenetic analysis, the cucumber FAD proteins were clustered into five subfamilies with their counterparts from other plants. Gene structures and protein sequences were considerably conserved in each subfamily. All three CsFAB2 proteins shared conserved structure with the known plant soluble FAD proteins. The other cucumber FADs belonged to the membrane-bound FADs and contained three highly conserved histidine boxes. Additionally, the putative endoplasmic reticulum retention signal was found at the C-termini of the CsFAD2 and CsFAD3 proteins, while the N-termini of CsFAD4, CsFAD5, CsFAD6, CsFAD7 and three CsFAB2s contained a predicted chloroplast signal peptide, which was consistent with their associated metabolic pathways. Furthermore, a gene expression analysis showed that *CsFAD2* and *CsFAD3* were universally expressed in all tested tissues, whereas the other cucumber *FAD* genes were preferentially expressed in the cotyledons or leaves. The tissue-specific expression patterns of cucumber *FAD* genes were correlated well with the differences in the fatty acid compositions ofroots and leaves. Finally, the cucumber *FAD* genes showed a cold-induced and heat-repressed expression pattern, although with distinct regulatory time courses among the different *CsFAD* members, which indicates the potential roles of the FADs in temperature stress resistance in cucumber.

## Introduction

Fatty acids are the main components of plant membrane lipids and seed storage lipids [1]. In plant cells, fatty acids are generated*de novo* in the stroma of plastids [2]. Once biosynthesized, these fatty acids are then incorporated into the glycerolipid synthetic pathways in plastids or endoplasmic reticulum (ER) for assembly into galactoglycerolipids, sulfolipids, and phospholipids [2].

High level of fatty acid desaturation is a common feature for the plant cell membranes [3]. Unsaturated fatty acids usually contain one or more double bonds in their hydrocarbon chains [4]. The content of unsaturated fatty acids is very important in terms of nutritional characteristics of edible oils [4]. Also, the number and position of the double bonds in a fatty acidmarkedly influence its physical and physiological properties [5, 6]. The composition of the unsaturated fatty acids is critically important for the membranes function, and thereby for the proper growth and development ofplants [5]. Furtermore, the level of unsaturation is one of the major determining factors in the tolerance of a given plant to various environmental stresses, especially temperature stresses [7]. Unsaturated fatty acids have a lower melting temperature than saturated fatty acids, and their increased accumulation is thought to help cold acclimation by maintaining the appropriate fluidity and integrity of membranes; however, unsaturated fatty acid accumulation can also aggravate heat damage [8].

Disaturation of the fatty acids is performed by a series of enzymes called fatty acid desaturases (FADs) [3]. Given the functional importance of fatty acid desaturation in plant development and stress responses, *FAD* genes have been identified and characterized in many plant species. In Arabidopsis, the properties of its FADs have been illucidated by characterization of several classes of mutants, each on deficient in a specific desaturation step [3]. These enzymes are encoded by nuclear genes but affect the desaturation in different subcellular localization. FAD2 and FAD3 are located inER and primarily affect desaturation of the extrachloroplast lipids, whereas the others (FAB2, FAD4, FAD5, FAD6, FAD7, and FAD8) are located in the plastids and affect plastid lipid desaturation [3].These FADs are highly substrate specific. FAB2 belongs to the soluble FAD and is responsible for the desaturation of stearic acid (18:0) to oleic acid (18:1) in an acyl-carrier protein (ACP)-bound form [9, 10], and therefore FAB2 is also named stearoyl-ACP desaturase (SAD). The remaining FADs are membrane-bound. FAD2 and FAD6 are ω-6 desaturases that synthesize the dienoic fatty acid linoleic acid (18:2) from oleic acid (18:1) in the ER and plastids, respectively. FAD3, FAD7 and FAD8 are ω-3 desaturases that insert the double bond in linoleic acid (18:2) to synthesize linolenic acid (18:3) in the ER (FAD3) and plastids (FAD7 and FAD8). The *FAD8* gene encodes a cold-inducible isoform of FAD7 [11]. FAD4 and FAD5 act on palmitic acid (16:0) specifically from phosphatidylglycerol and monogalactosyldiacylglycerol, respectively, to produce palmitoleic acid (16:1) in plastids [5]. In recent years, *FAD* genes have also been identified in many oilseed crops, including soybean [12, 13], cotton [14, 15], cacao [10] and olive [4, 16]. For example, 29 desaturase genes were identified in soybean [12]. In cotton (*Gossypium raimondii*), its membrane-bound FADs wereencoded by 19 genes[15]. In olive, two *FAD3* (*FAD3A* and *FAD3B*) and two *FAD7* (*FAD7-1* and *FAD7-2*) genes were isolated. Of them, *FAD3A* was mainly responsible for the 18:3 present in seeds, while two *FAD7* genes contributed mostly to the 18:3 in the mesocarp of fruits [4, 16].

FADs are also crucial for supplying sustenance during various environmental stressesin plants. In Arabidopsis, the expression of *FAD8*is strongly induced by cold temperatures [11]. Moreover, *FAD2* and *FAD6*are activated in seedlings under salinity and osmotic stresses [17–19]. An *FAD2*-deficient mutant accumulated more Na^+^ in the cytoplasm of its root cells and was highly sensitive to salt stress during seed germination and early seedling growth [18]. Moreover, transgenic tobacco plants overexpressing the Arabidopsis *FAD7*gene showed enhanced cold stress, whereas plants with its own*FAD7* gene silencedcontained lower levels of trienoic fatty acids and were more tolerant to high temperatures than the wild-type plants [20–22]. Tobacco plants overexpressing either Arabidopsis *FAD3* or *FAD8*gene also exhibited increased tolerance to drought and osmotic stress [23]. In soybean (*Glycine max*), the expression of *GmFAD3* and *GmFAD7* is tightly regulated under cold temperatures [13]. In tomato (*Lycopersicon esculentum*), the expression of *LeFAD7* is inhibited by heat stress, and silencing the *LeFAD7* gene alleviated high-temperature stress [24]. For another ω-3 *FAD* gene (*LeFAD3*) in tomato, its expression is induced by salinity stress, and *LeFAD3*-overexpression enhanced the tolerance of tomato seedlings to salinity stress [25].

Cucumber (*Cucumis sativus* L.) is an economically important vegetable that is grown worldwide. Cucumber is planted at various times throughout the year, and the beginning and end of the growing season often include sub-optimal growth temperatures. Late in the growing season, heat stress always occurs and causes significant reductions in yield. During the early phase of the growing season, the exposure of cucumber seedlings to sudden episodes of cold can cause serious damage to the plants because the tropical origin of cucumber renders it cold sensitive [26]. Undoubtedly, characterization of *FAD*s would provide some candidate genes to develop new cucumber varieties with enhanced stress tolerance. Taking advantage of the available cucumber genome database [27], a systematic characterization of cucumber *FAD* genes was carried out.In this study, 23 cucumber *FAD* genes were identified through homology searches. The detailed analysis including the phylogenies, chromosomal localizations, structures and conserved motifs were further performed.In addition, the *in silico* and experimental expression patterns of the cucumber *FAD* genes were analyzed and provided valuable information for further exploration into the functions of this significant gene family in cucumber.

## Materials and Methods

### Database searching and sequence analysis

The FAD sequence data were collected by homology screening against the cucumber genome database (ICuGI, http://www.icugi.org/cgi-bin/ICuGI/index.cgi) [27]. Homology searches were performed using tBlastn with the default parameters. The known FAD sequences from Arabidopsis (as listed in S1 Table) were used as queries.

To illustrate the exon-intron gene structure for each cucumber *FAD* gene, the Gene Structure Display Server (GSDS, http://gsds1.cbi.pku.edu.cn/) was employed to compare the predicted coding sequence (CDS) with its corresponding genomic sequence derived from the ICuGI database. Next, introns were confirmed by amplifying the full-length cDNA sequences with primers specific for the 5’- and 3’-untranslated regions (UTRs) of the *CsFAD* genes. The molecular weights (MWs) and isoelectric points (p*I*s) of the deduced proteins were predicted using ExPASy (http://www.cn.expasy.org/tools). Transient signal peptides were predicted using TargetP 1.1 (http://www.cbs.dtu.dk/services/TargetP/) [28] and the ChloroP 1.1 server (http://www.cbs.dtu.dk/services/ChloroP/) [29, 30].

### Multiple sequence alignments and phylogenetic analyses

The plant FAD sequences were downloaded from the ICuGI, TAIR (http://www.arabidopsis.org) and GeneBank databases. The accession numbers are listed in S1 Table. Multiple sequence alignments of the full-length FAD amino acid sequences were performed using ClustalX (version 2.0) with the default parameters [31]. A Neighbor-Joining (NJ) phylogenetic tree was constructed using MEGA 5.2 [32]. Bootstrap tests were performed with 1,000 replicates for statistical reliability.

### *K*_a_/*K*_s_ analysis and calculation of the duplication date

The DnaSP (version 5.10.01) software was used to estimate the synonymous (*K*_s_) and non-synonymous (*K*_a_) substitution rates by aligning the original cDNA sequences of duplicated *CsFAD* genes [33]. Time (million years ago, mya) of duplication and divergence of *CsFAD* genes was calculated according to the equation *T* = *K*_s_/2λ (λ = 6.5×10^−9^) [34].

### Plant growth and treatment

Cucumber (*C*. *sativus* L. cv. ‘Zhongnong No. 203’), a cultivar commonly used in north China,were used in this study. This cultivar has the similar genetic background with the ‘Chinese long’ inbred line whose genome has been sequenced[27, 35]. The cucumber seeds were surface sterilized in 5% NaClO and sown in vermiculite. After 7 days, the seedlings with completely outspread cotyledons were transferred to a hydroponic culture system containing half-strength Hoagland’s nutrient solution (pH 6.5) in a chamber. The growth conditions were set as follows: a 12-h photoperiod, 28°C/18°C day/night temperatures, 75%-85% relative humidity, and a light intensity of 300 μmol m^-2^ s^-1^. The 21-d-old seedlings whose first true leaves had been fully expanded were used for the subsequent expression profiling and fatty acid analysis.

For the tissue-specific expression assay of *FAD* genes, the leaves, cotyledons, hypocotyls and roots of the cucumber seedlings were sampled. To examine the stress- and hormone-inducible expression patterns of *FAD*s, the cucumber seedlings were treated at 8°C for cold stress, with a light intensity of 100 μmol m^-2^ s^-1^. Heat stress was maintained by exposing the plants to temperatures of 38°C/28°C (day/night). The seedlings were also subjected to a nutrient solution supplemented with ABA (100 μM) or H_2_O_2_ (1 mM). After 0, 6, 12, 24 and 48 h of treatment, the leaves were harvested. The samples were immediately frozen in liquid nitrogen and stored at -80°C until use. Three biological replicates were conducted per sample, with 15 seedlings per replicate.

### Expression analysis of cucumber *FAD* genes

*In silico* expression analysis was accomplished by searching the PlantGDB databases including EST, cDNA and PUTs (PlantGDB unique transcripts). In addition, the cucumber EST collection (version 3.0) (http://www.icugi.org/cgi-bin/ICuGI/EST/home.cgi?organism=cucumber) from ICuGI database was also searched.

Moreover, the real-time quantitative RT-PCRexpression profile was also performed. Total RNA was extracted using the EASYPure Plant RNA kit (Transgen, Beijing, China) according to the manufacturer’s protocol. The concentration of RNA was measured using a Nanodrop 2000c spectrophotometer (Thermo Scientific, USA). Residual DNA was digested with RNase-free DNase I (Sigma-Aldrich, USA) by incubation at 37°C for 30 min.2 μg of total RNA was used as the template for the first-strand cDNA synthesis using a reverse-transcription system with oligo-dT_15_ as primer (Promega, USA). Real-time PCR (qPCR) was then performed in a total reaction volume of 20 μl containing 5 μl diluted cDNA (1:20), 5 μl Power SYBR-Green PCR Master Mix (Applied Biosystems, USA), and 0.5 μl each 10 μM gene-specific primer (S2 Table). Each reaction was performed in triplicates using a Roche Light Cycler 96 system (Switzerland) under the following program: 30 s at 95°C, 45 cycles of 5 s at 95°C, 30 s at 58°C, and 10 s at 72°C. The specificity of the primer pairs was verified by the qPCR dissociation curve and further confirmed by PCR visualized on a 1.5% agarose gel. Each sample was tested in three biological replicates. The relative expression levels were calculated using the comparative C_t_ (2^-ΔΔCt^) method. The housekeeping gene *Actin* (*CsAct1*, Csa6M484600), which was stably expressed [35], was used as a standard control.

### Fatty acid profiling by gas chromatography-mass spectrometry (GC-MS)

The preparation of fatty acid methyl esters (FAMEs) from cucumber seedlings was performed as described previously [36]. Briefly, approximately 0.2 g of leaf and root samples were ground in liquid nitrogen. Then, 1.0 ml of 10% KOH in methanol was added to each tissue sample and heated at 80°C for 2 h without shaking. After the mixture was cooled to room temperature and 0.5 ml of 50% HCl was added, the mixtures were extracted twice with 1.0 ml hexane and dried under a N_2_ stream. Each sample was then resuspended with 1.0 ml 3 N HCl in methanol, heated at 80°C for 2 h and then cooled to room temperature. After the addition of 1.0 ml 0.9% NaCl solution, the FAMEs were re-extracted in 1.0 ml hexane, transferred to glass GC vials (Agilent) and dried under N_2_. The FAMEs were then resuspended in 0.5 ml hexane for GC-MS analysis. 100 μg of heptadecylic acid (17:0, Sigma-Aldrich, USA) was added to each sample prior to the FAME extraction as an internal standard. Each sample was tested in three biological replicates.

The FAME samples were analyzed using a GC-MS system (QP2010 Plus, Shimadzu, Japan). The GC was equipped with anSP^™^-2560 column (100 m×0.25 mm, 0.2-μm phase thickness, Supelco, USA). One microliter of each sample was injected onto the column using a split/splitless injector maintained at 240°C, with a split ratio of 20:1. The column oven temperature was maintained at 150°C for 2 min, then raised to 230°C at a rate of 4°C min^-1^, and finally maintained at 230°C for 5 min. Helium was used as the carrier gas at a constant flow rate of 0.8 ml/min. Mass spectra were acquired in the electron ionization mode (ESI, 70 eV) from 45 to 500 Da at a rate of 1 scan s^-1^.

## Results and Discussion

### Identification of the *FAD* genes in cucumber

To identify the orthologous genes encoding FADs in cucumber, the full-length amino acid sequences of the AtFAD proteins (S1 Table) were blasted against the cucumber genome (v2.0) using the tBlastn algorithm with an *E*-value cut-off of 1E-5. In total, 23 putative *FAD* genes were identified in the cucumber genome with reliable sequencing data and gene structures. All identified cucumber *FAD* genes were named according to their similarity with their characterized counterparts in Arabidopsis (Table 1). These cucumber *FAD* genes included three *CsFAB2*s, two *CsFAD2*s, fourteen *CsFAD5*s, and one each *CsFAD3*, *CsFAD4*, *CsFAD6* and *CsFAD7*.

**Table 1:** tBlastn result using AtFAD family proteins as queries.

Query^a^	ICuGI No.	Chr. Localization	Direction	Designation	tBlastn^b^	
					Ident. (%)	*E*-Value
AtFAB2	Csa2M351530	Chr2:16086978..16090638	R	CsFAB2.1	81.54	0
	Csa2M083750	Chr2:6794468..6798154	F	CsFAB2.2	78.16	0
	Csa6M338660	Chr6:15509457..15511407	R	CsFAB2.3	65.40	1×10^−174^
AtFAD2	Csa3M808360	Chr3:30921555..30925121	R	CsFAD2.1	78.33	0
	Csa4M286360- Csa4M286350	Chr4:11057253..11058771	R	CsFAD2.2	59.27	9×10^−167^
AtFAD3	Csa1M532210	Chr1:18732851..18736154	R	CsFAD3	73.33	0
AtFAD4	Csa3M077710	Chr3:4196862..4198299	F	CsFAD4	62.59	5×10^−136^
AtFAD5/AtADS3	-	Scaffold002219:1..393 Scaffold000339:1..1..2040	R	CsFAD5.1	65.76	1×10^−178^
	Csa1M227450	Chr1:11921967..11924652	F	CsFAD5.2	63.47	6×10^−135^
	Csa1M227460	Chr1:11936523..11939129	F	CsFAD5.3	61.99	1×10^−127^
	Csa4M006050	Chr4:900644..902513	R	CsFAD5.4	79.27	7×10^−175^
	Csa4M006060	Chr4:906424..908374	F	CsFAD5.5	63.97	7×10^−135^
	Csa4M006070- Csa4M006080	Chr4:909500..913000	F	CsFAD5.6	78.39	5×10^−132^
	Csa4M006090- Csa4M006100	Chr4:915100..917420	F	CsFAD5.7	64.34	6×10^−138^
	Csa4M006120	Chr4:920341..922912	F	CsFAD5.8	61.17	3×10^−130^
	Csa4M006130	Chr4:929912..932466	F	CsFAD5.9	59.04	4×10^−126^
	Csa4M006140	Chr4:932850..935843	R	CsFAD5.10	66.54	6×10^−142^
	Csa4M006150	Chr4:940689..943196	R	CsFAD5.11	62.87	1×10^−128^
	Csa4M006160	Chr4:948297..951751	R	CsFAD5.12	64.47	6×10^−123^
	Csa7M376370	Chr7:13776214..13778880	R	CsFAD5.13	63.43	7×10^−128^
	Csa7M377870	Chr7:13789731..13792227	R	CsFAD5.14	61.17	3×10^−123^
AtFAD6	Csa1M255170	Chr1:12496633..12501089	F	CsFAD6	73.83	0
AtFAD7	Csa5M207960	Chr5:9235259..9237875	R	CsFAD7	69.11	0

^a^The accession numbers for AtFADs were listed in S1 Table.

^b^Ident., Identitiy.

**Fig 1:**
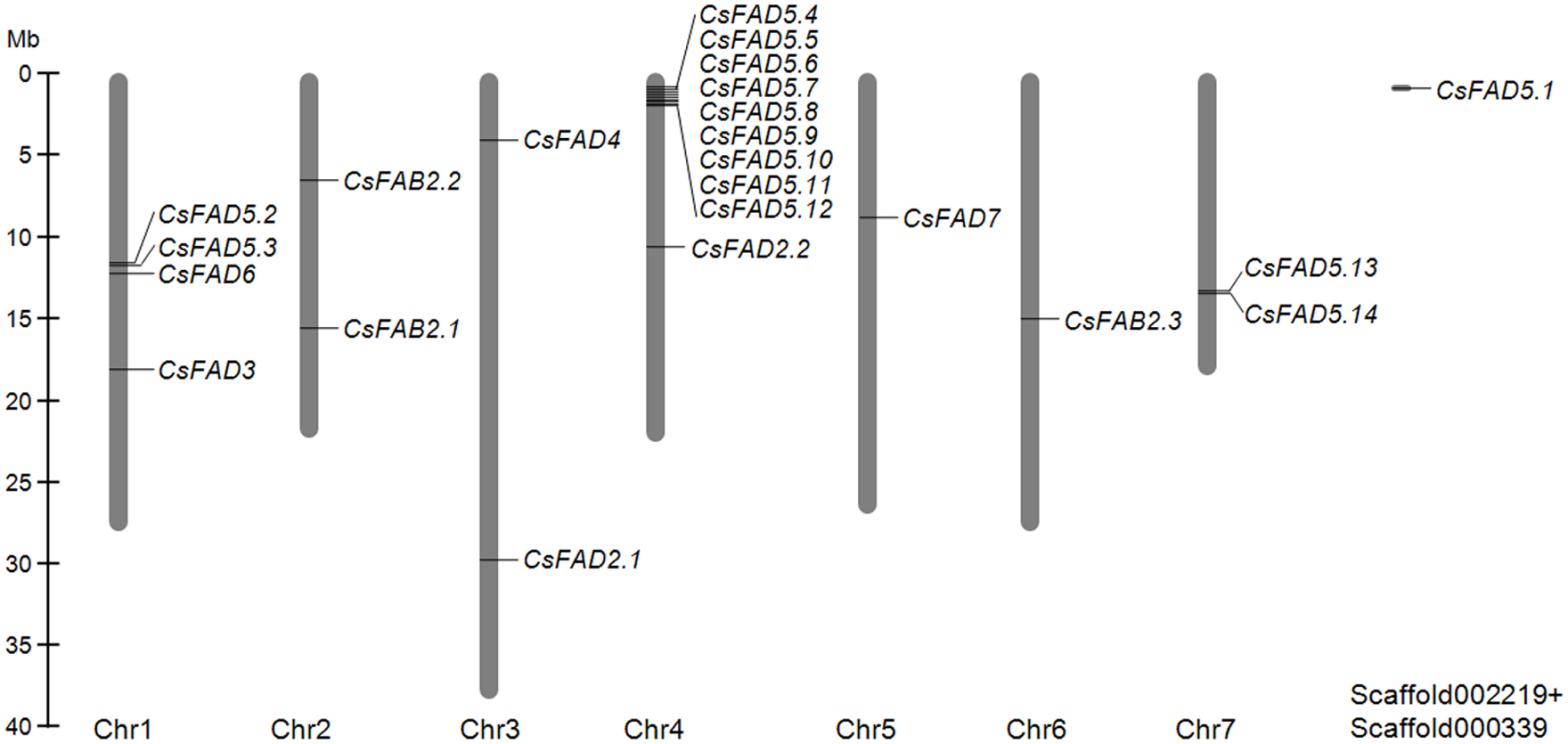
Chromosome localization of the cucumber *FAD* genes. The chromosome numbers are indicated below each vertical bar, and the scale of the chromosomes is in megabases (Mb).

Of the 23 cucumber *FAD* genes, 22 were mapped to the seven chromosomes in cucumber, but *CsFAD5*.*1* could not be mapped to any chromosome (Fig 1). The mapped genes were distributed unevenly among the chromosomes. Chromosomes 5 and 6 contained one *CsFAD* gene each, chromosomes 2, 3 and 7 contained two each, four *CsFAD* genes were located on chromosome 1, and up to ten *CsFAD* genes were found on chromosome 4. Two duplicated gene pairs (*CsFAD5*.*2*/*CsFAD5*.*3* and*CsFAD5*.*13*/*CsFAD5*.*14*) and a gene cluster *CsFAD5*.*4*~*CsFAD5*.*12* were found in tandem on chromosomes 1, 7 and 4, respectively, suggesting that some segmental and tandem duplication events may be responsible for the expansion of the *CsFAD5* genes.

The annotations of these cucumber *FAD* genes were manually verified and improved, and the annotated nucleotide sequences and the deduced amino acid sequencesare present in the S1 Fig. The protein sequences encoded by the cucumber*FAD* genes varied in length from 287 aa for CsFAD5.4 to 445 aa for CsFAD7. The predicted molecular weights (Mws) of the CsFAD proteins ranged from 33.47 kDa to 51.35 kDa, and the theoretical isoelectric points (p*I*s) ranged from 6.39 to 10.19 (Table 2). However, the lengths of the CsFAD5 proteins were highly conserved, varying from 287 aa to 297 aa, with the exception of CsFAD5.1, which was 380 aa long and much larger than the other CsFAD5 proteins (Table 2).

**Table 2:** Predicted protein characteristics of the cucumber FADs.

Designation	Length (aa)	Mw (kDa)	p*I*	Predicted subcellular localization		
				Localization	SP-length (aa)	Score
CsFAB2.1	396	46.43	6.39	C	33	0.702
CsFAB2.2	395	46.18	6.66	C	54	0.845
CsFAB2.3	388	44.11	6.44	C	34	0.596
CsFAD2.1	382	43.97	8.65	-	-	-
CsFAD2.2	387	45.40	9.18	-	-	-
CsFAD3	371	42.90	9.30	-	-	-
CsFAD4	313	34.69	7.66	C	32	0.531
CsFAD5.1	380	43.83	9.33	C	15	0.726
CsFAD5.2	297	35.22	9.31	-	-	-
CsFAD5.3	295	35.14	9.12	-	-	-
CsFAD5.4	287	33.47	8.61	-	-	-
CsFAD5.5	292	34.26	9.75	M	13	0.752
CsFAD5.6	291	34.01	10.02	-	-	-
CsFAD5.7	294	34.86	8.85	-	-	-
CsFAD5.8	294	34.53	7.49	-	-	-
CsFAD5.9	293	34.69	10.16	-	-	-
CsFAD5.10	294	34.58	9.94	-	-	-
CsFAD5.11	296	34.94	10.19	-	-	-
CsFAD5.12	298	35.14	10.16	-	-	-
CsFAD5.13	296	35.02	9.91	-	-	-
CsFAD5.14	297	34.92	9.91	-	-	-
CsFAD6	443	51.35	9.41	-	-	-
CsFAD7	445	50.84	7.48	C	71	0.580

We further performed a bioinformatic analysis to predict the subcellular localization of the cucumber FAD proteins using the TargetP 1.1 and ChloroP 1.1 algorithms [28–30]. Consistent with their participated metabolic pathways, six cucumber FAD proteins with a high-confidence chloroplast signal peptide (SP) in their N-termini (CsFAB2.1, CsFAB2.2, CsFAB2.3, CsFAD4, CsFAD5.1 and CsFAD7) were predicted to localize to the chloroplast (Table 2). Unexpectedly, a mitochondrial targeting peptide was predicted in the N-terminus of CsFAD5.5, suggesting that this CsFAD5 isoform might exhibit functions in the mitochondria. A potential mitochondria-targeted FAD was also reported in cacao (TcSAD3), with a similar mitochondrial targeting peptide being predicted at its N-terminus [11]. No SP was detected in the other cucumber FAD5 proteins (Table 2), indicating the potential cytoplasmic localization or other subcellular localizations for these FAD5 proteins. Actually,the divergent subcellular localization of FAD5 proteins had been reported in Arabidopsis. AtFAD5 was identified as a plastid protein, while two other Arabidopsis FAD5s (AtADS1 and AtADS2) were present as components of the ER membranes [37]. In plant, the alternative subcellular targeting of FAD5 proteins might switch the enzyme specificity. For example, the Δ^7^-regiospecificity of AtFAD5 protein was identified to be attributed to plastidial targeting of the enzyme rather than the numerous sequence differences within the catalytic portion of the enzymes. When the plastidial FAD5 was retargeted to the cytoplasm, regiospecificity shifted 70-fold, Δ^7^ to Δ^9^. Conversely, retargeting of the cytoplasmic desaturases to the plastid, shifted regiospecificity about 25-fold, Δ^9^ to Δ^7^ [38]. In cucumber, the multiple targeting of different CsFAD5 isoformsmight also create different metabolites in alternate compartments.

### Phylogenetic analysis of the cucumber *FAD* genes

**Fig 2:**
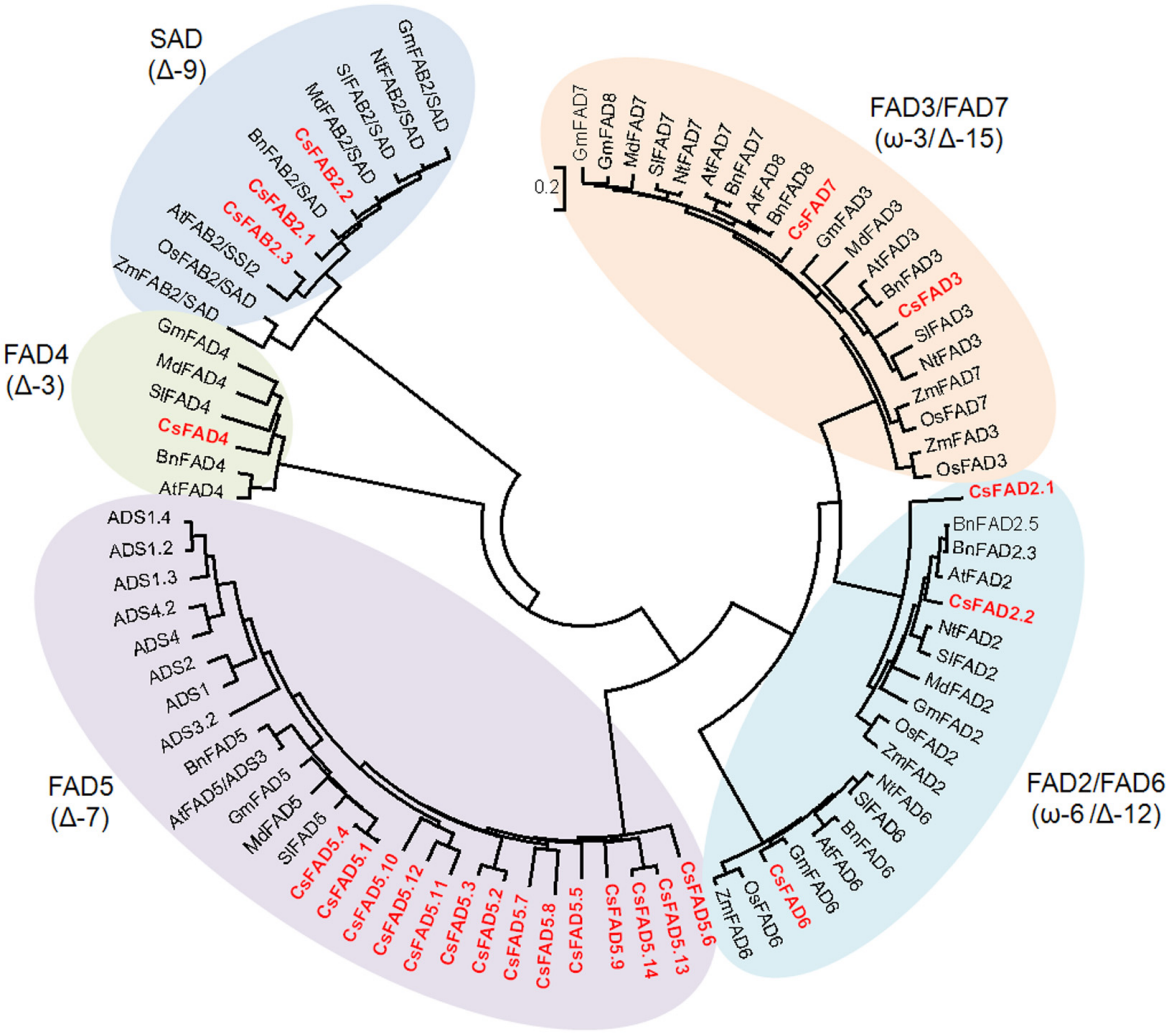
Phylogenetic tree depicting the relationships among the plant FAD proteins. The FAD proteins from Arabidopsis (*A*. *thaliana*, AtFADs), rape (*Brassica napus*, BnFADs), soybean (*G*. *max*, GmFADs), apple (*Malus domestica*, MdFADs), tomato (*Solanum lycopersicum*, SlFADs), tobacco (*Nicotiana tabacum*, NtFADs), rice (*Oryza sativa*, OsFADs), and maize (*Zea mays*, ZmFADs) were aligned using ClustalX 2.0, and the phylogenetic tree was constructed using the Neighbor-Joining (NJ) method with the program MEGA 5.2. The cucumber FAD proteins are highlighted in red.

To evaluate the phylogenetic relationships of the cucumber FAD proteins with other different plant FADs, an NJ-phylogenetic tree was constructed according to the protein sequence alignments of a set of FAD family members from other plant species, including cucumber, Arabidopsis, rape, soybean, apple, tomato, tobacco, and two monocot plants (rice and maize). As shown in Fig 2, the corresponding tree divided these FAD proteins into five subfamilies.

The plant FAB2 proteins, also named SADs, introduce a double bond into the Δ-9 position of stearoyl-ACP (18:0-ACP)[3]. In the phylogenetic tree, the FAB2 proteins from different plants formed a separate subfamily. The CsFAB2.1 and CsFAB2.3 isoforms were clustered closely to the FAB2 proteins from Arabidopsis and rape, whereas CsFAB2.2 was closer to MdFAB2 from apple (Fig 2).

The other two subfamilies included the FAD4 and FAD5 proteins (Fig 2). FAD4 and FAD5 introduce the first double bond into a saturated acyl chain at the Δ-3 or Δ-7 position, respectively [3]. Compared to the FAD5 subfamily in Arabidopsis(nine members) [37–40], the cucumber FAD5 subfamily was much larger (fourteen members). Furthermore, of the fourteen CsFAD5 members, CsFAD5.1 and CsFAD5.4 grouped with the other plant FAD5 proteins, whereas the remaining twelve CsFAD5 proteins clustered together, indicating that potential gene duplication events during the expansion of the CsFAD5 subfamily occurred after cucumber split from other plants.

The FAD2/FAD6 subfamily included the FAD2 and FAD6 proteins, which are frequently called ω-6 or Δ-12 desaturases, and generally introduce the second double bond between an existing double bond and the acyl end [3]. In this subfamily, the FAD2 and FAD6 proteins grouped into two distinct clades, representing the ER- and plastid-type ω-6 FADs (Fig 2). In each clade, the FAD2 or FAD6 proteins from the monocot plants clustered with each other and separately from their dicot homologs. For the two CsFAD2 isoforms, CsFAD2.1 clustered more closely with the FAD2 proteins from soybean and apple, which are close relatives of cucumber, but CsFAD2.2 was much closer to the FAD2 proteins from the *Brassicaceae* (Arabidopsis and rape).

All the FAD3, FAD7 and FAD8 proteins used in this study fell into the FAD3/FAD7 subfamily, which are also called ω-3 or Δ-15 desaturases and introduce a double bond after two existing double bonds [3]. Similar to the FAD2/FAD6 subfamily, FAD3 and FAD7/FAD8 grouped into separate clades that represented the ER- and plastid-type ω-3 FADs. The exceptions were the FAD3 and FAD7 proteins from the monocots, which were included in neither the FAD3 nor the FAD7 clade but clustered together to form a monocot branch. In the FAD7/FAD8 clade, soybean, Arabidopsis and rapeeach had one FAD7 and one FAD8, but in cucumber, only one CsFAD7 was included (Fig 2). However, it should be noted that according to their nomenclature or phylogenetic relationships, it was difficult to distinguish *FAD7* and *FAD8*, due to the high sequence similarity [14, 15].

### Conserved motifs in the cucumber FAD proteins

**Fig 3:**
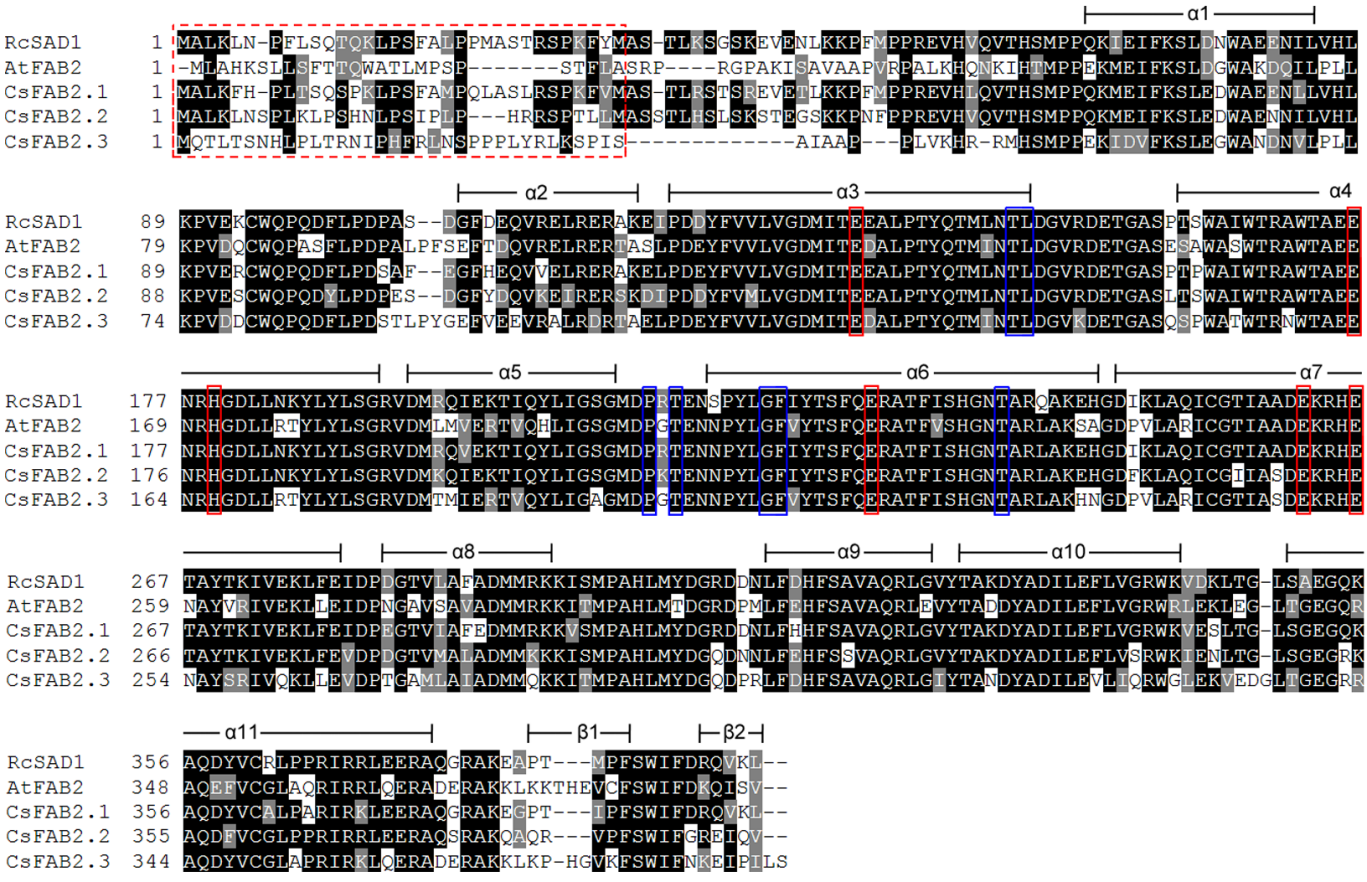
Multiple amino acid sequence alignment of the CsFAB2 proteins from cucumber and the SADs from Arabidopsis (AtFAB2) and castor (RcSAD1). The secondary protein structures of the SAD proteins were compared, deduced and annotated according to the crystal structure of RcSAD1. The sequence alignment was performed using ClustalX, followed by shading with Boxshade 3.21. The predicted chloroplastic localization signal peptide is indicated in the red and dashed box. The conserved amino acid residues involved in the binding of iron ions and substrate specificity are marked in red and blue boxes, respectively.

FAB2 is the soluble fatty acid desaturase, and its protein structure was analyzed separately from the membrane-bound desaturases in this study. The three-dimensional structure of the SAD protein from castor (RcSAD1) has been extensively studied [41–43]. To provide further insights into the protein catalytic activities of the CsFAB2 isoforms in cucumber, a multiple amino acid sequence alignment was conducted with RcSAD1, AtFAB2 and the three CsFAB2 proteins. As shown in Fig 3, these FAB2 proteins shared an overall high identity with each other. A high degree of variation was observed within the putative N-terminal chloroplast SP region. Consistent with the crystal structure of RcSAD1, all three CsFAB2 proteins consisted of eleven highly conserved α-helices and two β-sheets (Fig 3).

Notably, a further detailed investigation of the determinative residues also revealed that all three CsFAB2 isoforms shared a conserved di-iron center and similar substrate preferences. For example, six residues involved in iron binding (E105, E143, H146, E196, E229 and H237; positions assigned according to RcSAD1) were also conserved in all three CsFAB2 proteins (marked in red boxes in Fig 3). Additionally, the amino acid residues at positions 117, 118, 189 and 206 (according to RcSAD1) were reported to be crucial for the function of RcSAD1 as a Δ-9 18:0-ACP desaturase, and substitutions of these residues converted RcSAD1 into an enzyme that could function as a Δ-6 16:0-ACP desaturase [38, 39]. CsFAB2.1, CsFAB2.2 and CsFAB2.3 shared the same residues at these positions with RcSAD1 (marked in blue boxes in Fig 3). Likewise, at positions 179 and 181, which were crucial for the substrate specificity of RcSAD1 [44], all three CsFAB2 proteins contained the same residues as RcSAD1 and AtFAB2 (Fig 3). These results suggest that all three CsFAB2 proteins might exhibit the same enzymatic characteristics as RcSAD1 in castor. Given the fact that most plants lack other desaturases that utilize 18:0 as substrate, the activity of FAB2 is of particular interest because of its significant effects on the ratio of unsaturated fatty acids, and the relationship of this to the fluidity and rigidity of the membrane system [45]. The conservation between CsFAB2s and RcSAD1 also provided us the possibilities to design CsFAB2 with novel substrate specificity through site directed mutagenesis to modify the final fatty acid profiles in transgenic cucumber plants [45].

The other cucumber FAD proteins identified in this study belonged to the membrane-bound desaturase family. To reveal the conserved motifs in these membrane-bound FAD proteins, the detailed sequence alignments were performed against their Arabidopsis homologs (S2–S6 Figs). Similar to the Arabidopsis FADs, all the identified cucumber membrane-bound FAD proteins contained three histidine boxes (His-box; S2–S6 Figs), which are thought to be involved in the formation of a part of the di-iron center where oxygen activation and substrate oxidation occur [5, 46]. The amino acid residues in these His-boxes were highly conserved, especially for the CsFAD proteins in the same subfamily (Table 3), even for the FAD isoforms among different plant species (S2–S6 Figs). Additionally, the relative positions of these His-boxes were similar among the different CsFADs. The first and second His-boxes were located within 31 aa of each other. The third His box was located in the C-termini of the CsFAD proteins. However, the number of residues between His-box 2 and His-box 3 differed between FAD subfamilies (Table 3). For example, the length between the second and third His-box was 169 or 170 residues in the two CsFAD2 proteins and 162 residues in the two ω-3 CsFADs (CsFAD3 and CsFAD7). For CsFAD6, this length was 157 aa. In the CsFAD5 cluster, this length was almost identical, with 127 or 128 aa in all fourteen members. The exception was CsFAD4, in which the length between His-box 1 and His-box 2 was 53 aa, and only 24 aa separated His-box 2 and His-box 3 (Table 3).

**Table 3:** The conserved histidine boxes of the membrane-bound FAD proteins in cucumber.

Type	Protein	His-box 1		His-box 2		His-box 3	
		Sequence	Position	Sequence	Position	Sequence	Position
ω-6/Δ-12	CsFAD2.1	HECGH	116–120	HRRHH	152–156	HVIHH	327–331
	CsFAD2.2	HECGH	105–109	HRRHH	141–145	HVAHH	315–319
	CsFAD6	HDCAH	167–171	HDQHH	203–207	HIPHH	363–367
ω-3/Δ-5	CsFAD3	HDCGH	101–105	HRTHH	137–141	HVIHH	304–308
	CsFAD7	HDCGH	170–174	HRTHH	206–210	HVVHH	373–377
Δ-3	CsFAD4	QGHH	160–163	HAWAH	217–221	HSTHH	246–250
Δ-7	CsFAD5.1	HRNLSH	156–161	HRYHH	193–197	HNNHH	325–329
	CsFAD5.2	HRNLSH	68–73	HRCHH	105–109	HNNHH	237–241
	CsFAD5.3	HRNLSH	66–71	HRCHH	103–107	HNNHH	235–239
	CsFAD5.4	HRNLSH	63–68	HRYHH	100–104	HNNHH	232–236
	CsFAD5.5	HRNLAH	63–68	HRCHH	100–104	HNNHH	232–236
	CsFAD5.6	HRNLAH	64–69	HRCHH	101–105	HNNHH	234–238
	CsFAD5.7	HRNLSH	73–78	HRCHH	110–114	HNNHH	243–247
	CsFAD5.8	HRNLSH	64–69	HRYHH	101–105	HNNHH	234–238
	CsFAD5.9	HRNLAH	71–76	HRCHH	108–112	HNNHH	240–244
	CsFAD5.10	HRNLAH	63–68	HRYHH	100–104	HNNHH	232–236
	CsFAD5.11	HRHLTH	71–76	HRYHH	108–112	HNNHH	240–244
	CsFAD5.12	HRQLSH	72–77	HRIHH	109–113	HNNHH	242–246
	CsFAD5.13	HRNLAH	63–68	HRCHH	100–104	HNNHH	232–236
	CsFAD5.14	HRNLAH	62–67	HRCHH	99–103	HNNHH	231–235

The protein sequence alignments also revealed the conserved signals responsible for proper subcellular localization of FAD proteins. Consistent with the bioinformatic prediction (Table 2), a chloroplast SP was identified at the N-termini of the CsFAB2.1, CsFAB2.2, CsFAB2.3, CsFAD4, CsFAD5.1, CsFAD6 and CsFAD7 proteins (Fig 2 and S2–S6 Figs).

In plants, unsaturated fatty acids are also synthesized by an alternativeeukaryotic pathway, which are located within the ER [5]. In agreement with the potential locations their metabolic pathways occurred,FAD2 and FAD3 are known as ER-localized FADs. Maintenance of the ER-resident proteins is mediated by a C-terminal retention signal that is recognized by a cognate receptor and therefore leads to the retrograde transport of escaped proteins back to the ER [47]. Both FAD2 and FAD3 contain an ER retention signal. In FAD2, the ER retention signal consists of Φ-X-X-K/R/D/E-Φ (Φ are the large hydrophobic amino acid residues) at the C-terminus, such as “YNNKL” in AtFAD2 (S2 Fig) [48]. As expected, both CsFAD2.1 and CsFAD2.2 contained similar ER retention signals, with “YKNKL” in CsFAD2.1 and “FRNKL” in CsFAD2.2 (S2 Fig). For FAD3, an alternative ER retention signal was identified. AtFAD3 contains a functional prototypic dilysine ER signal at its C-terminus, “KSKIN” (S3 Fig) [48]. However, CsFAD3 lacked a similar retention signal. A tyrosine-rich signal, “YLYHY”, was identified at its C-terminus, which might serve as an ER retention signal (S3 Fig).

It has been reported that in Arabidopsis, FAD2 and FAD3 in the ER and FAD6 and FAD7/8 in the plastid can form FAD2-FAD3 and FAD6-FAD7/8 heterodimers, respectively [19]. These dimers can serve as metabolic channels in which 18:1 is converted to 18:3 without releasing a free 18:2 intermediate [19, 48]. In cucumber, the co-localization of CsFAD2 and CsFAD3 in the ER and CsFAD6 and CsFAD7 in the chloroplast hinted that the similar metabolic channels might also function in cucumber.

### Exon-intron organization of the cucumber *FAD* genes

**Fig 4:**
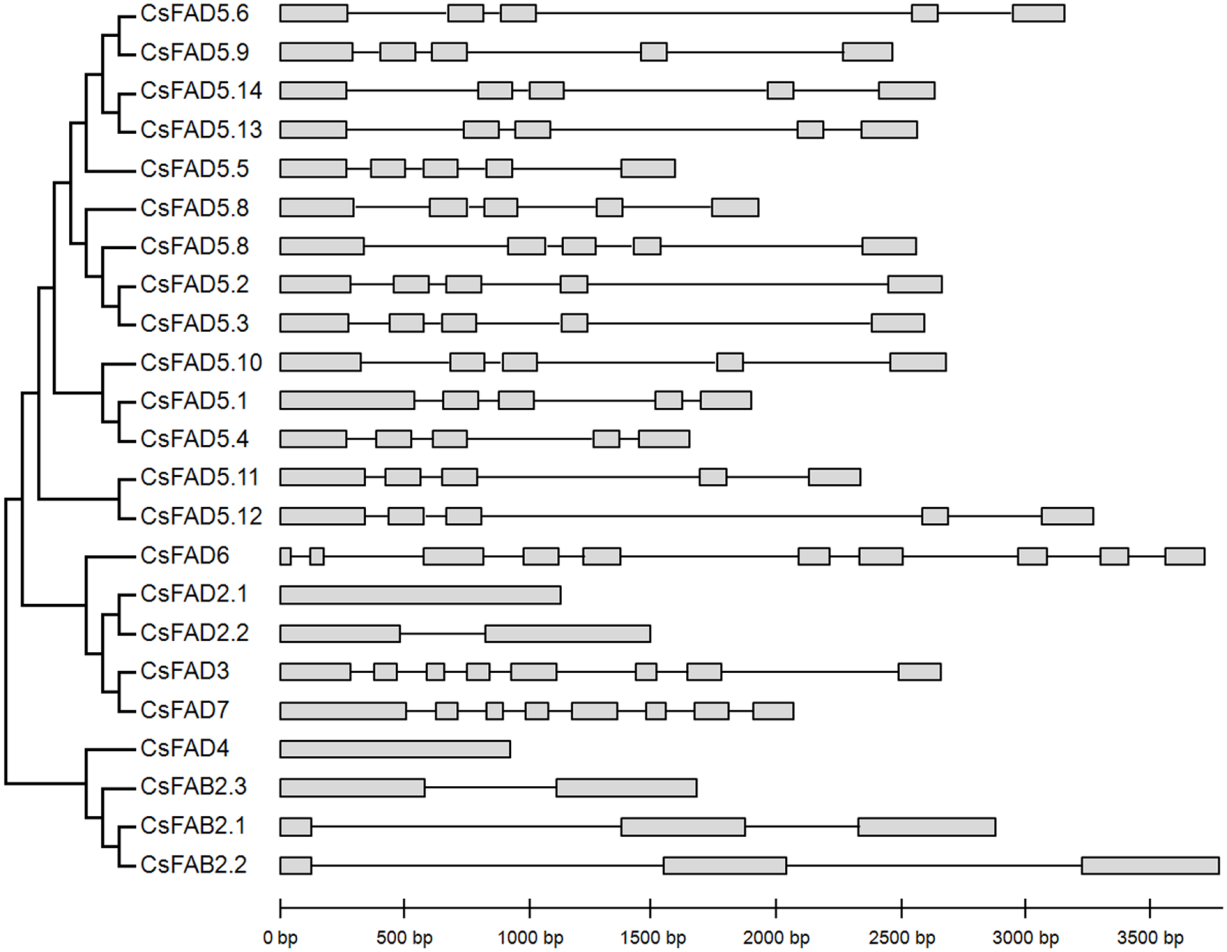
Phylogenetic relationships and gene structures of the cucumber *FAD* genes. Exons are represented by gray boxes and introns by black lines. The detailed sequence information for each exon and intron is in S1 Fig.

A separate phylogenetic tree was constructed using the protein sequences of all the identified cucumber *FAD* genes, and their exon-intron structures were compared (Fig 4). All members of the *CsFAD5* cluster contained five exons and four introns. Both *CsFAD3* and *CsFAD7*, which encode ω-3 FADs, contained eight exons. However, for the genes encoding the ω-6 FADs (*CsFAD2*.*1*, *CsFAD2*.*2* and *CsFAD6*), the exon-intron organization was quite different. *CsFAD6* had up to ten exons, whereas *CsFAD2*.*1* contained no intron in its open reading frame (ORF), and *CsFAD2*.*2* had a single intron (Fig 4). Similar to *CsFAD2*.*1*, no intron was contained in the ORF of *CsFAD4*. For the *CsFAB2* isoforms, two (*CsFAB2*.*1* and *CsFAB2*.*2*) had three exons, whereas *CsFAB2*.*3* contained only two exons. The exon-intron organization patterns of the *CsFAD* genes, as well as the lengths of the exons and introns in these genes, were highly conserved with those reported in other plant *FAD* genes, such as Arabidopsis, soybean, cotton and others [12, 14, 15]; these findings support a conserved evolutionary history among different plants.

### Duplication and divergence rate of the cucumber *FAD* genes

*CsFAB2*, *CsFAD2* and *CsFAD5* were identified as multi-families, including three, two and fourteen members, respectively (Table 1). According to their chromosome locations (Fig 1), phylogenetic relationships (Fig 3), as well as their flanking genes in the cucumber genome, the expansion of these gene families was possibly due to the segmental and/or tandem duplication events (Table 4). To explore the association of Darwinian selection in the duplication and divergence of the duplicated *FAD* genes, the *K*_a_/*K*_s_ ratios were estimated for these duplicated gene-pairs [33]. The *K*_a_/*K*_s_ ratios among the triple *CsFAB2* genes were estimated as > 1, suggesting that positive selection on these duplication events occurred during *CsFAB2* expansion (Table 4). Additionally, the segmental duplication events were estimated to have occurred around 8 mya and 19–20 mya for *CsFAB2*.*1*/*CsFAB2*.*2* and *CsFAB2*.*1*/*CsFAB2*.*3* pairs (Table 4). For the two copies of *CsFAD2*, the *K*_a_/*K*_s_ ratio was about 0.12, indicating that the segmentally duplicated *CsFAD2* genes were under strong purifying selection pressure. A much earlier divergence was observed from *CsFAD2*.*1*-*CsFAD2*.*2* around 202 mya, respectively (Table 4). The 14 *CsFAD5* genes were distributed into three gene clusters in the cucumber genome, and of them, CsFAD5.2/CsFAD5.3, CsFAD5.7/CsFAD5.8, CsFAD5.10/CsFAD5.12, and CsFAD5.13/CsFAD5.14 were clustered separately in the phylogenetic tree. The *K*_a_/*K*_s_ ratios for these tandem duplicated *CsFAD5* pairs ranged from 0.8031 to 1.2842 with an average of 0.99 (Table 4). It suggested that nearly neutral selections accompanied with the *CsFAD5* expansion.

**Table 4:** *K*_a_/*K*_s_ analysis and duplicated date calculation for duplicated *CsFAD* genes.

Duplicated pair	Duplication type	*K*_a_	*K*_s_	*K*_a_/*K*_s_	T (mya)
*CsFAB2*.*1/CsFAB2*.*2*	Segmental	0.3594	0.1079	3.3309	8.30
*CsFAB2*.*1/CsFAB2*.*3*	Segmental	0.5691	0.2477	2.2975	19.05
*CsFAB2*.*2/CsFAB2*.*3*	Segmental	0.5877	0.2670	2.2011	20.54
*CsFAD2*.*1/CsFAD2*.*2*	Segmental	0.3254	2.6347	0.1235	202.67
*CsFAD5*.*2/CsFAD5*.*3*	Tandem	0.0820	0.1021	0.8031	7.85
*CsFAD5*.*7/CsFAD5*.*8*	Tandem	0.2931	0.3595	0.8153	27.65
*CsFAD5*.*10/CsFAD5*.*12*	Tandem	0.3046	0.3315	1.0883	23.43
*CsFAD5*.*10/CsFAD5*.*11*	Tandem	0.3123	0.3130	0.9978	24.08
*CsFAD5*.*11/CsFAD5*.*12*	Tandem	0.1514	0.1582	0.9570	12.17
*CsFAD5*.*13/CsFAD5*.*14*	Tandem	0.0949	0.0739	1.2842	5.68

### Tissue specific expression patterns of the *FAD* genes in cucumber seedlings

To characterize the functions of the cucumber *FAD* genes, the expression profiles were analyzed. For the *CsFAD5* gene subfamily, CsFAD5.1 was the homolog of Arabidopsis FAD5, whose regio- and substrate specificity, and biological functions have been formally identified [37–39]. Therefore, *CsFAD5*.*1* was used as a representative gene, and the expression patterns of the other *CsFAD5* genes were not included in this study. Firstly, *in silico* expression analysis was performed by mining the EST, cDNA and PUT data in PlantGDB.The results showed that only *CsFAB2*.*1*, *CsFAD2*.*1*, *CsFAD3* and *CsFAD7* were supported by the expression evidences (Table 5). Then, the expression of cucumber *FAD* genes was further verified using the eight EST libraries released in ICuGI database. Interestingly, other than *CsFAD2*.*2*, all the cucumber *FAD* genes matched to at least four ESTs, and quite larger numbers of ESTs could be matched to the potentially expressed *CsFAD* genes identified from PlantGDB database (Table 5).

**Table 5:** *In silico* expression analysis of the cucumber *FAD* genes.

Gene	PlantGDB^a^			Number of ESTs in ICuGI^b^								
	EST	cDNA	PUTs	GenBank (1,113)	Fruit (1,392)	8 DPP fruit (187,276)	Leaf (717)	Gynoecious flower (171,786)	Hermaphrodite flower (149,364)	Female flower buds (716)	Male flower buds(912)	Total (513,276)
*CsFAB2*.*1*	+	+	+	2	1	40		33	34			110
*CsFAB2*.*2*	-	-	-		6			3	5			14
*CsFAB2*.*3*	-	-	-					10	3			13
*CsFAD2*.*1*	+	+	+		1	59	1	90	99	1	1	252
*CsFAD2*.*2*	-	-	-									0
*CsFAD3*	+	-	+		1	13		188	159		1	362
*CsFAD4*	-	-	-					3	1			4
*CsFAD5*.*1*	-	-	-			1		3	2			6
*CsFAD6*	-	-	-			5		6	2			13
*CsFAD7*	+	+	+	1		1		31	21	1		55

^a^ +, expressed; -, no blast hits with an *E* value ≤ 10^−4^.

^b^The number within parentheses indicates the total EST number in each library.

**Fig 5:**
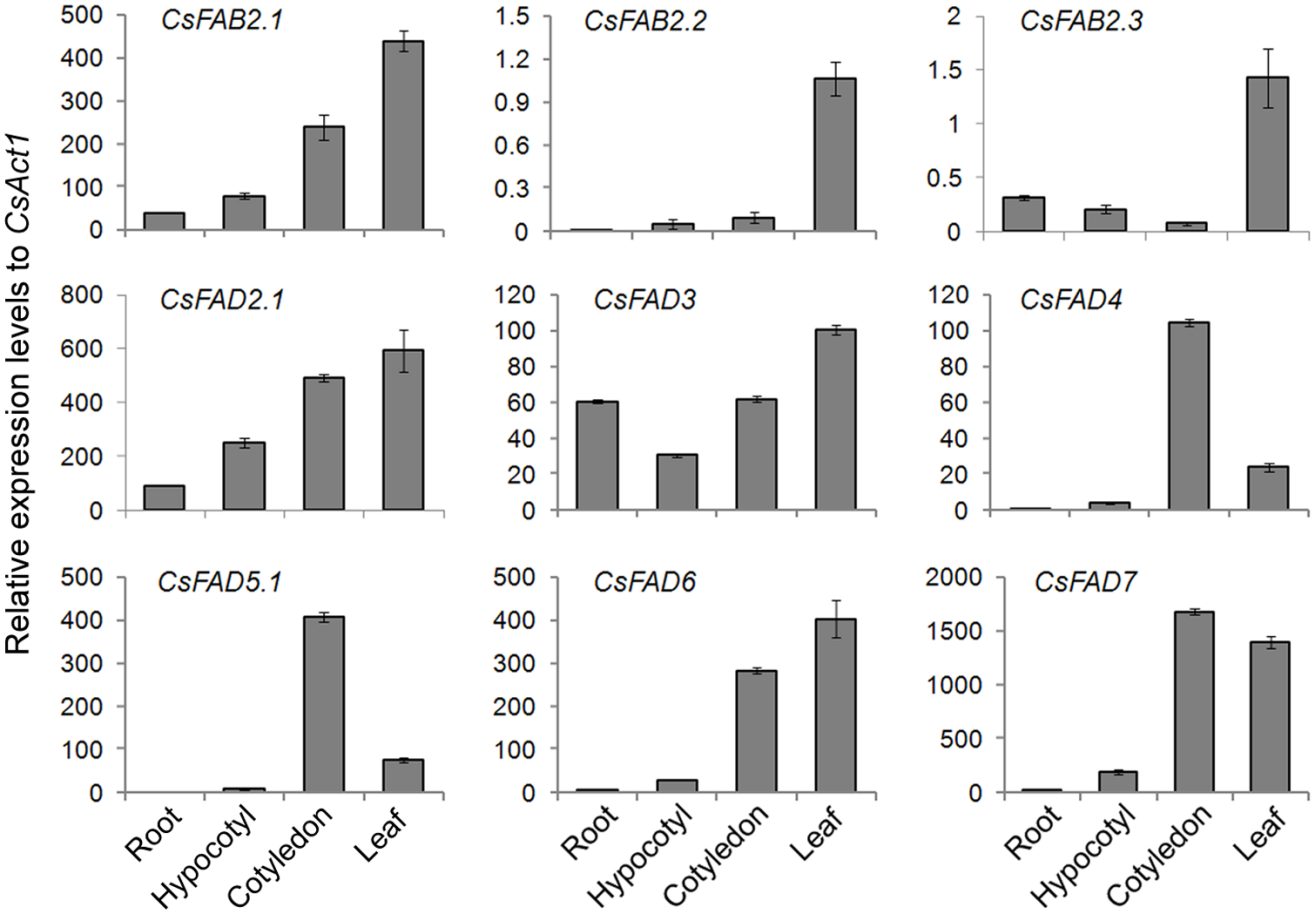
Expression patterns of the cucumber *FAD* genes in various tissues of cucumber seedlings. The bar charts illustrate the relative expression levels of the cucumber *FAD* genes in the various tissues, as measured by qPCR and normalized to *CsAct1* expression. The data represent the means of three independent replicates ± standard deviation (sd).

Furthermore, the expression profiles of *CsFAD* genes were examined by qPCR in various tissues of cucumber seedling, including the roots, hypocotyls, cotyledons and leaves. As shown in Fig 5, the expression patterns varied significantly in different cucumber tissues. Three *CsFAB2* genes were dominantly expressed in the cucumber seedling leaves. However, in comparison with the other two *CsFAB2* genes, *CsFAB2*.*1* showed much higher expression levels (approximately 500-fold) in all of the detected tissues. For *CsFAD2*.*1*, *CsFAD3* and *CsFAD6*, the highest expression levels were detected in the leaves, whereas for *CsFAD4*, *CsFAD5*.*1* and *CsFAD7*, the highest transcript abundances were detected in the cotyledons. In the roots and hypocotyls, only trace expression levels could be detected for any cucumber *FAD* gene except *CsFAD2*.*1* and *CsFAD3* (Fig 5), which indicate the central roles of these two CsFAD proteins in modulating fatty acid composition in the roots and hypocotyls of cucumber seedlings.

Consistent with the digital analysis, no transcript was detected for *CsFAD2*.*2* in all the tested tissues (Fig 5). As illustrated by the *K*_a_/*K*_s_ analysis, the *CsFAD2*.*1* and *CsFAD2*.*2* gene pair underwent a strong purifying selection after gene duplication (Table 4). One such selection fate was the pseudofunctionalization of the duplicate [49]. Here, that no expression of *CsFAD2*.*2*could be detected in both *in silico* and qPCR analysis (Table 5 and Fig 5), further supported this conclusion.

**Fig 6:**
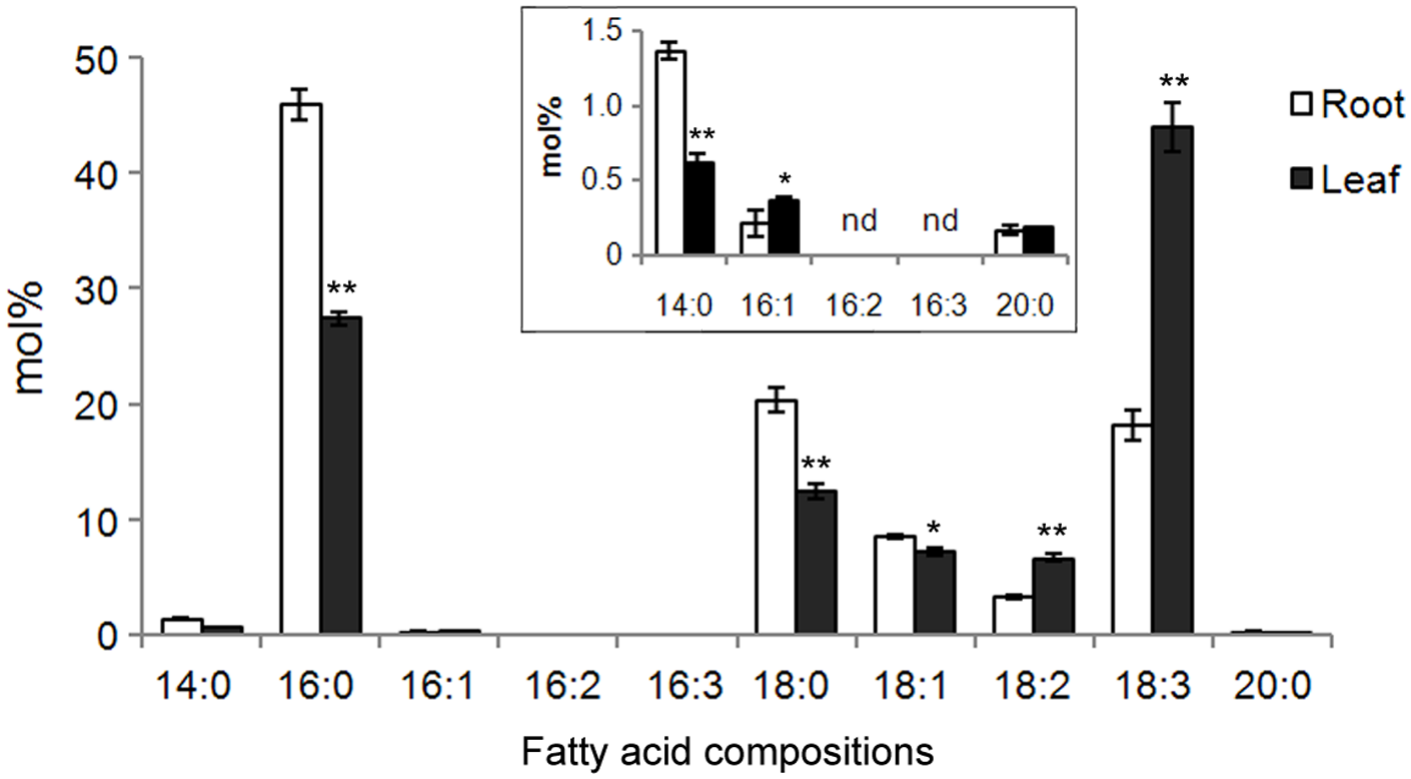
Fatty acid profiles in the roots and leaves of cucumber seedlings. The inset shows the enlargement of the fatty acid species below 1.5% (mol%). Fatty acids were determined by GC-MS, and the mean values (mol% ± sd) were calculated from three independent experiments. nd, not detected. *P< 0.05, **P< 0.01 compared to the corresponding value obtained in the roots.

Additionally, the fatty acid compositions in roots and leaves of cucumber seedlings were compared. As shown in Fig 6, neither 16:2 nor 16:3 could be detected in both leaves and roots. The major fatty acid in the roots was 16:0, which reached approximately 46% (mol%) of the total fatty acids. Lesser levels of other fatty acids were observed in the following order: 18:0>18:3>18:1>18:2>14:0>16:1>20:0. When compared with leaves, the relative levels (mol%) of saturated fatty acids (14:0, 16:0, and 18:0) were significantly higher in the roots, which is consistent with the lower *CsFAD* expression levels observed in roots (Fig 5). In leaves, the most abundant fatty acid species was 18:3, which represented approximately 44% of the total fatty acids therein. This result might be due to the extremely high expression levels of *CsFAD7* in cucumber seedling leaves (Fig 5). Another polyunsaturated fatty acid, 18:2, was also more abundant in the leaves than in the roots, also consistent with the higher expression of *CsFAD2*.*1* and *CsFAD6* in leaves (Fig 5). However, for 16:1, its level was slightly lower than that in roots, although its level was very low (< 0.5%) in both roots and leaves.These results indicated that *FAD* expression profiles might correlate well with fatty acid compositions in cucumber seedlings.

### Inducible expression profiles of the cucumber *FAD* genes

**Fig 7:**
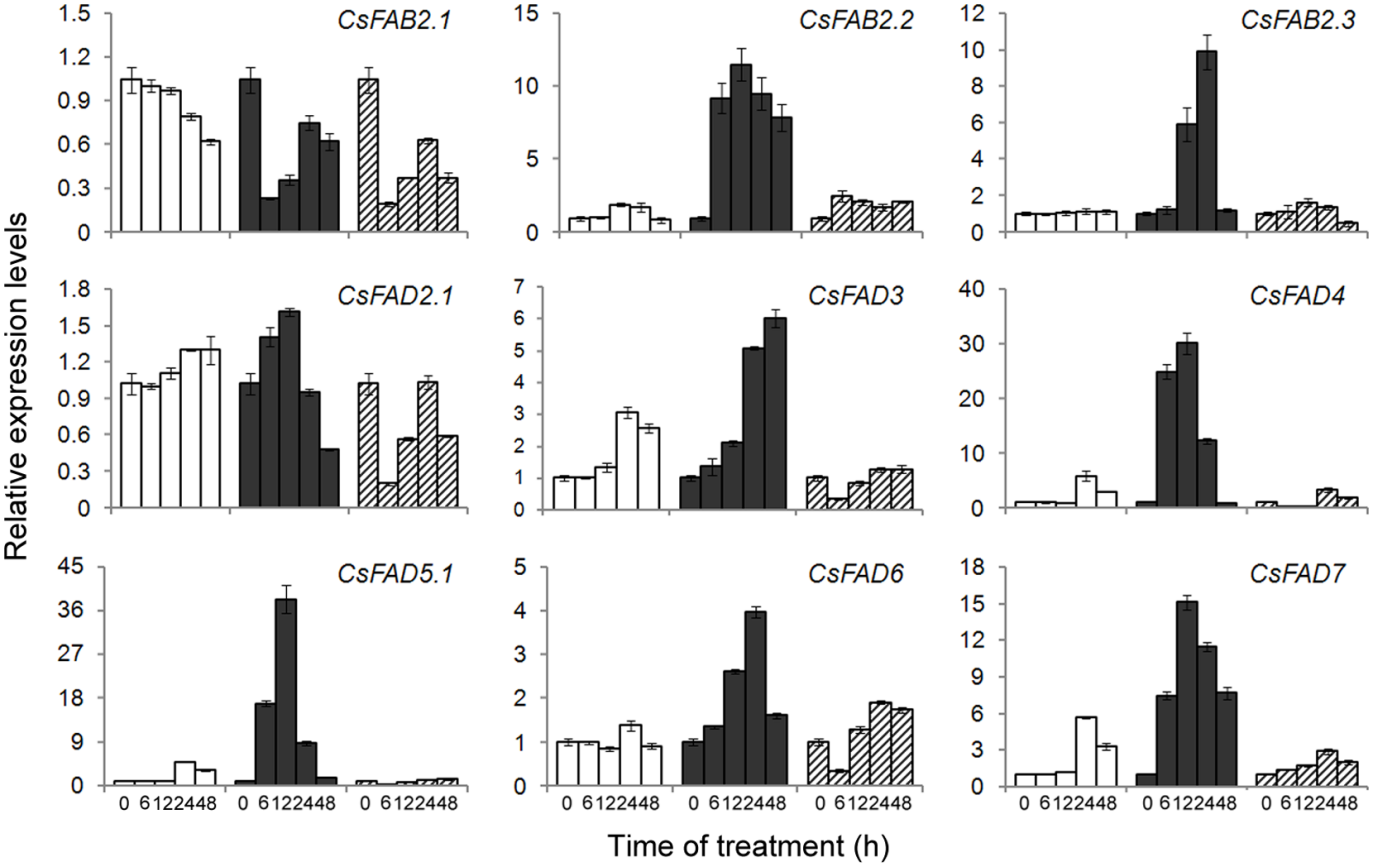
Expression profiling of the cucumber *FAD* genes in response to cold and heat stresses. The relative expression levels of the *FAD* genes in the leaves of cucumber seedlings treated with normal (28°C/18°C, white bars), cold (8°C/8°C, black bars) or hot (38°C/28°C, hatched bars) temperatures were determined by qPCR. The expression level for each gene in the control plants at 0 h was normalized to 1.0. The data represent the means of three independent replicates ± sd.

By qPCR, we also examined the inducible expression patterns of cucumber *FAD* genes in response to cold (8°C/8°C) or heat (38°C/28°C) stress. Upon cold treatment, most of the cucumber *FAD* genes were upregulated, with the exception of *CsFAB2*.*1*. Although *CsFAB2*.*1* was expressed at relatively high levels under normal conditions (Fig 5), under cold temperatures, an approximately 3-fold reduction was observed (Fig 7). For the other two *CsFAB2* isoforms, an approximate 10-fold increase was caused by cold stress, although with distinct inducible time courses (Fig 7). For *CsFAB2*.*2*, induction occurred throughout the experiment, whereas for *CsFAB2*.*3*, cold-induced upregulation occurred later (12 h) and peaked after 24 h of treatment. Subsequently, the induction diminished (Fig 7). For *CsFAD2*.*1*, only a slight induction (1.61-fold) was detected after 12 h of cold treatment, and thereafter, expression was reduced. For *CsFAD3*, expression was induced continuously within 48 h of cold treatment. The expression of *CsFAD4*, *CsFAD5*.*1* and *CsFAD7* showed a similar induction pattern under cold stress. Their expression was induced rapidly (within 6 h of treatment) upon cold stress and peaked after 12 h of treatment (Fig 7). Compared to the other *FAD* genes, *CsFAD6* was induced slowly and peaked to a lesser extent (3.97-fold) after 24 h of cold treatment. It should be noted that at 24 and 48 h in the control samples, the expression of *CsFAD3*, *CsFAD4*, *CsFAD5*.*1* and *CsFAD7* was upregulated, although the extent of upregulation was much less than that under cold stress. This induction might be the result of a lower temperature at night (18°C) compared with the day (28°C).

Under heat stress, most of the cucumber *FAD* genes (*CsFAB2*.*1*, *CsFAD2*.*1*, *CsFAD3*, *CsFAD4*, *CsFAD5*.*1* and *CsFAD6*) were repressed. Especially after 6 h of heat treatment, a sharp reduction was observed, although the extent of reduction differed from gene to gene (Fig 7). Subsequently, the expressionrecovered but still was significantly lower than the corresponding values in the control samples, indicating the heat-repressed expression of these cucumber *FAD* genes. For *CsFAB2*.*3*, a heat-induced reduction continued until 48 h of treatment. However, in the expression of *CsFAB2*.*2* and *CsFAD7*, different results were detected. For *CsFAB2*.*2*, a slight induction (approximately 2-fold) was detected after heat treatment, and for *CsFAD7*, 2.88- and 2.00-fold induction was found at 24 and 48 h of heat treatment, respectively. When compared to the control plants, this slight induction was much less, also indicating the heat-repressed expression of *CsFAD7*.

**Fig 8:**
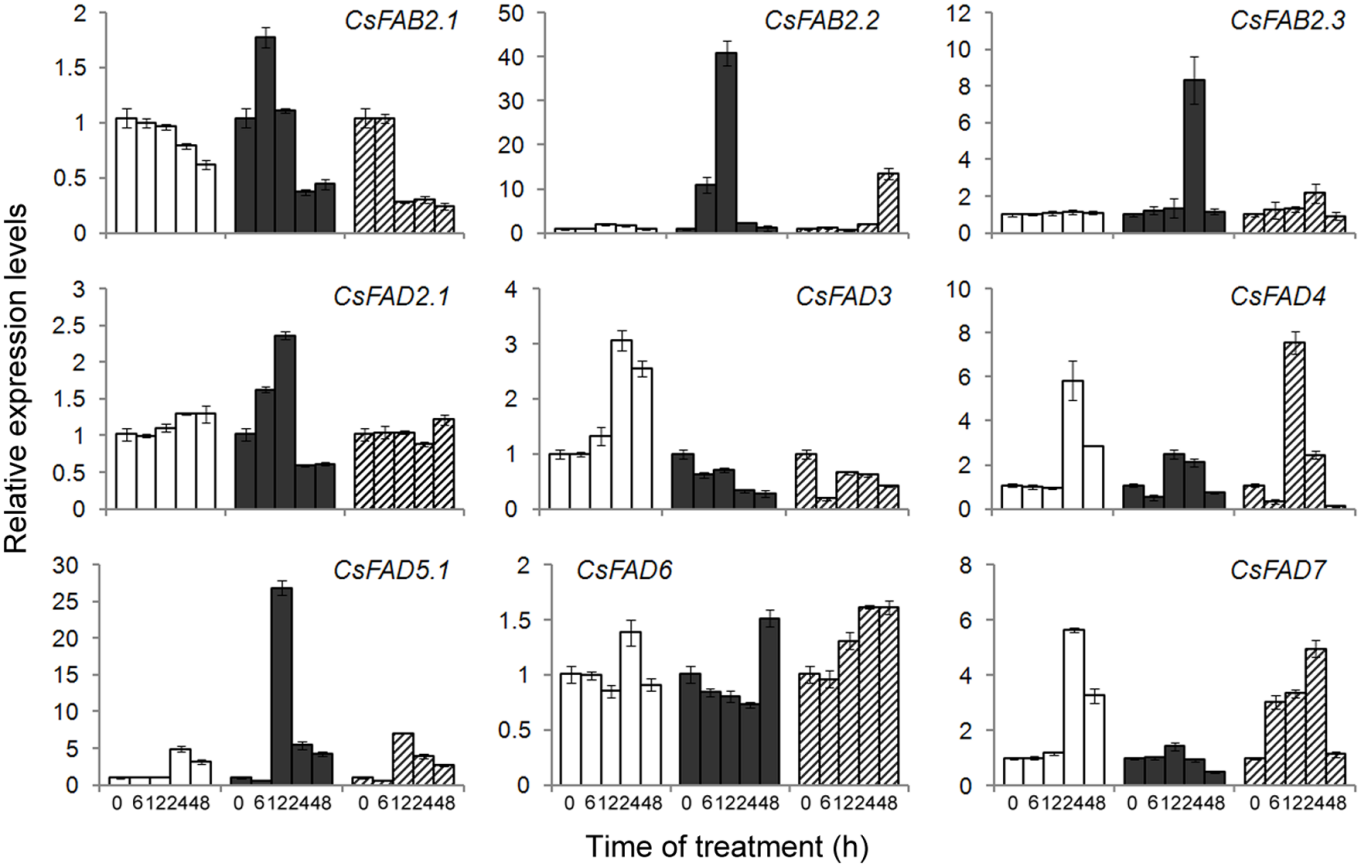
Expression profiling of the cucumber *FAD* genes in response to ABA and H_2_O_2_. The relative expression levels of the *FAD* genes in the leaves of cucumber seedlings treated without (white bars) or with ABA (100 μM, black bars) or H_2_O_2_ (1 mM, hatched bars) were determined by qPCR. The expression level for each gene in the control plants at 0 h was normalized to 1.0. The data represent the means of three independent replicates ± sd.

ABA and H_2_O_2_ serve as the universal signals during abiotic stress responses [50]. To determine whether the cold- and heat-induced changes in the expression of cucumber *FAD* genes were due to the applied stress itself or associated with stress-related plant signals, we also analyzed the *FAD* transcript levels in cucumber seedlings treated with exogenous ABA and H_2_O_2_. Upon ABA application, the expression of these cucumber *FAD* genes differed from one another. For *CsFAB2*.*1*, a transient and slight induction (1.78-fold) was observed 6 h after ABA treatment, and then its expression was significantly repressed. For *CsFAB2*.*2* and *CsFAB2*.*3*, their expression levels peaked at a 40.76-fold induction at 12 h and an 8.30-fold inductionat 24 h after ABA treatment, respectively(Fig 8). A sharp induction was also observed in *CsFAD5*.*1*, the expression of which was greatly induced (26.85-fold) 12 h after ABA treatment. Thereafter, *CsFAD5*.*1* expression was repressed rapidly (Fig 8). For *CsFAD2*.*1*, its expression was induced gradually and slightly within 12 h of treatment. The expression of the two ω-3 FAD-encoding genes (*CsFAD3* and *CsFAD7*) mirrored the ABA-repressed patterns. At the end of the time course, approximately 0.5-fold expression was detected (Fig 8). For *CsFAD6*, an approximately 1.5-fold induction 24 h after ABA treatment in the control seedlings was delayed to 48 h after ABA treatment (Fig 8).

As shown in Fig 8, upon H_2_O_2_ treatment, a significant reduction was detected in the expression of *CsFAB2*.*1*12 h after ABA treatment. However, for *CsFAB2*.*2*, H_2_O_2_ induced increased transcription until the end of the experiment (48 h). For *CsFAB2*.*3* and *CsFAD2*.*1*, no significant change was observed during the experiment (Fig 8). The H_2_O_2_-repressed pattern was also observed in *CsFAD3*, the expression of which was reduced rapidly after 6 h of treatment. For *CsFAD4* and *CsFAD5*.*1*, a transient induction (approximately 7-fold) occurred after 12 h of H_2_O_2_ treatment. For *CsFAD6*, only a slight induction was observed, and for *CsFAD7*, its expression increased gradually during the 24-h H_2_O_2_ treatment (Fig 8).

Taken together, these results from qPCR suggested that the expression of most cucumber *FAD* genes was induced by cold treatment but repressed by heat temperatures, consistent with the *FAD* genes from other plants [13–15, 51]. And this regulation by temperatures might be fulfilled, partially at least, via an ABA-dependent pathway, while the regulatory roles of H_2_O_2_ was limited (Fig 8). Besides the transcriptional regulation, some post-transcriptional regulation also occurred during the *FAD* genes expression. For example, in Arabidopsis, the C-terminal 44 aa of the FAD8 enzyme specifically destabilize the protein at high temperatures, even in the absence of reduced transcript accumulation [52]. In soybean, the phosphorylation of S185 in FAD2-1 may downregulate enzyme activity under high temperatures [53]. For *CsFAD3* and *CsFAD4*, a rapid reduction (within 6 h) after heat treatment was detected (Fig 7), implying that the similar post-transcriptional regulation might exist during the expression of these cucumber *FAD* genes, although further efforts should be needed.

The differential expression also indicated the potential roles of FADs in modulating the fatty acid compositions in cucumber seedlings under temperature stresses. Fatty acid desaturation is one of the factors involved in temperature responses and stress adaptions. Cold always led to increased fatty acid desaturation, while heat stress drastically decreased the fatty acid desaturation [23, 51, 54–56]. However, in cucumber, fatty acid desaturation showed a biphasic response to cold stress, with a decrease at the early responsive phase and then a dramatic increase at the late phase [57]. Thisresult was not completely consistent with our findings that the expression of cucumber *FAD* genes was continuously induced by cold treatment (Fig 7). This conflict could partically be explained by the fatty acid hydroperoxidation mediated by lipoxygenase (LOX). LOX could be induced by cold, and utilize polyunsaturated fatty acids (18:2 and 18:3) as the primary substrate. The LOX-dependent depletion of polyunsaturated fatty acids thereby decreased the level of fatty acid unsaturation [57]. To comprehensively decipher the complex metabolic and enzymatic changes involved in regulating fatty acid conpositions under temperature stresses in cucumber seedlings, some lipidomic and transcriptomic methods would be employed.

## Conclusions

In conclusion, the 23 cucumber *FAD* genes are comprehensively described, including their gene structures, phylogenetic profiles, subcellular localizations, conserved protein motifs, and expression patterns. These cucumber FADs were phylogenetically clustered with their counterparts from other plants. The detailed sequence alignment further revealed that the cucumber FAD proteins shared high amino acid sequence conservation and key determinative residues with the known plan FAD proteins. Also, the exon-intron structures of *FAD* genes were highly conserved in each subfamily. On the transcriptional level, the expression of cucumber *FAD* genes is positively correlated with the contents of unsaturated fatty acids. The cold-induced and heat-repressed expression patterns of the *FAD* genes suggest the potential roles of FADs in cucumber responses to temperature stresses. The characterization of these *FAD* genes aids the construction of the pathways involved in unsaturated fatty acid biosynthesis and provides candidate genes for the genetic engineering of stress tolerance in cucumber.

## Supporting Information

S1 Table FAD proteins used in sequence alignment and phylogenetic analysis. (DOCX)

S2 Table Primers used for qPCR analysis of cucumber *FAD* genes. (DOCX)

S1 Fig Nucleic acid sequences and the deduced amino acid sequences of 23 cucumber *FAD* genes. The intron sequences are shown in lowercase letters.
(PDF)

S2 Fig Sequence alignment of AtFAD2 and CsFAD2 proteins. The alignment was performed using Clustal X, followed by shading with Boxshade 3.21. The gaps are indicated as dashes. The conserved His-boxes are indicated with blue boxes, and the ER-retention signal is marked with red box.
(DOCX)

S3 Fig Sequence alignment of AtFAD3, AtFAD7, CsFAD3 and CsFAD7 proteins. The alignment was performed using Clustal X, followed by shading with Boxshade 3.21. The gaps are indicated as dashes. The conserved His-boxes are indicated with blue boxes, and the ER-retention signal of FAD3 proteins is marked with red box. The predicted chloroplast signal peptide of FAD7 proteins is highlighted with red dashed box.
(DOCX)

S4 Fig Sequence alignment of AtFAD4 and CsFAD4 proteins. The alignment was performed using Clustal X, followed by shading with Boxshade 3.21. The gaps are indicated as dashes. The conserved His-boxes are indicated with blue boxes, and the predicted chloroplast signal peptide of FAD4 is highlighted with red dashed box.
(DOCX)

S5 Fig Sequence alignment of AtFAD5 and CsFAD5 proteins. The alignment was performed using Clustal X, followed by shading with Boxshade 3.21. The gaps are indicated as dashes. The conserved His-boxes are indicated with blue boxes, and the predicted chloroplast signal peptide of FAD5 is highlighted with red dashed box.
(DOCX)

S6 Fig Sequence alignment of AtFAD6 and CsFAD6 proteins. The alignment was performed using Clustal X, followed by shading with Boxshade 3.21. The gaps are indicated as dashes. The conserved His-boxes are indicated with blue boxes, and the predicted chloroplast signal peptide of FAD6 is highlighted with red dashed box.
(DOCX)
