# Supplementary material for: Characterization of the Fatty Acid Desaturase Genes in Cucumber: Structure, Phylogeny, and Expression Patterns
Source: PLoS One. 2016 Mar 3;11(3):e0149917. doi: 10.1371/journal.pone.0149917 (PMC4777478; doi:10.1371/journal.pone.0149917)
Supplement: S1 Table — (DOCX) [file pone.0149917.s001.docx]

**Supporting Information**

**S1 Table. FAD proteins used in sequence alignment and phylogenetic analysis**.

| **Species** | **Protein** | **Accession** | **Protein** | **Accession** |
| --- | --- | --- | --- | --- |
| Arabidopsis (*A. thaliana*) | AtFAB2/SSI2 | AT2G43710 | AtADS1.4 | At1G06120 |
|  | AtFAD2 | AT3G12120 | AtADS2 | At2G31360 |
|  | AtFAD3 | AT2G29980 | AtADS3.2 | At3G15870 |
|  | AtFAD4 | AT4G27030 | AtADS4 | At1G06350 |
|  | AtFAD5/ADS3 | AT3G15850 | AtADS4.2 | At1G06360 |
|  | AtADS1 | At1G06080 | AtFAD6 | AT4G30950 |
|  | AtADS1.2 | At1G06090 | AtFAD7 | AT3G11170 |
|  | AtADS1.3 | At1G06100 | AtFAD8 | AT5G05580 |
| Oilseed rape  (*Brassica napus*) | BnFAB2/SAD | AAT65205 | BnFAD5 | CDY58467 |
|  | BnFAD2.3 | ACP39504 | BnFAD6 | AAT65203 |
|  | BnFAD2.5 | ACP39505 | BnFAD7 | ACS26170 |
|  | BnFAD3 | AAT09135 | BnFAD8 | ACS26172 |
|  | BnFAD4 | CDY14288 |  |  |
| Soybean  (*Glycine max*) | GmFAB2/SAD | AAA92462 | GmFAD5 | XP_006584522 |
|  | GmFAD2 | ACH43025 | GmFAD6 | XP_006574477 |
|  | GmFAD3 | NP_0012369430 | GmFAD7 | NP_001237361 |
|  | GmFAD4 | XP_003551889 | GmFAD8 | AEE25912 |
| Apple  (*Malus domestica*) | MdFAB2/SAD | XP_008370856 | MdFAD4 | XP_008385990 |
|  | MdFAD2 | XP_008344778 | MdFAD5 | XP_008373965 |
|  | MdFAD3 | XP_008385049 | MdFAD7 | NP_001280916 |
| Tobacco  (*Nicotiana tabacum*) | NtFAB2/SAD | AIC36757 | NtFAD6 | AIA22326 |
|  | NtFAD2 | AAT72296 | NtFAD7 | AIA22325 |
|  | NtFAD3 | P48626 |  |  |
| Tomato  (*Solanum lycopersicum*) | SlFAB2 | XP_004241001 | SlFAD5 | XP_004235954 |
|  | SlFAD2 | XP_004228665 | SlFAD6 | XP_004242585 |
|  | SlFAD3 | ABX24525 | SlFAD7 | NP_001234592 |
|  | SlFAD4 | XP_004244133 |  |  |
| Rice (*Oryza sativa*) | OsFAB2/SAD | AAP20854 | OsFAD6 | BAD09897 |
|  | OsFAD2 | ACN87220 | OsFAD7 | BAE79785 |
|  | OsFAD3 | BAA11397 |  |  |
| Maize (*Zea mays*) | ZmFAB2/SAD | NP_001151143 | ZmFAD6 | DAA48913 |
|  | ZmFAD2 | BAE93382 | ZmFAD7 | NP_001105303 |
|  | ZmFAD3 | NP_001149938 |  |  |
| Castor (*Ricinus communis*) | RcSAD1 | XP_002531889 |  |  |
