# Supplementary material for: Characterization of the Fatty Acid Desaturase Genes in Cucumber: Structure, Phylogeny, and Expression Patterns
Source: PLoS One. 2016 Mar 3;11(3):e0149917. doi: 10.1371/journal.pone.0149917 (PMC4777478; doi:10.1371/journal.pone.0149917)
Supplement: S2 Table — (DOCX) [file pone.0149917.s002.docx]

**Supporting Information**

**S2 Table. Primers used for qRT-PCR analysis of cucumber *FAD* genes.**

| **Gene** | **Primer sequence** | |
| --- | --- | --- |
| *CsFAB2.1* | CsFAB2.1-F  CsFAB2.1-R | 5’-AGTTGCCGTCCTTTGCGATG-3’  5’-CACCTCTCCACTGGTTTCAAATG-3’ |
| *CsFAB2.2* | CsFAB2.2-F  CsFAB2.2-R | 5’-TCTCACAATCTCCCTTCCATTCC-3’  5’-AGGTGAACCAAGATGTTATTCTCTG-3’ |
| *CsFAB2.3* | CsFAB2.3-F  CsFAB2.3-R | 5’-ATGCAAACACTTACCTCAAACCAC-3’  5’-AGCGGCAGCACATTGTCATTG-3’ |
| *CsFAD2.1* | CsFAD2.1-F  CsFAD2.1-R | 5’-TCTCAAGAAGACAGATTCTGACCAC-3’  5’-AGCCCAGGCTGGATAAAATAAGAG-3’ |
| *CsFAD2.2* | CsFAD2.2-F  CsFAD2.2-R | 5’-ATGAGGAAAGGAAGCCCAAATAGA-3’  5’-GTAGTGAAGCAGTAGTGGGTG-3’ |
| *CsFAD3* | CsFAD3-F  CsFAD3-R | 5’-AGAATAAGCCACAGAACTCATCATC-3’  5’-TCACCTGGAGCAAATAAATCACTG-3’ |
| *CsFAD4* | CsFAD4-F  CsFAD4-R | 5’-ATGTCCATCTTAGCTCAAAACAAGTTC-3’  5’-TGTAAGTGGAGTAGGAGATATAACC-3’ |
| *CsFAD5.1* | CsFAD5.1-F  CsFAD5.1-R | 5’-ATGGCTCTTCTCAATTCTAAATTCCAC-3’  5’-ACTCTGGCTCAAATGCTTCTGTC-3’ |
| *CsFAD6* | CsFAD6-F  CsFAD6-R | 5’-AGCTATGGATTTGAGCAAATTGGAG-3’  5’-ATGCCCAAGCCAAAGGTAGTAG-3’ |
| *CsFAD7* | CsFAD7-F  CsFAD7-R | 5’-AGTGTTGTTGGCCATATCCTTCAT-3’  5’-AGAGCGAACCTTAGTGTTCTTGTT-3’ |
| *CsAct1* | CsAct1-F  CsAct1-R | 5’-TGGATTCTGGTGATGGTGTGAGT-3’  5’-CTGCTCATAGTCGAGTGCAACATA-3’ |
