# Supplementary material for: Characterization of the Fatty Acid Desaturase Genes in Cucumber: Structure, Phylogeny, and Expression Patterns
Source: PLoS One. 2016 Mar 3;11(3):e0149917. doi: 10.1371/journal.pone.0149917 (PMC4777478; doi:10.1371/journal.pone.0149917)
Supplement: S1 Fig — The intron sequences are shown in lowercase letters. (PDF) [file pone.0149917.s003.pdf]

Supporting Information

>CsFAB2.1

```
1      AAAAAGCCCATTTTATAGAGAGAGAAGGAAAAAGAGAAAAAGAAAGAGAAAGAGAGGAATTGAGGTCGAAAGTGGCCATTGCCGGCAGGT
91     AGTCTTAGCATCACATTCATTCACATACGTAAAGTGGCCTCGGTTTCTTCAACTCCGGCTCCGGTATTCTTCTTCCTCTTTCCTTCACGT
181    ACTGTTTTTCCGACTAACTCACCTGAATATTCAACGTTGATTACGGAGCTACTCTGAAAATGGCGCTCAAATTCCATCCTCTCACCTCTC
1                                                                  M  A  L  K  F  H  P  L  T  S
271    AATCTCCTAAGTTGCCGTCCTTTGCGATGCCGCAGCTTGCAAGTCTCAGATCTCCTAAGTTTGTTATGGCCTCCACTCTCCGCTCCACCT
11     Q  S  P  K  L  P  S  F  A  M  P  Q  L  A  S  L  R  S  P  K  F  V  M  A  S  T  L  R  S  T
361    CCAGgtaacttcctttttctttttcctcattcttcttcttttttttttaatgcttttcagatctttgtgaatcttttggttatgtgttccct
41     S  R
451    tgttttgcattgcttatcattctgttaacttctgttttcgttctcttgtatatcgttgtttccgttcatttcgaggactgatgttttgtt
541    gctaatgaatttattcgatttatcattacctcggattgacatttagttgcatggttggcgcgttttgttctgttgattttctgcttccat
631    gtgttgctcctctgcgtgattctccgcgcattttatcttcttgatgcggttccttccatgaatttgattttcagttagttttgtggttcc
721    attggaatttcatgatttttcttcatgtcgtcatatttttgacgattgcgtctgatctctttttttcaaatggagtttgcattgaatgg
811    tgatgagcatggaggtttttcagttgtattatttttgtttctgtctccgtcttcgttttctattacatatcacctcgactgagtgattat
901    ggatcattcctgctagaacgaacaggcttattccgttttagctcttcgaaatgaaattagatgttcaattttatttttctttgacagtga
991    attcgaactgtagttaattgtgtggttgcatgactgtccattcggtattctttggtgtattaggcagcgttgtgttcagtgttctgtctt
1081   gttttggaggcttcttggtgcaggtctgtccttggaatttttcggtcttaacttgattgcttttctttattatgtggcatcatcatcggc
1171   tcttgatgtcccagctcatttatgaggattctgatcttccgctgctcatttattccatatcctgtgctgtctctgcttatatgtttaaaa
1261   cctttaagagaccaaatttcttttgggtgtaaatctttatttaatccaatctttttacttgtcacgactttcaaaacaaccattcgtttg
1351   aagtacctgaaggattaaacatttagctacctggtaaaaatctgacatattggaaacatatacttaatgtctttatgacgtatgatgtgg
1441   ccattttgttgtgcagctctcttctcgatccatgaggtcttttgcacagaatgcctctggacagcattttaattctgagacttaccattt
1531   gcattgagaaaacgcttaattgaatagaagttctagaaagctaccatataatctgcctattcagtgatgtgatatctctgttcattaact
1621   tttttaactgtgtgttttgtaaaattccagGGAGGTTGAGACGCTTAAGAAACCCTTTATGCCTCCAAGGGAAGTGCATCTTCAAGTAAC
43                                    E  V  E  T  L  K  K  P  F  M  P  P  R  E  V  H  L  Q  V  T
1711   TCACTCAATGCCACCCCAAAAGATGGAGATATTTAAATCTTTGGAGGACTGGGCTGAAGAAAACCTTTTGGTTCATTTGAAACCAGTGGA
63      H  S  M  P  P  Q  K  M  E  I  F  K  S  L  E  D  W  A  E  E  N  L  L  V  H  L  K  P  V  E
1801   GAGGTGTTGGCAACCACAGGACTTTCTGCCAGACTCGGCATTTGAGGGATTTCACGAGCAGGTCGTGGAGCTTAGAGAGAGGGCAAAGGA
93      R  C  W  Q  P  Q  D  F  L  P  D  S  A  F  E  G  F  H  E  Q  V  V  E  L  R  E  R  A  K  E
1891   ACTCCCTGATGAGTACTTTGTTGTTTTAGTTGGAGACATGATTACAGAAGAAGCCCTTCCTACTTACCAGACAATGCTGAATACTTTGGA
123     L  P  D  E  Y  F  V  V  L  V  G  D  M  I  T  E  E  A  L  P  T  Y  Q  T  M  L  N  T  L  D
1981   TGGAGTCAGGGACGAAACAGGAGCAAGCCCCACCCCTTGGGCAATTTGGACAAGGGCTTGGACTGCTGAAGAGAACAGACATGGTGACCT
153     G  V  R  D  E  T  G  A  S  P  T  P  W  A  I  W  T  R  A  W  T  A  E  E  N  R  H  G  D  L
2071   TCTGAATAAATATCTTTACCTATCTGGACGAGTGGACATGAGGCAGGTTGAGAAAACAATTCAGTATTTGATTGGATCGGGAATGgtgag
183     L  N  K  Y  L  Y  L  S  G  R  V  D  M  R  Q  V  E  K  T  I  Q  Y  L  I  G  S  G  M
2161   atccgtctaaaccatcaaatttttcttttttccttatagctatatttcgatttaaaaaattaccttgaaccccataaatacactatgcacaa
2251   aaaggctgatgagtgcacgattgtccacactcccttgagtatttaattccttccattctctgctgtttctgcagcatgagtcttgcatgc
2341   attctgtctgataactgataaaccagtttaaattatattgagtcaagtttgttcttcataaagattttttttggcagaattctctcttta
2431   gctggacatcagaattcccatatgttcaacaacttatgtatgttgtaaaattagttggtatattaaagtaattatcattggagatgctat
2521   gagtatttatgaacctttttagcctttaggttgatctttaggtggtaggctcttcaatatattttaatcccttctcatttttataattctt
2611   atggattgtgttatagGACCCAAGGACTGAAAACAATCCTTACCTAGGATTTATATACACCTCATTTCAAGAAAGGGCAACCTTCATCTC
211                      D  P  R  T  E  N  N  P  Y  L  G  F  I  Y  T  S  F  Q  E  R  A  T  F  I  S
2701   CCATGGGAACACTGCCAGACTAGCAAAAGAGCACGGAGACATAAAGTTAGCTCAAATATGTGGAACAATCGCTGCAGATGAGAAGCGACA
241     H  G  N  T  A  R  L  A  K  E  H  G  D  I  K  L  A  Q  I  C  G  T  I  A  A  D  E  K  R  H
2791   CGAGACTGCATATACAAAAATTGTCGAAAAGCTCTTTGAGATAGACCCTGAAGGAACGGTCATAGCTTTTGAAGACATGATGAGGAAGAA
271     E  T  A  Y  T  K  I  V  E  K  L  F  E  I  D  P  E  G  T  V  I  A  F  E  D  M  M  R  K  K
2881   AGTCTCAATGCCTGCTCATCTGATGTACGATGGCCGCGACGACAACTTGTTCCATCACTTCTCAGCAGTTGCACAGAGGCTTGGAGTTTA
301     V  S  M  P  A  H  L  M  Y  D  G  R  D  D  N  L  F  H  H  F  S  A  V  A  Q  R  L  G  V  Y
2971   CACAGCAAAAGACTACGCAGACATATTGGAGTTTTTAGTTGGGAGATGGAAGGTGGAAAGTTTGACTGGACTGTCAGGCGAGGGGCAGAA
331     T  A  K  D  Y  A  D  I  L  E  F  L  V  G  R  W  K  V  E  S  L  T  G  L  S  G  E  G  Q  K
3061   GGCTCAGGATTATGTGTGCGCATTGCCTGCAAGAATTAGAAAGCTAGAAGAAAGAGCTCAGGGACGGGCAAAAGAAGGTCCTACCATTCC
361     A  Q  D  Y  V  C  A  L  P  A  R  I  R  K  L  E  E  R  A  Q  G  R  A  K  E  G  P  T  I  P
3151   TTTCAGTTGGATTTTCGATCGACAGGTTAAGCTGTAGAAGGTTGAATGGAGTAGGAAGGAAGAGAATGTGTAGAAGAGGAGTTGATGGGG
391     F  S  W  I  F  D  R  Q  V  K  L  *
3241   TAAGGGAAAAAGAAAAAAAAAAAAAGCAGAAATCATCATTTGGTGTACTTTGTCTTTTTAATGTGTTGAAGAACAATAATGGTCAGTATCA
3331   TCTTCTTTGCTCTGTTTTGTCCATTCCTTCTCTCTTTCTCTCTCTCTCTCATCAAAAAAAAAAAAGGGAAAAAAAATTGTGTGTATTGCAG
3421   TGTAGATACAGTATTTTATGCGTCGATCTGTTTGGTTTAGGTGTGATAGATGGTAGGAAGGTAATGGCAGTGTTGTTTGTGTGTGTTGTG
3511   TGCTTTAGTTTTTGTCACTTTTACTTCATAATGTAAAATCTGTTTCCTTGGTGTATCAGAACAATCTACAATTTTGATCATCAATTCTTT
3601   CCATCTCTCGCTCCAAAGACACGTTTTTCCTCGTTTTAAAAAAGAGAAGTTTTAGAAATCC
```

>CsFAB2.2

```
1     TTCCCCTCTTTCTTTCTTCTTTATATTTCCCTCTCTCTCGCCGATCCCCCAACTCTCTTCCTCCATAATGGCTCTCAAGCTCAATTCCCC
1                                                                        M  A  L  K  L  N  S  P 
91    ACTGAAACTCCCATCTCACAATCTCCCTTCCATTCCCCTTCCCCATCGCAGATCTCCAACTCTCCTCATGGCCTCCTCCACTCTCCACTC
9      L  K  L  P  S  H  N  L  P  S  I  P  L  P  H  R  R  S  P  T  L  L  M  A  S  S  T  L  H  S 
181   TCTCTCCAAgtaagtatctaaccttcccattttcctctctcctctgttccatcaatttcttttattctactgcctattcatcccacttcc
39     L  S  K 
271   cttttacttttctttttactgtattctgattttattcgttcaatcttcaccgattctaatctgaaattgcttatttctcttcatattgcg
361   ttccttggaatctccgcctacctcaatcaatcaagctatatagtgagacgatttttgtattaataatgtaatttttgtctgaagaaagag
451   gcatttttcagagattgtttttgtacttgcaagaaatctccagaaggatggatttttttttcacagaatttctaatttagttatttttt
541   taattgccaatactacaacgtttattaaatgattttgcaatagcatcgattctttttccctttttatgcgattctttccaaacggtatct
631   atcagtcttttgttgtttttttctccctctctttttttagttatttgtttattgttaggtcatgttgatagcagagtcgtgtttgttat
721   attatataaatattgttgtatgtatataaagttaaattatatcttcaattacgtttgcatttgtgaatttagaattttcaattctgttta
811   aatcttggccccttcagaaaaatttaattaaaagttacattttgctcattccatgacgatgtggaattgtggagggatgtgaccatcaat
901   aatttctattaagaaaatttgatttattattttcttaaacatatgttttctttgaaaattacgcagccacatactttcactcttgagta
991   atatgcttttattcttttctcgattctctcattttatatcgtttgagtttttagccaaatttttggaaatacaacagttttttgtgaga
1081  ttgctttttttaagttcttagaagcttgctattactttttaaatcgtttctggaatgaagaactaaaacattaagaaaatataacttcgt
1171  ttttcagccaaaaattgagaaccatttaagagtgctacctaataaaatacaattacatttaaaaagttgtacaaaactaattaaatgtaa
1261  tcacgattcacaactaaatatgtagttagcttcttgttttcctgttgtttcttttctatggtcgtctaatgtttgtgtttatggctgaag
1351  tcatgtcctatagactatagttgctttctactaactaccgtctattggcttcttcatactacatttccattcaatttggtatctagcagt
1441  gtgcaatcgcccattcaggtttagcttgtattttcttgagtggaagtgcttgtaaaactgtattctgagactctcgtatgtgactgagtt
1531  ttgatggccatcttcttttctaactattcttaataattgttaatcttgtaaattaaactgaatcatacatctgagactgacatcactgtt
1621  gcttattctatttacagATCGACTGAGGGATCAAAGAAGCCAAATTTCCCTCCCCGGGAAGTGCATGTCCAAGTGACTCATTCCATGCC
42                       S  T  E  G  S  K  K  P  N  F  P  P  R  E  V  H  V  Q  V  T  H  S  M  P 
1711  ACCTCAAAAGATGGAGATCTTCAAATCGTTAGAAGACTGGGCAGAGAATAACATCTTGGTTCACCTAAAGCCTGTTGAAAGTTGTTGGCA
66     P  Q  K  M  E  I  F  K  S  L  E  D  W  A  E  N  N  I  L  V  H  L  K  P  V  E  S  C  W  Q 
1801  ACCTCAGGATTATCTCCCAGACCCAGAATCTGACGGTTTCTACGACCAAGTCAAGGAAATAAGGGAGAGATCGAAGGATATTCCTGATGA
96     P  Q  D  Y  L  P  D  P  E  S  D  G  F  Y  D  Q  V  K  E  I  R  E  R  S  K  D  I  P  D  D 
1891  CTATTTTGTGATGTTGGTTGGTGACATGATTACCGAAGAAGCGCTTCCAACTTATCAGACAATGCTGAACACCCTCGATGGTGTTCGTGA
126    Y  F  V  M  L  V  G  D  M  I  T  E  E  A  L  P  T  Y  Q  T  M  L  N  T  L  D  G  V  R  D 
1981  TGAGACGGGTGCAAGCCTCACCTCGTGGGCAATTTGGACAAGAGCATGGACTGCGGAAGAGAATAGACATGGTGACCTTTTGAATAAGTA
156    E  T  G  A  S  L  T  S  W  A  I  W  T  R  A  W  T  A  E  E  N  R  H  G  D  L  L  N  K  Y 
2071  CCTTTACCTGTCAGGACGAGTCGATATGAAGCAGATTGAAAAGACCATCCAGTACCTTATTGGATCAGGAATGgttaggtttctcgcttt
186    L  Y  L  S  G  R  V  D  M  K  Q  I  E  K  T  I  Q  Y  L  I  G  S  G  M 
2161  ctcgaaaataactttttgtttcggtttctttgaacaatttactgtgacctggaaaagtagagatttgtgtgcacttgaactcacaattcaa
2251  ctcataagtactataagtcatgcgagaaaatttacttgcaaaaactaagaaaatattaactttatgaacatcaactaagcatgtttccat
2341  tgaagttgtgtctttaattgtagagaacacttttcaaaaacactctcaaatagcacctatgtcttttacaaatgtgtggtttgttagaat
2431  tctgagttcaaaccattagaactttgttagtttgttagggatttaattgggtacttcgaactcttttagtggttttacagttaacagtat
2521  cggatttgatggtaaatcatagtgagtagtgaaacttgctgcttcaacataatgttctttgctgcttgtctgagtgttagaatagtccat
2611  tgttacattttcagcttgtatgtttttttgtgtactttggattgctacatttagtatctgtctgtgaccatagtttattataagatttta
2701  tggtgtttctctttttagattcaaattgaacccatggtttgtttttgtgccattttagGATCCCAAAACCGAAAACAATCCATACCTTGG
210                                                             D  P  K  T  E  N  N  P  Y  L  G 
2791  TTTCATCTACACCTCATTTCAAGAGAGGGCAACTTTCATCTCCCATGGAAACACTGCTAGGCTGGCCAAGGAACATGGTGATTTTAAGTT
221    F  I  Y  T  S  F  Q  E  R  A  T  F  I  S  H  G  N  T  A  R  L  A  K  E  H  G  D  F  K  L 
2881  GGCACAGATATGTGGTATAATTGCATCTGATGAAAAACGTCATGAAACCGCATATACCAAGATTGTTGAGAAGCTCTTTGAGGTTGACCC
251    A  Q  I  C  G  I  I  A  S  D  E  K  R  H  E  T  A  Y  T  K  I  V  E  K  L  F  E  V  D  P 
2971  TGATGGCACAGTTATGGCTTTAGCTGACATGATGAAGAAAAAAATTTCCATGCCAGCCCATTTGATGTACGATGGACAGGATAACAACCT
281    D  G  T  V  M  A  L  A  D  M  M  K  K  K  I  S  M  P  A  H  L  M  Y  D  G  Q  D  N  N  L 
3061  TTTTGAGCACTTCTCATCTGTTGCTCAGCGGCTCGGAGTTTATACTGCTAAAGACTATGCTGACATTCTGGAGTTCCTCGTTAGCAGATG
311    F  E  H  F  S  S  V  A  Q  R  L  G  V  Y  T  A  K  D  Y  A  D  I  L  E  F  L  V  S  R  W 
3151  GAAAATAGAGAATTTGACAGGTCTCTCGGGTGAGGGACGCAAAGCACAAGATTTCGTGTGCGGTTTGCCGCCAAGGATTAGAAGGCTGGA
341    K  I  E  N  L  T  G  L  S  G  E  G  R  K  A  Q  D  F  V  C  G  L  P  P  R  I  R  R  L  E 
3241  AGAGAGGGCTCAAAGCAGAGCAAAACAAGCACAAAGAGTCCCATTTAGCTGGATCTTCGGTCGAGAAATACAAGTTTAAGGGCAACGGTC
371    E  R  A  Q  S  R  A  K  Q  A  Q  R  V  P  F  S  W  I  F  G  R  E  I  Q  V  * 
3331  TTAAAGGAAGGTGTTAAACTACTTGACTCAACTTGACAGAGGCCTTTCAGACGACATTCGAATACAAAATCGGAAGCGTTTTCATCTTTA
3421  GGTATCACTTCCAAATCCTAATTGTTGAAGGGAAAAGTATGTAGATCAGACTGTCTTGTTGCTGCTGCTGTATTTGTTGATGCAACAACT
3511  ATGTCACTTCCAGCTCCTTATTTCTTTAGAAGAAAAGGATCAATATTGGCTTGAGTTTTGTTTTTCTCGTACCCAAATAGAACGCCTAGT
3601  AAGTTTTCAACTGTTGTAATCACCAAACCATCTATAAATTTTGGCAAAAAAAAATCCTAGTTGCACTTTTGCTTTATTTATCTGTCC
```

>CsFAB2.3

```
1     TTTATAAATATATTAGGTCATTTCCCTCTTCCAATTGCCCCAGAATTTCTACTTCCACGAAATAATACAACACACCATCCTCATCATGCA
1                                                                                             M  Q
91    AACACTTACCTCAAACCACCTTCCCCTTACCCGGAATATCCCTCACTTCCGCCTCAACTCCCCCCCGCCACTCTACCGCCTCAAATCTCC
3       T  L  T  S  N  H  L  P  L  T  R  N  I  P  H  F  R  L  N  S  P  P  P  L  Y  R  L  K  S  P
181   AATTTCAGCCATTGCGGCCCCGCCTCTGGTTAAGCATCGGAGAATGCACTCAATGCCCCCCGAAAAGATCGATGTCTTCAAGTCCTTGGA
33      I  S  A  I  A  A  P  P  L  V  K  H  R  R  M  H  S  M  P  P  E  K  I  D  V  F  K  S  L  E
271   AGGTTGGGCCAATGACAATGTGCTGCCGCTGCTGAAGCCGGTGGATGACTGCTGGCAGCCCCAGGATTTCTTGCCTGATTCTACTCTGCC
63      G  W  A  N  D  N  V  L  P  L  L  K  P  V  D  D  C  W  Q  P  Q  D  F  L  P  D  S  T  L  P
361   CTACGGGGAATTTGTGGAGGAAGTCAGGGCTCTGCGGGATCGGACGGCGGAGCTGCCGGATGAGTACTTTGTGGTGTTGGTTGGGGATAT
93      Y  G  E  F  V  E  E  V  R  A  L  R  D  R  T  A  E  L  P  D  E  Y  F  V  V  L  V  G  D  M
451   GATTACGGAGGATGCATTGCCCACTTATCAGACCATGATTAATACGCTGGATGGAGTTAAGGATGAGACTGGGGCTAGCCAGAGCCCCTG
123     I  T  E  D  A  L  P  T  Y  Q  T  M  I  N  T  L  D  G  V  K  D  E  T  G  A  S  Q  S  P  W
541   GGCTACCTGGACCCGGAATTGGACCGCAGAGGAGAACCGGCACGGCGACTTGCTCCGGACATACCTTTATTTATCCGGTCGAGTTGATAT
153     A  T  W  T  R  N  W  T  A  E  E  N  R  H  G  D  L  L  R  T  Y  L  Y  L  S  G  R  V  D  M
631   GACGATGATTGAACGGACTGTTCAGTATTTGATCGGCGCCGGCATGgtgagaccaattcccctttaattatttctaatttaatttaacga
183     T  M  I  E  R  T  V  Q  Y  L  I  G  A  G  M
721   aaattatttgatttttcaacacttttgaaaattacattttattttgtcaaacacagaattcaatttgatttatacagattcactcgttaa
811   tttttttttttaaaaaaatgattgaatttgataaagttgatgatctacttatttactaatgagagttctttttttagaaaaagaaattag
901   aacaaacttgtaattaaagtgcatttataacatgtaattattagttcattttaagtcgggttgtttttttttttaatattttattaatgt
991   ttacttttaattacatttaggatgcgatgcttttttccaaatttaatagttgaatagttaggaaggtgttaattaattaaatttgtttt
1081  ggaagcaaagaagtaaacaaggaggagatgaaaaacgaatttgaatacaccaatcctgggaataagttttttatttaaaaaagaagaaac
1171  gattgaatgaataaaattatatggtaacaatggagatggaaatgtgaatacgtgggaggtgggcagGATCCAGGAACGGAGAATAACCCA
198                                                                         D  P  G  T  E  N  N  P
1261  TACCTAGGATTCGTTTACACCTCATTTCAAGAGCGAGCCACGTTCATTTCTCACGGCAACACCGCTCGCTTAGCCAAACACAACGGCGAT
206    Y  L  G  F  V  Y  T  S  F  Q  E  R  A  T  F  I  S  H  G  N  T  A  R  L  A  K  H  N  G  D
1351  CCAGTCCTTGCCCGCATCTGCGGCACCATCGCCTCCGACGAGAAGCGCCACGAGAACGCATATTCAAGAATCGTCCAGAAGCTTCTAGAG
236    P  V  L  A  R  I  C  G  T  I  A  S  D  E  K  R  H  E  N  A  Y  S  R  I  V  Q  K  L  L  E
1441  GTGGACCCCACAGGAGCCATGCTGGCAATAGCTGACATGATGCAGAAGAAGATCACCATGCCGGCTCACCTTATGTACGACGGGCAGGAC
266    V  D  P  T  G  A  M  L  A  I  A  D  M  M  Q  K  K  I  T  M  P  A  H  L  M  Y  D  G  Q  D
1531  CCGAGGCTATTCGACCACTTCTCTGCCGTGGCTCAGCGCCTTGGAATCTACACGGCTAATGACTACGCGGACATTCTGGAGGTACTGATC
296    P  R  L  F  D  H  F  S  A  V  A  Q  R  L  G  I  Y  T  A  N  D  Y  A  D  I  L  E  V  L  I
1621  CAACGGTGGGGGTTGGAGAAGGTGGAGGATGGATTGACGGGGGAGGGGCGACGGGCTCAGGATTACGTGTGTGGGTTGGCGCCCAGGATT
326    Q  R  W  G  L  E  K  V  E  D  G  L  T  G  E  G  R  R  A  Q  D  Y  V  C  G  L  A  P  R  I
1711  AGGAAGCTTCAGGAGCGAGCCGACGAGCGGGCTAAGAAGTTGAAGCCCCATGGGGTCAAGTTTAGCTGGATTTTTAATAAGGAAATTCCC
356    R  K  L  Q  E  R  A  D  E  R  A  K  K  L  K  P  H  G  V  K  F  S  W  I  F  N  K  E  I  P
1801  ATTCTCTCCTAGGTGGTTCTTCACTTGTTAGCTTTGTTTTTTGTTTTTGTTTTTCTTTTCCTTTCCTGTAAATTTAAATTTAGATTTAAA
386    I  L  S  *
1891  TTTAAAATCTTAAACCCAAAAACTTCAAAAAATAAAAATAATTGAAATCTTGTTTAATTTA
```

>CsFAD2.1

```
1     CCTCCAAATAAAGGAAAAATATTCACACAAGAGAGAGAGAGAGAGAGAGAGAGAAAAATAAGGCTTTGTTGCCGGCGATCTGAAGGCGGC
91    GGCCATTGCTCTCGAGAGAGAGAGAAAGAGAGAGATTGAAAGAGCGGAGAGTACGAGGCTCTCTCAAACTTCGGTCCTCTTCTTCTCTTT
181   CAGgtatccttctctatcccttcctattctgtttccgcttttctctttcttcgccatcatgctcgttctcttcttttgttctctcactca
271   atgtgattgactttatgttgtttttctgttttattttcccattaacgtttgttgtaatgtgtagatctatgataagatttgaattagtgc
361   tcattaatgtgttgcatgcttttgatttcgttttaagcagagattactttgtctatattcattaaatcattggattttaggttctttcag
451   agtttgtaaacagtgatgttaaggaatgctgagatttatgactgatgagagttcgtgtttgtctattagcttgttggtttcctctttgat
541   atcgcatggattcgatctgtatgtctgggtttgactcttgctgaaatggcttcactatagcatatttgagtttgtgttgttgaagatttg
631   ttgatttcttggaaaattgggagttcgttttgtttttcctctttttacaggtttattgattggcgatatctcgttttcaacttccgaaat
721   gctatttttcataagaagaaactgtggatgtatttttctactcgattagagatccttgaaactatgccaaaaggaattgtttttttcacc
811   aaattgtttttttgtcgtttgtgatattaatgcattttcttattcttaataaagtttaagtgttcctttattatttttttaatggtggttgt
901   tggaatggttatttcccttttactaaaagctttttccatgtgattcaaaggtgtatttggggtttcccggtctttgttccaagtcaatta
991   ggatgggcgccaattcggttttagcttctgtatcattggtgtatattttgttatagggaggaaaagaaggaagaagaaaaatcttccgtc
1081  ctacagtgtgctgagtaaactcgagcagccttttctgctgaatactttttaaaattatttttttaactgtgatttggtgaacttaaattgt
1171  tttaataaataatgtggattaaatcttaacagaaacgatttaaaaaaaaaacatatttttagtgaaatctttattttatttaaaagatct
1261  ccatcagtcctgatgtttcctagaaaacttaaaaaataatgggtagctagaaatatggaaataaatgtatttgagcttctccttgaaac
1351  tggagcagtggtcaatttttttttcgtttgtgtagatgtgatagaaatagaatattccgttcccttccggcatcatcagagaggtggaatt
1441  ggtctttctcaacctcagtatcaattaaattaagtttcgtgataaatttcaaatgtttggtagggtcaaatagtttgtaaaatccctagc
1531  cgtccattatgatgcaaacggaaggatttcacttgctcttttaaattactaaaaatatttcgtcgttgatgttgcctgtctgttttatca
1621  tttctctttccgcctcaagaggcggctaagtgtcttggcaagttaattttgttttgtacttccgccccttgtcttttggcccttttaatt
1711  aagtttttccctttaatctcctggtcggcgtacattgaattattaggtttgcatttaatttctatgtttataaatctgttaaagcaatta
1801  atgtggtactgtttgatctagatattttgaagtctaaaacgttaaaccctttgtttgatttgtttttttttttttttgttccagGTCAAT
1891  ATCCTTGAAAAAATGGGTGCTGGAGGTCGAATGTCCTCTCCTTCATCTCTCAAGAAGACAGATTCTGACCACTTGAAGCGAGTACCGTCC
1                   M  G  A  G  G  R  M  S  S  P  S  S  L  K  K  T  D  S  D  H  L  K  R  V  P  S
1981  ACAAAACCCCCATTCACACTTGGTGAGCTCAAGAAAGCTATTCCACCTCATTGCTTTCAACGTTCAGTCCTTCGTTCTTTCTCCTATGTC
27     T  K  P  P  F  T  L  G  E  L  K  K  A  I  P  P  H  C  F  Q  R  S  V  L  R  S  F  S  Y  V
2071  GTTTATGACCTAACCTTAGCCTCTATCTTCTATTACATTGCAACCACATATTTCCAAAATCTTCCTAGCCTCTTATTTTATCCAGCCTGG
57     V  Y  D  L  T  L  A  S  I  F  Y  Y  I  A  T  T  Y  F  Q  N  L  P  S  L  L  F  Y  P  A  W
2161  GCTTTGTACTGGGCTGCCCAAGGCTGCGTCCTTACTGGCGTTTGGGTCATTGCCCACGAGTGTGGCCACCACGCCTTCAGTGATTACCAG
87     A  L  Y  W  A  A  Q  G  C  V  L  T  G  V  W  V  I  A  H  E  C  G  H  H  A  F  S  D  Y  Q
2251  TGGCTGGATGACACCGTTGGCCTTATCCTCCACTCGTTTCTCATGGTCCCCTACTTCTCTTGGAAATACAGCCACCGTCGACACCATTCC
117    W  L  D  D  T  V  G  L  I  L  H  S  F  L  M  V  P  Y  F  S  W  K  Y  S  H  R  R  H  H  S
2341  AACACCGGATCTCTTGAACGAGATGAAGTCTTTGTCCCTAAACAGAAATCCGACATGAAATGGTACTCCACATACATCAACAACCCTGTA
147    N  T  G  S  L  E  R  D  E  V  F  V  P  K  Q  K  S  D  M  K  W  Y  S  T  Y  I  N  N  P  V
2431  GGCCGTGTCCTCACCCTCACCATCCAACTGGTCCTAGGCTGGCCTCTTTACTTGGCCTTCAACGTCTCTGGCCGTCCCTACGACCGTTTT
177    G  R  V  L  T  L  T  I  Q  L  V  L  G  W  P  L  Y  L  A  F  N  V  S  G  R  P  Y  D  R  F
2521  GCCTGCCACTATGACCCATACGGTCCCATCTACACTGACCGTGAGCGGTTACAGATATTCATTTCGGACGCTGGGCTGCTAACTGTCACC
207    A  C  H  Y  D  P  Y  G  P  I  Y  T  D  R  E  R  L  Q  I  F  I  S  D  A  G  L  L  T  V  T
2611  TATGGGCTCTACCGCCTTGTTCTAGCCAAAGGATTGGCTTGGGTGGTTTGTGTTTACGGAGTTCCACTTCTGATCGTAAATGGATTCCTC
237    Y  G  L  Y  R  L  V  L  A  K  G  L  A  W  V  V  C  V  Y  G  V  P  L  L  I  V  N  G  F  L
2701  GTCCTAATCACATACCTTCAACACACTCACACAGCACTGCCACATTACGATTCCTCCGAATGGGACTGGCTAAGAGGTGCCTTGGCAACC
267    V  L  I  T  Y  L  Q  H  T  H  T  A  L  P  H  Y  D  S  S  E  W  D  W  L  R  G  A  L  A  T
2791  GTAGACAGAGACTACGGCATCTTGAACAAGGTCTTCCACAACATTACGGACACTCATGTGGCTCATCATTTGGTGTCCACAATGCCACAT
297    V  D  R  D  Y  G  I  L  N  K  V  F  H  N  I  T  D  T  H  V  A  H  H  L  V  S  T  M  P  H
2881  TACCATGCCATGGAAGCAACCAAGGCAATCAAGCCAATTTTGGGCCAATACTATCACTTTGATGGGACCCCGGTTGTGAAGGCAATGTGG
327    Y  H  A  M  E  A  T  K  A  I  K  P  I  L  G  Q  Y  Y  H  F  D  G  T  P  V  V  K  A  M  W
2971  AGAGAAGCAAAAGAGTGCATTTACGTGGAGCCTGATGAAGGTGAGAAGAAAGGCGTTTATTGGTATAAAAACAAGTTGTAATTTGCTTGT
357    R  E  A  K  E  C  I  Y  V  E  P  D  E  G  E  K  K  G  V  Y  W  Y  K  N  K  L  *
3061  GGAAGAAGCAGTGTGAGAGAGAAAAGTGCCTTTGTCTACTTAAAAGTGGGGTAAACTTTTAAGCATCTTGGTTCTGCTACTACTACTACT
3151  CTAGGAGAGCTGTTTGTTTTTCTTCTTTGTTATATGAACCTTAGTTTCTGTTATGGGTAAGTTTAATTCCTACCAAAATGCCAACTCTTT
3241  TGTTAAGTCAGTCAAGTTTCTTGGTTGGTTTTACTTTGTTGGTTCGGGTCTAGATTACAAGATACTCGTAAGGTATGCAGATGCTTAATG
3331  ATGATTAAATGTTAATGCATGCGAATTGACTTTTCTTCAGATTGCGTCTATGTTATGGATAGATTAGGATGTCCATGTGTAATATATTTT
3421  CATGTATGGAAAAACATTGCTAGACTTGGCCTCACCGTTCTTTATCCATTTATTGATTAGATGACATATCATTTATCATTTTAAATGTGG
3511  AATGGAAACATGAGGTTGGAAAAGGATAATAAAATAAGTGAACCATTGTTTTTCCTT
```

>CsFAD2.2

```
1      ATGAGGAAAGGAAGCCCAAATAGAAGCAAATTAGCATTGGGGGAGCGCATTACACACACCAAGCCTCCCTTCACCATCACACAAATCAAG
1       M  R  K  G  S  P  N  R  S  K  L  A  L  G  E  R  I  T  H  T  K  P  P  F  T  I  T  Q  I  K
91     AAATCCATTCCATCCCATTGCTTCAATCGTTCCCTCTACCGCTCCTTCTCTTACGTTATCTTCGACTCCATCCTTCTCTCTACTTTTTAT
31      K  S  I  P  S  H  C  F  N  R  S  L  Y  R  S  F  S  Y  V  I  F  D  S  I  L  L  S  T  F  Y
181    TACGTAGCCACCACCTATTTCCACACCCTTTCCGCCCACCCACTACTGCTTCACTACTTAGCTTGGCCTCTTTATTGGCTCTCTCAAGGC
61      Y  V  A  T  T  Y  F  H  T  L  S  A  H  P  L  L  L  H  Y  L  A  W  P  L  Y  W  L  S  Q  G
271    ATTGTCTTCACCGGCTTCTGGGTCATCGCTCATGAATGTGGCCATCATGCCTTCAGTGATTACCAATTCCTAGATGACTTCCTTGGCTTC
91      I  V  F  T  G  F  W  V  I  A  H  E  C  G  H  H  A  F  S  D  Y  Q  F  L  D  D  F  L  G  F
361    CTCCTCCATTCTTCCCTCCTCATCCCTTACTTCTCTTTCAAAATCAGCCACCGCCGCCACCATGCCAACACTGCATCACTCCACCGCGAC
121     L  L  H  S  S  L  L  I  P  Y  F  S  F  K  I  S  H  R  R  H  H  A  N  T  A  S  L  H  R  D
451    GAGGTCTTTGTCCCCAAGCCCAAATCCAAAATCCCTTGgtatggtgttaattaggtttaatatcattattaaataccatgattggatgtt
151     E  V  F  V  P  K  P  K  S  K  I  P  W
541    tatatgaggcacaaacatatagaaactgatgtgagaaatagatgataccttattttctaaaaacagatatgaatatatatatttttatta
631    aaaaattcaagatatgagaaaccctaatgcatttcttcacaatctctctatcttctctatgaatcactctctaaaactattggttttcat
721    ggatcaaagtatgctattctaactcatgttattgtgaaaattatctagttcaaaatgttgtctcttattattttatataatgaagctttc
811    tatggtgtataatcatgaagttaaaatttacagGTATTTCAAACACTTAACCAACCCACCTGCTAGACTCTTCATTATTGTAATGACCCT
164                                      Y  F  K  H  L  T  N  P  P  A  R  L  F  I  I  V  M  T  L
901    CACACTTGGCTGGCCAATGTACATAGCCTTCAACAATTCAGGCCGAGTCTATGATAGATTCACCAGCCACTACGATCCAAACAGCCCAAT
183      T  L  G  W  P  M  Y  I  A  F  N  N  S  G  R  V  Y  D  R  F  T  S  H  Y  D  P  N  S  P  I
991    ATTCACCGAAAAGGAACGACTTCAAGTTCAAATATCCAATGCCGGAGTCTTAACCATTTTATACTTACTTTACAAACTCGCAGTTACAAA
213      F  T  E  K  E  R  L  Q  V  Q  I  S  N  A  G  V  L  T  I  L  Y  L  L  Y  K  L  A  V  T  K
1081   AGGAATCACGTGGGTCATTCGCATATACTTACTGCCTTTAACGGTTATGAACGTGTTTGTGGTGTTGATATCATGTTTACAACACACACA
243      G  I  T  W  V  I  R  I  Y  L  L  P  L  T  V  M  N  V  F  V  V  L  I  S  C  L  Q  H  T  H
1171   CTCTTCATTGCCGTATTACGATTCTAGTCAGTGGGATTGGCTGAGAGGGAATTTGGTTACAGTGGATAGAGATTACGGGAAGATTTTGAA
273      S  S  L  P  Y  Y  D  S  S  Q  W  D  W  L  R  G  N  L  V  T  V  D  R  D  Y  G  K  I  L  N
1261   TAAAATGTTACATAATATAACGGATACACATGTGATTCATCACTTGTTCCCTTCAATGCCACATTACAATGCAGCGGAGGCGACGAGGGC
303      K  M  L  H  N  I  T  D  T  H  V  I  H  H  L  F  P  S  M  P  H  Y  N  A  A  E  A  T  R  A
1351   GGTGAAGGAAGTGCTGGGAGAGTACTATCAGTTTGATGAGACGCCTATCTTGAAGGCTGCATGGAGGGAGTTTAGAGAATGTGTTTATGT
333      V  K  E  V  L  G  E  Y  Y  Q  F  D  E  T  P  I  L  K  A  A  W  R  E  F  R  E  C  V  Y  V
1441   AGAGGAAGATGATCATGAGGAGGGGGACTCAGAAGCTTTTAGAAGTAAAGGGGTATTTTGGTTTCGGAACAAGCTTTGA
363      E  E  D  D  H  E  E  G  D  S  E  A  F  R  S  K  G  V  F  W  F  R  N  K  L  *
```

>CsFAD3

```
1     AGAGATGGGTGAGTGGCCTAGTCTTTCGTAAGCAAAGAGACCAATTAGCATTGCCCCTTTTTATAATCTCCATCTTCCCCCATCTTTCAT
91    TTGCCCTTTTTCTCTCTTCCATTTTTCTCTCAACCACAACAGCTGATACAACAAAAAAGGCCATTTCCCATTTCCCCTAATTCTCTCTCG
181   AAAGGGAAATATGGCCATTCCTCTGCTGGCCAACGCTAACGCCACTGCCGCCGCTCCGGCCCCTTTCGACCCCTCCGCCCCTCCTCCTTT
1                M  A  I  P  L  L  A  N  A  N  A  T  A  A  A  P  A  P  F  D  P  S  A  P  P  P  F
271   CCGTATATCCGAGATCCGTGCCGCCATTCCTCCCCATTGCTGGGTCAAGAGCCCTTGGCGCTCTCTTCTTTACGTTCTTCGAGACTTAGC
28     R  I  S  E  I  R  A  A  I  P  P  H  C  W  V  K  S  P  W  R  S  L  L  Y  V  L  R  D  L  A
361   CATCGTCTCCGCATTGGTCGCCGCCGCGCTCTTCTTCGACTCCTGGCTCGTTTGGCCCATCTATTGGCTCGCTCAGGGCACCATGTTTTG
58     I  V  S  A  L  V  A  A  A  L  F  F  D  S  W  L  V  W  P  I  Y  W  L  A  Q  G  T  M  F  W
451   GGCCATTTTCGTCCTCGGTCATGACTGgttcgaatctcatcttcctctgtttttttttttttttttttttacactctgttctctttttc
88     A  I  F  V  L  G  H  D  C
541   tttctgtttacgatctctctgttttctttttccagTGGCCATGGAAGCTTCTCCAACAGTTCTGCGCTTAATTCCTTCATGGGTCTTCTT
97                                         G  H  G  S  F  S  N  S  S  A  L  N  S  F  M  G  L  L
631   CTTCATTCCTTCATTTTGGTTCCTTATCATGGATGgtaccatttctccctccttcccgaatcaaaatccccagatcgatcttcctctatt
115    L  H  S  F  I  L  V  P  Y  H  G  W
721   tttctgttcataatttgtttttttttggaaatctgaatctgaaaccatcattggatttttctgaaacagGAGAATAAGCCACAGAACTC
127                                                                          R  I  S  H  R  T
811   ATCATCAAAACCATGGAAATGTGGAGAAAGACGAATCCTGGGTTCCAgtacgttcgtcagatttgtcagatctctctgttttttttaaaaa
133   H  H  Q  N  H  G  N  V  E  K  D  E  S  W  V  P
901   aaatttctttctcctctgtttagcttttacattcatttcaatgactgttattttgtatcagTTGACAAAGAAGACTTACAAACAACTAGA
149                                                             L  T  K  K  T  Y  K  Q  L  E
991   GAAAAGAACAAGAATCCTCAGATTCACTTTACCTTTCCCCATTCTCGCATACCCATTTTACCTCgtaattatctatctctcatttcggtt
159    K  R  T  R  I  L  R  F  T  L  P  F  P  I  L  A  Y  P  F  Y  L
1081  tttcgtttttttttttctcgatttgtttcgaaacagtaattgtatattggtaaatattttgcagATGTGGAGAAGCCCAGGGAAAGAAGGA
180                                                                    M  W  R  S  P  G  K  E  G
1171  TCTCATTTCAATCCATACAGTGATTTATTTGCTCCAGGTGAAAGAAGAGACATTGTGATTTCAACCTCATGTTGGACATTAATGGCGGCT
189    S  H  F  N  P  Y  S  D  L  F  A  P  G  E  R  R  D  I  V  I  S  T  S  C  W  T  L  M  A  A
1261  CTTTTAGTTTATTTATCCTTCGTTTTTGGTCCATTCCAAATCTTCAAACTCTACGGCGTTCCTTACTGGgtaattactttttctataaaa
219    L  L  V  Y  L  S  F  V  F  G  P  F  Q  I  F  K  L  Y  G  V  P  Y  W
1351  aaaaataattgtgtagccgaaatgaaagacctttctacttgcttcctccattaaaattggtccatttctcaattcttttaaggaatgaat
1441  taatgagatctaatgacggtcaacatctttgcattttcaacattaaaagaatttccttttctatttgttcctctcacatggcacaactca
1531  tcaatatttaattattaaaaatgtttccaactttttaatttccccttcatgggtttttgtttttggatctgaatgcaaattgggcttttt
1621  tttttctttttttcttttttttttttgaagATATTTGTCATGTGGCTGGACGTTGTGACTTATTTGCACCACCATGGGTATGAACAGAAGC
242                                  I  F  V  M  W  L  D  V  V  T  Y  L  H  H  H  G  Y  E  Q  K
1711  TGCCATGGTACAGAGGAGAGgtaaagaaagaaagaaaaaaagaaatagaatttccctttcccacttattttcttgggaaacgaaactttt
262   L  P  W  Y  R  G  E
1801  aaaaacccaagtggggatttcttttgattttttttaataattattttctaatttctgatggacagGAATGGACTTACCTACGTGGTGGAC
269                                                                      E  W  T  Y  L  R  G  G
1891  TGACAACCGTTGATCGAGATTATGGATTGTTCAATAACATCCATCATGATATTGGAACTCATGTCATCCACCATTTATTTCCTCAAATCC
277    L  T  T  V  D  R  D  Y  G  L  F  N  N  I  H  H  D  I  G  T  H  V  I  H  H  L  F  P  Q  I
1981  CTCACTATCATCTTGTTGAAGCGgtatgtcctttttacccctttatattaccactacacaactgagatttcatctccatctttcaaatca
307    P  H  Y  H  L  V  E  A
2071  ttattaatgtttaaacatctataaataatacaacgagaagttcgtagttcgtagttaaaaagaaatgttttaatgttctaaacaatgttt
2161  aaacaatgcttaaacaatacttacatttcttttcaaagaagtcaataatatggctttttaaagcaccaggacaaaccaaaccattacac
2251  tctaaaaaagtgttgaaattatgaacaacaacttggaaaggaacaattattttaaaatctatcttaataggatagcttctgttctattta
2341  gcgtttgatttgacaacgaaataacatttttgttcttagatgtggtaggtggttggatgatagataccgatgtgattgtctttgtttcttt
2431  acatttcataatgtttttttcttaagtctaaatttccaattaatttctaacggtataattaggtcaataagcatcaatttattttaccttt
2521  taaggcagagacctttttgaaacaaagttttagaaattctccttaataaaaaagtaaattagggtagtgggtaattgtttttttgtttttt
2611  gttttcctttaaaagaatagctaaaaactacaaggaaaacaagatagattgtaagagaagagaagagatttagaaagtcaaacaataata
2701  attaatttgatttgcaatgaattgtttttggcagACAAAGGCAGCAAAGGGAGTATTGGGGAAGTATTATAGAGAGCCGAAGAAATCAGG
315                                        T  K  A  A  K  G  V  L  G  K  Y  Y  R  E  P  K  K  S  G
2791  GCCAATTCCAACTCATTTGGTGAAGAATTTGGTGAATAGCCTTAAACAAGACCACTATGTGAGTGACCAGGGCAATATTGTCTTTTACCA
334     P  I  P  T  H  L  V  K  N  L  V  N  S  L  K  Q  D  H  Y  V  S  D  Q  G  N  I  V  F  Y  Q
2881  GACAGATCCTTATCTTTACCACTACTAAAATTTAGAAAGAAAAAAAAAAAAAATTGATCATTGGAAAAAGAGAGAAAAAAAAAAAGAGAAA
364     T  D  P  Y  L  Y  H  Y  *
2971  AAAATCGATTCTGTTTAGTTGAGTTTCTCATCCCTTTCCTTTAGAATTGTTCTTTCTTGTTGCCTTTAGTTTGCACTTTGTATTATTACA
3061  TTTCCCAAACTACCCCTCTCCCCCCCCTAAGAGAACAAAAAAAAGAAGAACATTTTTCCTTTTTGATCCCATCCTCTCCAAACAAAAACA
3151  AATAAATTTAATTCAATTCTCCTCTATTTACATCTTTATTTTTTGTTATTGTTTTTTCTATTATTACTTTTGTTTTTCTAGTTGATGGGG
3241  TTTACATCATTTTAGTTCATCTTCTTCCATAAGCAATTCTCTAAATAGAGAATTAAAATTTAAG
```

>CsFAD4

```
1     CGTCCAAAAAGAAAAAGGAAGAAGTTTGTAGCAAAATCTCAGCCTCTGCGTCTCTATCTTCAATCTTGACTCTTCACCCAACCAAACAAT
91    TGGTTTTACAAATTTGTGGTACCAAAAACTCACAACTTTAATTATGCTTCGAAGGCAGAAACAGTTCCACGTAGTCACATAAATTTTCCA
181   AAATGAAACAAAAGTTTTCCTTCAGAGAATCCCTTTAGTATAAACCTCAATATTAACATAATCCTACTCAGCTTCTGTTCCCACCACACC
271   ATTGTCTTTTCTTCCTTCTTCTTCCTCTCTTTCCTCTCCACAGATTCTTGGCCTCTATTTCAACTCACTCTCTTCAATATTTTGCCACCC
361   ATCATTCCTCTATTGGTTTCTTCTTCTTCTTCCTTTCCAATGTCCATCTTAGCTCAAAACAAGTTCCTTCTGAGCTCTCGGTCCCATGTT
1                                                 M  S  I  L  A  Q  N  K  F  L  L  S  S  R  S  H  V
451   AATCCATGCAGCACTCCGATTCCACGCAGAGTTGTCTACTGCATAGCTAATACCACCGTCAATGGTGTTGCTACCAGTCCGAAGCATGCT
18     N  P  C  S  T  P  I  P  R  R  V  V  Y  C  I  A  N  T  T  V  N  G  V  A  T  S  P  K  H  A
541   GATGGTCAACTAGTCATTGAGCGTGAGGTTATATCTCCTACTCCACTTACATTTGTCAATTCCTCCACCTATTATTCTGTTCTTAATGAC
48     D  G  Q  L  V  I  E  R  E  V  I  S  P  T  P  L  T  F  V  N  S  S  T  Y  Y  S  V  L  N  D
631   CCGAGTTTACGATCAACTTGGAGTCATCGTGCGTGGGTGGCTGGTGGGTGCACCACAGTGCTAATCTCTTTGGCAAAATCAATAGTGGGT
78     P  S  L  R  S  T  W  S  H  R  A  W  V  A  G  G  C  T  T  V  L  I  S  L  A  K  S  I  V  G
721   GCAGCCAGTTCACATATCTGGCTTGAGCCTGCTTTAGCAGCATTGGTAGGCTACATTTTAGCTGACCTTGGATCCGGAGTATATCATTGG
108    A  A  S  S  H  I  W  L  E  P  A  L  A  A  L  V  G  Y  I  L  A  D  L  G  S  G  V  Y  H  W
811   GGCATCGATAACTATGGTGATGCATCAACCCCAATCTTCGGTACTCAAATAGAAGCATTCCAAGGCCATCACAAGTGGCCCTGGACAATC
138    G  I  D  N  Y  G  D  A  S  T  P  I  F  G  T  Q  I  E  A  F  Q  G  H  H  K  W  P  W  T  I
901   ACGAAGCGACAGTTCGCCAACAATTTGTACGCTTTGGCACGAGCTGTGACTTTTGCTGTACTTCCTGTAAGCCTTGCAATTGATGATCCC
168    T  K  R  Q  F  A  N  N  L  Y  A  L  A  R  A  V  T  F  A  V  L  P  V  S  L  A  I  D  D  P
991   ATTCTACATGGTTTTGTTGGTATGTGCTCAGGTTGCATAATGTTTAGCCAACAGTTTCATGCATGGGCTCACAGTAAAAAGAGTGAACTA
198    I  L  H  G  F  V  G  M  C  S  G  C  I  M  F  S  Q  Q  F  H  A  W  A  H  S  K  K  S  E  L
1081  CCTCCACTCGTGGTGGCAATGCAAAATGCTGGCCTGCTCGTGTCACGAACACAGCATTCAACACATCATCGTCCACCCTATAACAACAAC
228    P  P  L  V  V  A  M  Q  N  A  G  L  L  V  S  R  T  Q  H  S  T  H  H  R  P  P  Y  N  N  N
1171  TATTGCATAGTAAGTGGAATATGGAATAAGCTTTTGGATGAAACAAAGCTTTTTGAGGCATTGGAGATGATCTTGTACTTCAAACTTGGG
258    Y  C  I  V  S  G  I  W  N  K  L  L  D  E  T  K  L  F  E  A  L  E  M  I  L  Y  F  K  L  G
1261  CTGAGACCAAGATCTTGGACTGAGCCTAATTCTGAATGGACTGAAGAGACCGAGATTCTTGAACAAACTACAGCCCATTAACAACATTCT
288    L  R  P  R  S  W  T  E  P  N  S  E  W  T  E  E  T  E  I  L  E  Q  T  T  A  H  *
1351  TCTTTCATTCATATTGTGCTTTTCCTTGCAAGAATAGAAGGAAACAGAATTTATTTGAAATATTTTCATACGTAAACTATATATGATG
```

>CsFAD5.1

```
1     AAAACAAACACAAACCACAACTCTGTTTCAACACTAAATCACCTCATAAATTCATTCACAAACCGACAATTTTCCCCCTTCCATTTATAA
91    ATCCCCACAGCACAATCACTTCAATTCTTCACAGTCGCCTGCTTTTTTCTTTTCCCCCGATGGCTCTTCTCAATTCTAAATTCCACCGCT
1                                                               M  A  L  L  N  S  K  F  H  R
181   TCCCATCTCTCCGCCCTTCAATCCACCGTTCACCCCTCCCCACCCTTAATTTTCCCTCCATTCCCCATGACCAATTACCCTTTCATTTTG
11    F  P  S  L  R  P  S  I  H  R  S  P  L  P  T  L  N  F  P  S  I  P  H  D  Q  L  P  F  H  F
271   TGGGTCGTCCTAATTTCAATTCCCTGAGGTTGAATTCCCCAATTCATATCCAAAACAGAGCAATTTCTTCGCCGACAGAAGCATTTGAGC
41    V  G  R  P  N  F  N  S  L  R  L  N  S  P  I  H  I  Q  N  R  A  I  S  S  P  T  E  A  F  E
361   CAGAGTCGTTGCCGCCGCCACCGGCGTCGGCGGTGGATGGGAGGAGGATTTTGTTGTCGGACGTAGTGGTGAAGCGGCGGAGGGAAGTGT
71    P  E  S  L  P  P  P  P  A  S  A  V  D  G  R  R  I  L  L  S  D  V  V  V  K  R  R  R  E  V
451   TTTGGGGAAGGAAATGGAATTCGTTAGATATCGGAACGGCTGGAGTGGTGGTGGCGACGCATTTACTTACACTTTTGCTCCTTTTCAAT
101   F  W  G  R  K  W  N  S  L  D  I  G  T  A  G  V  V  V  A  T  H  L  L  T  L  F  A  P  F  Q
541   TCAATTGGGCTGCATTTTGGGTGGCGATTTCTTTATATATAATCACTGGTCTATTCGGTATTACCCTATCATTTCATCGGAATCTCTCTC
131   F  N  W  A  A  F  W  V  A  I  S  L  Y  I  I  T  G  L  F  G  I  T  L  S  F  H  R  N  L  S
631   ATCGGAGCTTTAAACTTCCTAAATGGCTCGAATATCTCTTCGCATATTGTGGAGTTCAAGCCCTTCAGgttcttaaagaagaaagaaaaa
161   H  R  S  F  K  L  P  K  W  L  E  Y  L  F  A  Y  C  G  V  Q  A  L  Q
721   gaatcccctgtttttcccatgttgtttaggctttgtttaatgtatgctctgtttgtttcttacactgattaggttattttcaacgtcgtt
811   gcagGGAAATCCGATCGATTGGGTTAGCACGCATAGATATCACCATCAATTTTGCGATTCCGAAAGAGACCCGCATAGTCCAATTGAAGG
184       G  N  P  I  D  W  V  S  T  H  R  Y  H  H  Q  F  C  D  S  E  R  D  P  H  S  P  I  E  G
901   CTTTTGGTTTAGTCATATGAGTTGGTTGTTTGATACCAATTCTGTGGTTGAGAGGgtgagaattcaattcaatggcttgaattttaccca
213    F  W  F  S  H  M  S  W  L  F  D  T  N  S  V  V  E  R
991   aaaaagttaatgttttaaattctaatttggatattgtttttgggttagTGTGGAGAGCCAAACAATGTTGGGGATTTGGAAAAGCAACC
231                                                 C  G  E  P  N  N  V  G  D  L  E  K  Q  P
1081  ATTTTACAAGTTCATTCAAACTACTTATCTTCTTCATCCAATTGCTCTTGGTGCTTTACTCTATTCAATGGGTGGATTTCCTTTCATTGT
245    F  Y  K  F  I  Q  T  T  Y  L  L  H  P  I  A  L  G  A  L  L  Y  S  M  G  G  F  P  F  I  V
1171  TTGGGGAATGgtaagtttggattacttatgtcccttttgatgttattgattttgggttaattccacctttcttaaatttatgtgtttctt
275    W  G  M
1261  aaaggatttaggccaagtagtcacaatataggagtaagtaaatatacaattgatttgacacttcgaatgaaagagacaattaatataag
1351  ctaagtttaaacattcttaattaattcaattgtctttttcaatgagaaaaagaaaaagaaagaaagagttgacttgtttttgaaagtggg
1441  ctatggattgggcttacccatatgggcttttgtgaatctccatggtccattgaaagattagtagagcatgagacctaaaaaaaatattag
1531  cagggcagatatagaaacagtaacgagacctgtagtattatcagatatgttatatagcaaactttagactcggtaatagactatacatgt
1621  tggcagtatatataggttaaattgtgattaaccaattatagaacttgtgttgtgattcttaattacagGGTGTGAGAATAGTGTGGGTGT
278                                                                       G  V  R  I  V  W  V
1711  ACCATATCACTTGGCTGGTGAATTCAGCTTGCCATGTTTGGGGAAGTCAAGCTTGGAAAACAGGAGATTTATCTAGGAACAACTGgtaac
285   Y  H  I  T  W  L  V  N  S  A  C  H  V  W  G  S  Q  A  W  K  T  G  D  L  S  R  N  N  W
1801  ttttaacttcccatactccccttttatatgacaatgataagcattgattaatatgtttattggcgaagGTGGGTGGCATTGCTTGCATTT
314                                                                         W  V  A  L  L  A  F
1891  GGAGAGGGATGGCACAACAACCACCATGCATTCGAGTTCTCAGCCCGACATGGCCTAGAGTGGTGGCAGCTCGACATGACATGGTACGTT
321    G  E  G  W  H  N  N  H  H  A  F  E  F  S  A  R  H  G  L  E  W  W  Q  L  D  M  T  W  Y  V
1981  GTTCGGCTTCTTCAAGCTATTGGTTTGGCCACAGATGTGAAGATACCAACACAACTTCAGAAAGAAAAATTGGCAATCATCAACACCACA
351    V  R  L  L  Q  A  I  G  L  A  T  D  V  K  I  P  T  Q  L  Q  K  E  K  L  A  I  I  N  T  T
2071  TGATATAAAGATATTATACTAAAACTTTAATTTTTTGTAAGCAAATCTTCAGTACCAAAAACTGTATGTCTTTCTCGTTTCGAGATTCAA
381    *
2161  ATGGGTTCTGAAGGCTAAAGTATATAGAAAGAGTAGATAGCATTTCAATCGGGAGACATTGGAATTGTTCTCTCTGCACTCAACTTTACA
2251  TAGCATATGATGTATTCACTAATTTAATTAGAGAGTTTTTTTCTCCCCATTTTTTATGATTTTTGTTGCAATACAGTTCGGTTTCGTTTT
2341  GAACAACGAAAACTTGTAAGTTATACGTTTTAAAATGGATTGGAAAAGAAGATTACAACATGATTTTA
```

>CsFAD5.2

```
1      ATGGATACAATGGTGAAGAAGGCTGATCTAATCACATTTGGGCAGAGAAAATGGACAAACAGAGACAAATATATGGCAGCTTTTTTTATT
1       M  D  T  M  V  K  K  A  D  L  I  T  F  G  Q  R  K  W  T  N  R  D  K  Y  M  A  A  F  F  I
91     TTCCTGCACTTCATTTGTATTCTTGCACCCTTCCATTTCAATTGGAATGCATTTTGGGTTGCGTTTGCTTTATACATTATTACAGGTTGT
31      F  L  H  F  I  C  I  L  A  P  F  H  F  N  W  N  A  F  W  V  A  F  A  L  Y  I  I  T  G  C
181    TTTGGAATCACTATTTCGTATCATAGAAATCTTTCGCATAGAAGTTTCAGACTTCCCAAATGGCTCGAATACTTTTTTGCATATTGTGGA
61      F  G  I  T  I  S  Y  H  R  N  L  S  H  R  S  F  R  L  P  K  W  L  E  Y  F  F  A  Y  C  G
271    GCCCTTGCTTTTCAGgtttgacattttaaaactttcttcttttttttattttaaacttaaaaaatttttactagaaagaggttcttatcat
91      A  L  A  F  Q
361    ttgacattaagaaaaatgaaaaatgttgagacacatttctatcgtccctactttcctaagtagttttgatcttatttgtttttctatttt
451    aactttagGGTGATCCAATCGATTGGGTGAGTACACACAGATGTCATCATCAATTTGTCGATACGGAAAATGACCCGCATAGTCCTATTC
96                G  D  P  I  D  W  V  S  T  H  R  C  H  H  Q  F  V  D  T  E  N  D  P  H  S  P  I
541    AAGGATTTTGGTTTAGTTATTTCATTTGGATTTTGGATTCGAATGCTTTGACCAGAAGAgtttgtccacagtatttcattgatcataaag
123    Q  G  F  W  F  S  Y  F  I  W  I  L  D  S  N  A  L  T  R  R
631    atacagaaaaaaccatcttcacagtggttttaaagTATGGAAGACCAAATAATGTTGGTGACTTGGAAAAACAATTCTTCTATAGGTTTC
143                                        Y  G  R  P  N  N  V  G  D  L  E  K  Q  F  F  Y  R  F
721    TTCGCAAAACTTATTTTCTTCATCAACTTGCTCTTGCAATCCTTCTTTATGCAGTCGGAGGAACACCTTTTCTTATATGGGGAGCGgtaa
161    L  R  K  T  Y  F  L  H  Q  L  A  L  A  I  L  L  Y  A  V  G  G  T  P  F  L  I  W  G  A
811    aataaaattactttattattattatttttttaaaaaaattaaccaaaatattaacaaatataaataaatatcattctttatcgatgatga
901    gtaatgatatacatagatagactacaatcatctatcattgataatagacactgatagacatctatcaatatatatcatttatactatatt
991    tatatatattaattacaacacagttttgtcgtttaaaatacatttataaattttatatatttgaatacatttttacgttgacttataaat
1081   gaagtggacatgatactttatattattattaataaatctctttgttttgcagTTTGTGAGGACCATAGGAACCATACATGTGACGTTTAT
190                                                          F  V  R  T  I  G  T  I  H  V  T  F  M
1171   GGTGAATTCAATATGTCATACATTTGGAAATCAACCATGGAATACTGGAGATTTGTCGAGAAATACTTGgtataagtgcttttgtatata
203      V  N  S  I  C  H  T  F  G  N  Q  P  W  N  T  G  D  L  S  R  N  T  W
1261   ttttctttttcatttaatctcggttgtcaaatagttgtgtcgacctttatttagttagcttggtaatattctttaaatatggtaaattt
1351   acatgtcaaccaaaattaagagaagcattttatcaaaagtagtttcttaaaagaagtataaatataatcaatttagaaaaatgaacgcca
1441   acaaaaatgctattagttaacgtgtgtatataattagttcaatacaatatggtgcctaaaaaattcattagtcaacaaacacaaaaaaat
1531   gcccgtctatgatctataatatcaatgatttaatctttttaaaaaaattatcaatcaaattagcatatattagaattgtaaattggtatc
1621   ctaactcttttagaatttggaaatgtttcatatatgtctaattgtagtttaaaaagaaactgaaactagcatatattaactagtttatgt
1711   catgaacgattcataattaattatcaaagttagtaacccataaaagtttaccgttcgaagaaaactacctcaaaagtttaagagttggga
1801   ggagagcatacaaatatgagagttccgtaactaaacttgcaaaagtagttagagtgctggttaaagctattccctcgtatgtgggttgtt
1891   gttcgtctagtctggtacggttcaagtaggtttaggcaattatatgggtgcaaaaatgagagaaactttttgtttatttttatcatttctc
1981   ttcttaaattggatctgaaatttcaattatgtaataaactaaacctaaacattttattattcttcaataaccatattctatagaaactct
2071   attgtttaccttttattgattcttccatcttccgtttttcttttttcttttcgtttttttttttaatgatggtgagcttcctcttgattccaac
2161   tccctttccctctccctctttaacgaatgaacgttcaaggtgggtattgtgttgactttggagaataattgacataaactatttccacca
2251   aatttgctttcaagaaaataattttcacgacacaaaacattacactcatattagactagacaacaaattagcaaatatatatatatatag
2341   tgaaatgaaatgaacgattattgaatgttgtctttgtaaagtaaacaaagctaattccacattatagctaatgcctttgtatgttgcaaa
2431   ttaattaataaaatatttatggttttgtttttttagGTGGATGTGTTTGATTTCATTCGGAGAAGGTTGGCATAATAACCATCATGCTTT
226                                         W  M  C  L  I  S  F  G  E  G  W  H  N  N  H  H  A  F
2521   TGAGTATTCAGCCAGACAAGGGCTTGAATGGTGGCAGATCGATATTTCTTGGTACATTATTTGGTTTCTTCAGGTCATTGGATTAGCAAC
244      E  Y  S  A  R  Q  G  L  E  W  W  Q  I  D  I  S  W  Y  I  I  W  F  L  Q  V  I  G  L  A  T
2611   TGAGGTTAAAGTACCATCTCAATCTCACAAGCAAAGACTACAAGCTTTGGACCAAACCAAAAGAAAAGGGCTTTGA
274      E  V  K  V  P  S  Q  S  H  K  Q  R  L  Q  A  L  D  Q  T  K  R  K  G  L  *
```

>CsFAD5.3

```
1     ATGGATACAATGGTGAAGGCTGATCAAATCCCATTTGGGGAGAGAAAATGGACAAACAGAGACAAATATATGGCAGCTCTTTTTGTTATT
1      M  D  T  M  V  K  A  D  Q  I  P  F  G  E  R  K  W  T  N  R  D  K  Y  M  A  A  L  F  V  I
91    ATGCACTTCATTTGTATTCTTGCACCCTTCCATTTCAATTGGAATGCATTTTGGGTCGCTACTCTGTTATACTTCTTCAGTTTTTTTGGA
31     M  H  F  I  C  I  L  A  P  F  H  F  N  W  N  A  F  W  V  A  T  L  L  Y  F  F  S  F  F  G
181   ATCAATATTTCGTATCATAGAAATCTTTCACATAGAAGTTTCAGACTCTCCAAATGGCTCGAATACTTTTTTGCATATTGTGGAGCTCTC
61     I  N  I  S  Y  H  R  N  L  S  H  R  S  F  R  L  S  K  W  L  E  Y  F  F  A  Y  C  G  A  L
271   GCTTTTCAGgtttgatattcttaatactttcttctttctttcttatttgacattaaaaaaatattgagagacgttctaatgtatctactt
91     A  F  Q
361   ttgtaagtagtttcaatgttatattctaaaagaaactaaaacatattatacttactgtttttttttttttaaattttagGGTGATCCAATC
94                                                                                  G  D  P  I
451   GATTGGGTGAGTACACATCGATGTCATCATCAATTTGCTGATACAAAAAATGATCCACATAGTCCTATTCAAGGATTTTGGTTTAGTTAT
98     D  W  V  S  T  H  R  C  H  H  Q  F  A  D  T  K  N  D  P  H  S  P  I  Q  G  F  W  F  S  Y
541   TTCACTTGGCTTTTGGATTCCAATGCTTTGACTAAAAGAgtttgtccacagtattttattgatcataaagatacagataaaaccatcttc
128    F  T  W  L  L  D  S  N  A  L  T  K  R
631   acattggttttaaagTATGGAAGACCAAATAATGTTGGCGACTTGGAGAAACAGTCCTTCTATAGGTTTCTTCGCAAAACTTATTTTCTT
141                   Y  G  R  P  N  N  V  G  D  L  E  K  Q  S  F  Y  R  F  L  R  K  T  Y  F  L
721   CATCAACTTGCTCTTGCAATCCTTCTTTATGCGGTTGGAGGAACACCTTTTCTTGTATGGGGAATGgtaaaataaaattacttatttatt
166    H  Q  L  A  L  A  I  L  L  Y  A  V  G  G  T  P  F  L  V  W  G  M
811   tattaacaaaattaatcaaaatatttatagatatataataaaatactattgttatcgacaatgaaatagttatatacacatataaactac
901   aattatcatctaccactaataatagacgacagcgatagacatcaatcaatgttactcatttataaacaatgacataattttattatattt
991   ctatatatttacaacgttattatcgcttgaaacaagtatttttatatttgaatatatatttttatttgaacttataaatgaagcaagcat
1081  atgatactttatattattataaataagattcattgttttgcagTTTGTGAGGACCATAGCATTCCTACATGTGACCTTTATGTTGAATTC
188                                                F  V  R  T  I  A  F  L  H  V  T  F  M  L  N  S
1171  AATATGTCATACATTTGGAAATCAACCATGGAACACTGGAGATTTGTCTAAAAATACTTGgtataagtgcttttgtatatatatattttt
204     I  C  H  T  F  G  N  Q  P  W  N  T  G  D  L  S  K  N  T  W
1261  tcattttatcttcatcttcatatagttgtgacctttattttagttagctattaatattctttaaatacggtaaatttaagtggttaatat
1351  atgccaaccaaattaagaaaaacattttaaaaagtagtttcttaaacaaagtataaatataagtcatttataaaataaacggctactaat
1441  catattagttaacgtgtgaaaaattcattagtcaacaaataatacaataaatgcgaggctttgataatatcaatgattaattttaaaaaa
1531  aattatcaatgattaatctttaaaaaaattatcaatgatatcttccaatgtcataactgtagttagtggtagaaaaattgattatccaac
1621  tcttttaaaattacaaataatctttcatatatctctaattgtagtttaaaaggaaaaagaaatcagcatatattaactagtttatgtctt
1711  gaatggttcatagttacgtaacccataacagttcacacttcgaaaaaagccacctaaaaggtttaagggttgggaggagagcataaaaat
1801  atgagagttcttggactaacttgcaatagtaaaggatcttccctcataggtaggctgttgcttcgttaaaatattcaggtacttgtactg
1891  aggcgaaaatgagagaaacacgtatttttgtttattttattctttttctttctttaaatgttatgaaattaaacttggatctgtaattt
1981  caattatataataactaaatctaaacattttattattttattgattctaattccatcttcctaatcattctttttttctttttttaataa
2071  agaagagcttttcctctatttcaattccttctccgacgaatgcacgttcgaagtgggtattgtgttaactttagagagcaattgacataa
2161  atgattcccaccaaatttgctttcaagataataatttccacaacacgaagctttacatccatattagaccagatgacatatatagcatat
2251  atatcattagtgaattggaatagacaatcgttgaatgttgtgtttctaaagtaaacaaagctaactccacattatagctaaagcctttct
2341  atgttgcaaattaattaattaacacaatatttttttgtttgtttttagGTGGATGTGTTTGCTTACACTTGGAGAAAGTTGGCATAATAAC
224                                                     W  M  C  L  L  T  L  G  E  S  W  H  N  N
2431  CATCATGCATTCGAGTATTCAGCCAGACAAGGACTTGAATGGTGGCAGATCGACATTTGTTGGTACATTATTTGGTTTCTTCAAGTCATT
238    H  H  A  F  E  Y  S  A  R  Q  G  L  E  W  W  Q  I  D  I  C  W  Y  I  I  W  F  L  Q  V  I
2521  GGATTAGCAACTGAGGTTAAAGTACCCTCTCAATCTCACAAGCAAAGACTACAAGCTTTGGACCAAACCAAAAGAAAAGAACTTTGA
268    G  L  A  T  E  V  K  V  P  S  Q  S  H  K  Q  R  L  Q  A  L  D  Q  T  K  R  K  E  L  *
```

>CsFAD5.4

```
1     TTCCTTTTCTTTCCAAAACAAACACAAACCACAACTCTGTTTCAACACTAAATCACCTCATAAATTCATTCACAAACCGACAATTTTCCC
91    CCTTCCATTTATAAATCCCCACAGCACAATCACTTCAATTCTTCACAGGATTTTGTTGTCGGAAAAAGGABPATGAAGCGGCGGAGGGAA
1                                                                                M  K  R  R  R  E
181   GTGTTTTGGGGAAGGAAATGGAATTCGTTAGATATCGGAACGGCTGGAGTGGTGGTGGCGACGCATTTACTTACACTTTTTGCTCCTTTT
7      V  F  W  G  R  K  W  N  S  L  D  I  G  T  A  G  V  V  V  A  T  H  L  L  T  L  F  A  P  F
271   CAATTCAATTGGGCTGCATTTTGGGTGGCGATTTCTTTATATATAATCACTGGTCTATTCGGTATTACCCTATCATTTCATCGGAATCTC
37     Q  F  N  W  A  A  F  W  V  A  I  S  L  Y  I  I  T  G  L  F  G  I  T  L  S  F  H  R  N  L
361   TCTCATCGGAGCTTTAAACTTCCTAAATGGCTCGAATATCTCTTCGCATATTGTGGAGTTCAAGCCCTTCAGgttcttaaagaagaaaga
67     S  H  R  S  F  K  L  P  K  W  L  E  Y  L  F  A  Y  C  G  V  Q  A  L  Q
451   aaaagaatcccctgtttttcccatgttgtttaggctttgtttaatgtatgctctgtttgtttcttacactgattaggttattttcaacgt
541   cgttgcagGGAAATCCGATCGATTGGGTTAGCACGCATAGATATCACCATCAATTTTGCGATTCCGAAAGAGACCCGCATAGTCCAATTG
91              G  N  P  I  D  W  V  S  T  H  R  Y  H  H  Q  F  C  D  S  E  R  D  P  H  S  P  I
631   AAGGCTTTTGGTTTAGTCATATGAGTTGGTTGTTTGATACCAATTCTGTGGTTGAGAGGgtgagaattcaattcaatggcttgaatttta
118   E  G  F  W  F  S  H  M  S  W  L  F  D  T  N  S  V  V  E  R
721   cccaaaaaagttaatgttttaaattctaatttggatattgtttttgggttagTGTGGAGAGCCAAACAATGTTGGGGATTTGGAAAAGC
138                                                       C  G  E  P  N  N  V  G  D  L  E  K
811   AACCATTTTACAAGTTCATTCAAACTACTTATCTTCTTCATCCAATTGCTCTTGGTGCTTTACTCTATTCAATGGGTGGATTTCCTTTCA
150   Q  P  F  Y  K  F  I  Q  T  T  Y  L  L  H  P  I  A  L  G  A  L  L  Y  S  M  G  G  F  P  F
901   TTGTTTGGGGAATGgtaagtttggattacttatgtccctttttgatgttattgattttgggttaattccacctttcttaaatttatgtgtt
180   I  V  W  G  M
991   tcttaaaggatttaggccaagtagtcacaatataggagtaagtaaatatacaattgatttgacacttcgaatgaaagagacaattaata
1081  taagctaagtttaaacattcttaattaattcaattgtctttttcaatgagaaaaagaaaaagaaagaaagagttgacttgtttttgaaag
1171  tgggctatggattgggcttacccatatgggcttttgtgaatctccatggtccattgaaagattagtagagcatgagacctaaaaaaaata
1261  ttagcagggcagatatagaaacagtaacgagacctgtagtattatcagatatgttatatagcaaactttagactcggtaatagactatac
1351  atgttggcagtatatataggttaaattgtgattaaccaattatagaacttgtgttgtgattcttaattacagGGTGTGAGAATAGTGTGG
185                                                                              G  V  R  I  V  W
1441  GTGTACCATATCACTTGGCTGGTGAATTCAGCTTGCCATGTTTGGGGAAGTCAAGCTTGGAAAACAGGAGATTTATCTAGGAACAACTGg
191    V  Y  H  I  T  W  L  V  N  S  A  C  H  V  W  G  S  Q  A  W  K  T  G  D  L  S  R  N  N  W
1531  taacttttaacttcccatactccccttttatatgacaatgataagcattgattaatatgtttattggcgaagGTGGGTGGCATTGCTTGC
221                                                                               W  V  A  L  L  A
1621  ATTTGGAGAGGGATGGCACAACAACCACCATGCATTCGAGTTCTCAGCCCGACATGGCCTAGAGTGGTGGCAGCTCGACATGACATGGTA
227     F  G  E  G  W  H  N  N  H  H  A  F  E  F  S  A  R  H  G  L  E  W  W  Q  L  D  M  T  W  Y
1711  CGTTGTTCGGCTTCTTCAAGCTATTGGTTTGGCCACAGATGTGAAGATACCAACACAACTTCAGAAAGAAAAATTGGCAATCATCAACAC
257     V  V  R  L  L  Q  A  I  G  L  A  T  D  V  K  I  P  T  Q  L  Q  K  E  K  L  A  I  I  N  T
1801  CACATGATATAAAGATATTATACTAAAACTTTAATTTTTTGTAAGCAAATCTTCAGTACCAAAAACTGTATGTCTTTCTCGTTTCGAGAT
287     T  *
1891  TCAAATGGGTTCTGAAGGCTAAAGTATATAGAAAGAGTAGATAGCATTTCAATCGGGAGACATTGGAATTGTTCTCTCTGCACTCAACTT
1981  TACATAGCATATGATGTATTCACTAATTTAATTAGAGAGTTTTTTTCTCCCC
```

>CsFAD5.5

```
1     ATGGCAGCAGAGAAGGAGGTGAAGCCCCATGAAGGCCATAAGCCTTTGGTAATGGATGAATCAAAACAAGAAGAACCAAAGAAAATGGTG
1                                                                                           M  V
91    AAGCCTCCACGATCATTTTGGAGGAGAAACTGGACGACCTTAGACAAACGTGTTGCTTATATCATTTTATTTGTGCATCTGCTTTGTATT
3      K  P  P  R  S  F  W  R  R  N  W  T  T  L  D  K  R  V  A  Y  I  I  L  F  V  H  L  L  C  I
181   TTTGCACCATTTCAGTTCAATTGGTCAGCATTTCGAGTCACCGTTGCATTAATAGTTATCACAGGTCTCTTTGGAATGACTATATCCTAC
33     F  A  P  F  Q  F  N  W  S  A  F  R  V  T  V  A  L  I  V  I  T  G  L  F  G  M  T  I  S  Y
271   CATAGAAATCTTGCTCACAAAAGTTTCCAGCTTCCAAAATGGCTTGAATACTCATTTGCTTATTGTGGAGTTCATTGTCTTCAGgtatat
63     H  R  N  L  A  H  K  S  F  Q  L  P  K  W  L  E  Y  S  F  A  Y  C  G  V  H  C  L  Q
361   atattctcgttattatttgattttcatgatattatatatattaattcactttgtttttgaaagtttgtatcgctttatgatctctttagG
451   GCGATCCACTCGACTGGGTGAGTACACATCGATGTCATCATCAATTCGTTGATTCCGAGAAAGACCCACATAGTCCCATTAATGGATTTT
91    G  D  P  L  D  W  V  S  T  H  R  C  H  H  Q  F  V  D  S  E  K  D  P  H  S  P  I  N  G  F
541   GGATTAGTCACATGATGTGGCTTTTTGATTCCTACACTCTAACCAACAAAgtttgtccaaagtattccagtgatctaaaaaagatagaga
121   W  I  S  H  M  M  W  L  F  D  S  Y  T  L  T  N  K
631   gaaacatgtttgtgatttttacaaagCATGGGAAACCAGACAACGTTGGTGATTTGGAGAAGCAAAGGTTCTATAGATTTATTCACAAAA
138                             H  G  K  P  D  N  V  G  D  L  E  K  Q  R  F  Y  R  F  I  H  K
721   CATATATGCTTCATCATCTTGCTCTTGCAATCATACTCTACAGCATTGGAGGAGTTCCTTTCTTTATATGGGGAATGgtatcaattatta
159   T  Y  M  L  H  H  L  A  L  A  I  I  L  Y  S  I  G  G  V  P  F  F  I  W  G  M
811   aattcactctcatgtttgttctgttttcaataactgatttataaatattattgatgaaaaatagctataataatatttgtatttatgttt
901   ttcagTGTGTGAGAATTGTAGCGGTTTTTCATTCTACATTTATGGTGAATTCAGTATGTCATATATGGGGAAAGCAACCTTGGAGAACTG
185         C  V  R  I  V  A  V  F  H  S  T  F  M  V  N  S  V  C  H  I  W  G  K  Q  P  W  R  T
991   GGGATTTGTCAAGAAACAATTGgtaaatttttttctgttctctaccatatttcatcatggaaagattgaatgatttatctttatagaaga
213   G  D  L  S  R  N  N  W
1081  ataagataatcactataaagaatacaagtgtttgttgatccattgaaaaaaataagtcttgaaaagattcttcatgagtgattcattttg
1171  caatattgcataaatattttagttgttttgtcaattaaaaaaatttcttttgttttaaatatttttattaagaattcaagagtgtttctt
1261  agaaaatacatgaaaactgaatttagaaggaaaaaaaacataaattttaaaaacaaaatactaaaatcaaaatcattacaaaacaagatc
1351  ttgtgtatgtcatatatttttgctctaatttatataaataacaaaaccatatatgatttaacttggtatttaccataatatatatatttg
1441  ggttgtttttgtttaaagGTTGGTGGGTTTGACTTCATTTGGGGAAGGATGGCACAATAACCACCATGCCTTTAGTTATTCAGCCAGGCT
221                     L  V  G  L  T  S  F  G  E  G  W  H  N  N  H  H  A  F  S  Y  S  A  R  L
1531  TGGGCTTGAATGGTGGCAACTTGATGCTGGTTGGTATGTTATCAAGTTCTTACAAGTCATTGGATTGGCCACTAATGTAAAATTACCCTC
245     G  L  E  W  W  Q  L  D  A  G  W  Y  V  I  K  F  L  Q  V  I  G  L  A  T  N  V  K  L  P  S
1621  TCCCACCCACATGCAAAGCCTAGCTATGGATCATAAACCAAAGGACAAACTTTTCTAAATTGATATCTGACATGCATGCAAGGTAGAGGT
275     P  T  H  M  Q  S  L  A  M  D  H  K  P  K  D  K  L  F  *
1711  CTTATATGCTTCTACGAAATTGTTTTGGAATCTGGAGAAGATCAAATGATGTCCTATAATCTTCCATTATTATTTGAAGGATGCTTGATC
1801  CAAATATATTACATTGTTCGGTGATGTTATGGTTGGATTGTTTAAGTTGTTTGAATTATTAGAACATTTATTTGTTTAAGAAAAAGTATG
1891  TAATGTGTTTAGTTTTGGTTGCAGTAATAGAATTAGTGTTATTATTCTACTTTTAAAATCC
```

>CsFAD5.6

```
1     AGTTAATCATAGGGATCAACTAATTAATTTTGTGGTTGATATATTTCATTCAATAAATGATGTGAAAATTTCAGTTGGAAGAGCTCTAAG
91    TTACATGCAGTATAAATACGTATGCTAGAGGAAATGGAATAACATTGATATAGCTTAAGCAAGCCTCGTCAACAATGGAGGGATCAAAAC
181   TAGAACAAAAGGATATGGCGGTGAAGCCTCTAAGGTCATTTAGGAAGAAAAAATGGGCTAAGATTAATAGAAGTGTTGCTGGTTCTCTCC
1                      M  A  V  K  P  L  R  S  F  R  K  K  K  W  A  K  I  N  R  S  V  A  G  S  L
271   TCTTTGTGCATCTACTTTGTATTTTTGCACCATTTCATTTTAATTGGAGTGCATTTTGGGTTGCATTTGTATTATACGTTATCACAGGTC
26    L  F  V  H  L  L  C  I  F  A  P  F  H  F  N  W  S  A  F  W  V  A  F  V  L  Y  V  I  T  G
361   TATTTGGTATTAGTGTTTCTTATCATAGAAATCTAGCACATCGAAGTTTTAATCTTCCAAAATGGCTTGAATACTTATTTGCTTATTGTG
56    L  F  G  I  S  V  S  Y  H  R  N  L  A  H  R  S  F  N  L  P  K  W  L  E  Y  L  F  A  Y  C
451   GAGTTCATGCGCTTCAGGtttgctctttaaccatttcttgttttagtctttttctcttttgaaatatatgtttttctcctaattcta
86    G  V  H  A  L  Q
541   aatttcatactataattctttcacatattccaaaaaatactttgtctttactcgaatgcttttgacttcaaattctaccaacaaaagtaa
631   gtttttcaaaaactatatatatatatttttacttttcaaatttcaacttattttttaaaacaaggaagtggacaacaaaacaaataaact
721   tattgataaaatagtttatataaaaaaaaaattatttctcaaaagaaaaagaaaaaaacaaatgtcaccaaactagcttaaattactta
811   aagtgttttagcatatctatttattagaaaattaaatttctctttatttttaaattttgcagGGCGATCCAATTGGTTGGGTGAAGATAC
92                                                                 G  D  P  I  G  W  V  K  I
901   ATAGATGTCATCATCGATTTGTTGATACAGAAAAAGACCCTCACAGCCCTATTCAAGGATTTTTGTTTAGTCACGTTACTTGGCTTTTAG
101   H  R  C  H  H  R  F  V  D  T  E  K  D  P  H  S  P  I  Q  G  F  L  F  S  H  V  T  W  L  L
991   ATTCTTATGTTTTAACTGAAAAAgttggacctaaatatttgatgattatgaaaagatagaaaggaacgtggttatggtgaacatgaagC
131   D  S  Y  V  L  T  E  K
1081  AAGGGAGACCAGAAAATAACGTTGGGGACTTGGAGAAGCAAGCATTCTATAGGTTTCTTCGGAAAACATATTTTCTTCATCTGCTTCTTC
139   Q  G  R  P  E  N  N  V  G  D  L  E  K  Q  A  F  Y  R  F  L  R  K  T  Y  F  L  H  L  L  L
1171  TTGCAGTTCTACTATATGCAATGGGAGGAGTTCCTTTCCTGATATGGGGAATGgtaaaataaaagctttctcaatcctttgaactcttag
169   L  A  V  L  L  Y  A  M  G  G  V  P  F  L  I  W  G  M
1261  ttgatggtaaataacattatatcggttggattcgtgttgaggaaaacaagcatttatcataatgaggtttgacctctaatatttatcttc
1351  cggttagccaagaattgaataaggaataatgctttcaaccattgatccaaccacataaataatgtcagatctcaatgttatgaaaaccca
1441  atacatcagtaatataacagaaataggtataggataaaagaaaatttatttataaaacttctaacaatacctaccaacgtaatcaaatat
1531  cataatctatctataatagattatgatagatcgatcatgataaataaagatcatagactattatatacgtctatcatgatacaaatagac
1621  acaatataatagtttttatttggtttaccttttgagtaatttttttttgtgagattttatgttttaatctaaaattttggttattgaaaa
1711  aattggtttaaactaagaattgaaaagttatgagaaatattagagtctttatataaaaacaaatgagaaaaaatctatataatttatttt
1801  aaaattggttattttcgagagaaaaattcaaagaaattgattgcgaggaattatgaatttgcattcaaatactcaaggtgaaggtatttt
1891  tgaactttttattaagggtatttttaaactttgaaatttaaaggtactcgtcaacgacttatatccaaaattaatgtttcgaaaatgtt
1981  cttactactatttgttaattataattaatttgatataatttgaacttatataacatatttacttttatggatttaattttttaaaatta
2071  gtacaatttatattatttttacctgttattttttcaataatatatcataatttaagatttttttttataaaataagtattaatttaaat
2161  ttaaaataaaaatgaaacaaaaaggacaataacataaaattgggatgtgggaaattttattttttgtttttgaaattgataccaacgtg
2251  ggtcaatagatacatccaaaaaatctactaaaatattaaaaatttagaaatccacgttttataagaatgtcctaaacttagatattagag
2341  tatcttaaaatactaccaaataaatacgtacaaacgtgtccattccataaaataataaactatccaaatattggtgaaggcattgaaatg
2431  aggagacaaaatgatgtttttactgcgtggggcctctatttaatgtatgagaaagtagattttagcaaagaggttggtttcatgtaaaaa
2521  agaaaattaggttaagaaatagaaaatttccaaactttccatgacttttccaaacacgtataatatcataatttattgctatacttaaaa
2611  gttgcagagttgagacatttcttaacgtacatattaaaagcttatttaatgctaattttgttaatgcacatttcaacagtcatgtttaga
2701  ttatttgcaggatagaaaaataagagaaattatgtatcaaattatttatttgtggtgtagGGTGTAAGAATCGTTGTGGTTTTACACATA
187                                                              G  V  R  I  V  V  V  L  H  I
2791  ACATTTATGGTGAATTCAGTATGTCATATATGGGGAAAACAACTTTGGAAGACAAATGATTTGTCAACAAACAACTGgtaattttctttt
197    T  F  M  V  N  S  V  C  H  I  W  G  K  Q  L  W  K  T  N  D  L  S  T  N  N  W
2881  tctctctccctcgctcaccctttattaagttagaaatgacctataatctcgttagttacatcatttaacactattcttacttatgaattt
2971  gattaatatatgtatataagaaccaaccgatcatgaaaattaacgttaattggaagaaaataacattattacaatagtttgaatacaaga
3061  tccaagcttttgagtcaattctatacatgttgatttgatatggcttaagtatctagatttttttcttagtcatatagttttgtatataacc
3151  tcttgtgtgttgtgtttagGTTGGTGGGTTTACTTGGATTTGGGGAAGGTTGGCACAATAATCACCATGCATTTGAATATTCAGCTAGAC
223                          L  V  G  L  L  G  F  G  E  G  W  H  N  N  H  H  A  F  E  Y  S  A  R
3241  ATGGACTTGAATGGTGGGAAATTGACTTTGGTTGGTATGTTATTATGTTTCTTCAAGCCATTGGAGTGGCCACTCATGTTAAAGTACCCT
247   H  G  L  E  W  W  E  I  D  F  G  W  Y  V  I  M  F  L  Q  A  I  G  V  A  T  H  V  K  V  P
3331  TACAACACCACAAGAAGAAGTTGGCAATGGATGAGACAAAATTTATATGAAAGCCTTCCGAAGACTAAAGTCATCATTTACATAAAAATA
277   L  Q  H  H  K  K  K  L  A  M  D  E  T  K  F  I  *
3421  TATGTTAAACTCATGAATATCTTCTAACGAAGACACAAAATCATGCAATAATTCTAAAAGCGAATAAATGTTATTAATTTT
```

>CsFAD5.7

```
1     TACTATATTATTATAGTGAGACATAACATGCACTATATATAAAATACATGTTGGAGAGAAAATGGTGAAATAATCTAAATTAGAGAGAAG
91    AAAACACTTTGGAAATGGATATGTCAAAACAAGAACTAAATGAAAGGGTGAAGCCTATAATGTCACCAACGAAGAGAGAATGGACCAACA
1                          M  D  M  S  K  Q  E  L  N  E  R  V  K  P  I  M  S  P  T  K  R  E  W  T  N
181   TTGACAAAACCATTGCATGTCTTCTTTTCTTTCTTCATGCCCTCTGTATTTTCGCCCCATTTAACTTTACTTGGAATGCGTTTTGGGTTG
26    I  D  K  T  I  A  C  L  L  F  F  L  H  A  L  C  I  F  A  P  F  N  F  T  W  N  A  F  W  V
271   CCCTTATCTTGTATAGTATTACAGGTCTTTTCGGCATCACTATTTCGTATCATAGAAATCTTTCACATAAGAGTTTCCAGCTTCCTAAAT
56    A  L  I  L  Y  S  I  T  G  L  F  G  I  T  I  S  Y  H  R  N  L  S  H  K  S  F  Q  L  P  K
361   GGCTTGAGTATTTATTTGCTTACTGTGGAGCTCATGCACTTCAGgttttatattattcgataatcttatattgtaattattttaatagga
86    W  L  E  Y  L  F  A  Y  C  G  A  H  A  L  Q
451   tatgataatcttgtacttcatttcttatatatatatatatattatcatattaaattatatcaaagtatatttgagaatgacttaaaac
541   ctaacttttgattgtattgatgtaatttagggtttccatataatcaactcacatatttctctctaaaataaaattatcttttgatttttc
631   ttcattgtttacgttacttttgttgattttatagtcatatatctttttttattttcaaattttgaatttgtgtattttagGGTGATCCGA
101                                                                                  G  D  P
721   TGGATTGGGTAAGTACACATAGATGTCATCACCAATTTGTTGACACAGACAAAGATCCTCATGATCGTAATCAAGGATTTTGGTTTAGTC
104   M  D  W  V  S  T  H  R  C  H  H  Q  F  V  D  T  D  K  D  P  H  D  R  N  Q  G  F  W  F  S
811   ATATCAATTGGGCTTTCGATTCCTACCATTTGACTAAAAAGGTTTGTGGTAAgtacttcaatgattccaaagaaacaaaaagaaacttgt
134   H  I  N  W  A  F  D  S  Y  H  L  T  K  K  V  C  G  K
901   tcacattagtaatgaagcatgagagACCAGATAATGTCAAGGACTTGGAGAAACAAATATTCTATACGTTTATTCATAAAACGTATATTC
152                                     P  D  N  V  K  D  L  E  K  Q  I  F  Y  T  F  I  H  K  T  Y  I
991   TTCATCCAATTGCTTTGGCAATCTTTCTCTATATGGTTGGAGGACTTCCTTTTGTTTTATGGGGAATGgtaaaataacatctcactacta
173   L  H  P  I  A  L  A  I  F  L  Y  M  V  G  G  L  P  F  V  L  W  G  M
1081  tcaataaatcttattatttatttttattatcttcatcaacatggtaatttaattttatatagtaaaagggaaaatggaacaaatagtaag
1171  aaataaatcattgattaattcctaattcaggaacgtgttttgctagtcagaatttcaaatgtactttttttcttgacttatatagttgtt
1261  ggcttcaaaagtgactttgtaataagagagaaactaaaaaataatttaatgtcaaatacatagattatataattgaaattatagccctag
1351  tttatggtaaatctcgtgtattttaggaaatatatatttgtattgacgtacctaaccttactgtaccttttgatgcagTGTGTGAGGATA
196                                                                                 C  V  R  I
1441  GTAATGTTATATCATGTAATATTTATGGTGAATTCAGTATGCCATAAATGGGGAAAGAAACAATGGAGTACAACTGATTTATCTAGAAAT
200    V  M  L  Y  H  V  I  F  M  V  N  S  V  C  H  K  W  G  K  K  Q  W  S  T  T  D  L  S  R  N
1531  AATTGgtaaattctttgtgccttcttttcaactttggaagatgattaaatgaagcgcccttttggctcatgagcatgatgatggagctta
230    N  W
1621  agggtatcaacttagttgagagatgcatgggttttcttacggattcattttccttgagtatcgtgttaaaaaataaaaagtatcattgct
1711  tgatataaaaacctttaaaagattatcaaacaaggtttcaacctaattaagatgtcttaatacatttacttatctattcttttagtatct
1801  tattaaacgggagagtatatatgctcaattttttcacctccaagttaaggattgaatgtttattatatatattgattattggttaattt
1891  tagGTGGGTGAGTCTTCTTTCTTTTGGAGAAGGTTGGCATAACAATCACCATGCATTCGAGTATTCAGCTAAGTATGGACTTGAATGGTG
232       W  V  S  L  L  S  F  G  E  G  W  H  N  N  H  H  A  F  E  Y  S  A  K  Y  G  L  E  W  W
1981  GCAACTTGACTTTGGTTGGTATGTTATTATGTTCCTTAAAGCAATTGGAGTGGCCACAGATGTCAAATTACCCACTCAATGCCACAAGCA
262     Q  L  D  F  G  W  Y  V  I  M  F  L  K  A  I  G  V  A  T  D  V  K  L  P  T  Q  C  H  K  Q
2071  AAGGATGAAACTGTGAGCTCATCAATGGATGAATTTAAAAAATGATGCTTAATTCATACTGAAAGATATAAACACGCCATATAGGATTTA
292     R  M  K  L  *
2161  CATAATTATTGTTTGAATTAGATGTATAATCCAACGATATATAATATTCATGTATTAGGGATATAGGATTTATGAAGAAGAATGAGATAT
```

>CsFAD5.8

```
1      ATGAGTAATTGGGTTGAGCTAGATCAAGGTTCTCTTCTTATGGATACATTAAAAGAAGAATCAAAGAAAATGGGGAAGGTTCATCAAATG
1                                                                             M  G  K  V  H  Q  M
91     ACATTTGGGAGGAGAAAATGGACCAATGGAGACAAATTAAAAGCATCTCTCATTTTATCTTTTCACGTTCTATGTATTTTTGCACCTTTT
8       T  F  G  R  R  K  W  T  N  G  D  K  L  K  A  S  L  I  L  S  F  H  V  L  C  I  F  A  P  F
181    CATTTTAATTGGAGTGCATTTTGGGTTGCGTTTGTATTATATATTCTCACAGGTCTCTTTGGAATAAGTATTTCTTATCATAGAAATCTT
38      H  F  N  W  S  A  F  W  V  A  F  V  L  Y  I  L  T  G  L  F  G  I  S  I  S  Y  H  R  N  L
271    TCCCATAAAAGTTTCAAACTTCCAAAATGGCTTGAATATGTATTTGCATATTGTGGAGTTCATGCAATTCAGgtttgaccttttgaacc
68      S  H  K  S  F  K  L  P  K  W  L  E  Y  V  F  A  Y  C  G  V  H  A  I  Q
361    atcctttgtcttgctattttgtttgaatctatttttatttgaattaaacattagaactttctatattattttataacgttgagattaga
451    agtatgtttatttacctcaaacagacccatcttaaaagagaagatgagaaagatgggtggggatctatcggtttaaatactatttttgtc
541    accataattctagttttggttcttttccgctcttgtactttaataaatttcattgtcgcaactatatataatcttttagcctaattcgtt
631    caattttgtttattttattctttgatgtatttttgaaaaatgaacctccaaaggtagaatatgatgacttttgaaagtataaaattcaac
721    ccattaacattttcaaaataaattaaaactaaaaatacacaaaaagactaaagtagtatatttttttcaacttgaatatatcttaatgt
811    tatttttcaaatcttttattccataactgccatctataatctaaaatattgaaataagtttcatatcttgttgtttggttcattttaaa
901    tatttatttttctttctaattgtagGGTGATCCAATCGACTGGGTGAGCACACATAGATATCATCATCAACATACAGATACCGAAAGAGA
92                               G  D  P  I  D  W  V  S  T  H  R  Y  H  H  Q  H  T  D  T  E  R  D
991    TCCACATAGTCCTGTACAAGGATTTTGGTTTAGTTACGTCATTTGGATCTTTGATACCATTACTTTAACCAAAAAAGTCTGTCCAGAgta
114      P  H  S  P  V  Q  G  F  W  F  S  Y  V  I  W  I  F  D  T  I  T  L  T  K  K  V  C  P  D
1081   ttttattgattataaagataaaaaaagaggtgccttttccttggttactaaatatggaagTCCAAATAATGTTGCTGATATGGAGGATGA
143                                                                  P  N  N  V  A  D  M  E  D  D
1171   TCCATTTTATAGATTTATTCATGATACATACTTACTTCATCCAATTGCTCTTGGAGTCCTTCTCTACATTGTTGGAGGAACACCTTTTTT
153      P  F  Y  R  F  I  H  D  T  Y  L  L  H  P  I  A  L  G  V  L  L  Y  I  V  G  G  T  P  F  F
1261   TATATGGGGAATGgtaaataaatctctctccaaaacatgcataaatttatttttaaaaaaaagggtttcttttactatcaattttaata
183      I  W  G  M
1351   tatatatctacatatataaaggtttatgacaattagggatatattgatatgatctctattgattctgcagTGTGTGAGGGCAACAGTGTA
187                                                                           C  V  R  A  T  V  Y
1441   CATGAATGTAGTGTTTATGATTAATTCAATTTGTCATTTATGGGGAAAGAAACAATGGAACACTAAAGATTCCACTAGAAATAATTGgta
194      M  N  V  V  F  M  I  N  S  I  C  H  L  W  G  K  K  Q  W  N  T  K  D  S  T  R  N  N  W
1531   agtgctttatcgtgaattcttgtcatttatttttcttgtcatttatttacctcaccaaatacaatcaaaatagtttcgaattacgatttg
1621   taacgattgagtctttttatatcgtaaaaaatatcattatagtttaagaaaattgcttacacatgtgttgagttgataaattgattagaa
1711   catgtcttgagttaataaattgattagagataatttttttattattaataataaactataaaaatagtgatagaaatgactgtcagatta
1801   taaatttaattatgtaattctattatggactttaatgagtagaaaacttttgatttaagagtataatgaaggtgttaataagaatgtcaa
1891   cctagtaattgaaatgtctaagtatgtttaaaaactctcttcctctattattttattcgaaaaaaggaaaaataacttttaaatatttat
1981   tataaatagaagtcaatatcattatcaaaccagagacctaaattaataattaaaatagataatctatttttttaaaaaaataatctattt
2071   ttttaaaaaaatagataattacttactatttgaactgatacatacctttcacaactcatgtaacattagaagaaaaagacactatacacc
2161   atcgattattgtaccattatcatttaagaactaggtaaactgatagaggtgtgggtggttattgatgaataccacagttgtttagcaata
2251   tacaatgaagccagtactatatcttgtatgttaaaaagttgtatatatattatatttaatgaaatctatcatgattgtttgatttttgtt
2341   gcatttgctcagGTGGATTAGTTTGCCTTCATTTGGAGAAAGTTGGCACAATAATCATCATGCGTTTGAATATTCAGCAAGAGCGGGGAT
223                  W  I  S  L  P  S  F  G  E  S  W  H  N  N  H  H  A  F  E  Y  S  A  R  A  G  I
2431   TGAATGGTGGGAAGTTGACATTGGTTGGTATGTTATATTGTTTCTTCAAGCCATTGGGTTGGCCACTGATGTCAAACAACCCTCTCAAGC
249      E  W  W  E  V  D  I  G  W  Y  V  I  L  F  L  Q  A  I  G  L  A  T  D  V  K  Q  P  S  Q  A
2521   TCACATGCAAAGGCTATCTATGGACAAACCAAATGAAGACTGTCAAGAATGA
279      H  M  Q  R  L  S  M  D  K  P  N  E  D  C  Q  E  *
```

>CsFAD5.9

```
1       GCCCATTGAACATGCAAACAGTACAGAAACCAAAGGCAATGGGGAAATCCCCAAGGTCATTCAAGAGGAGAAAATATACCAAGTTGGACA
1                  M  Q  T  V  Q  K  P  K  A  M  G  K  S  P  R  S  F  K  R  R  K  Y  T  K  L  D
91      AAATCATTTTCTTTTCCCTATTCATCGTCCATATTGCCTGTATTTTTGCACCATTTCATTTTACTTGGCCTGCATTTTGTGTCGCGTTTG
27      K  I  I  F  F  S  L  F  I  V  H  I  A  C  I  F  A  P  F  H  F  T  W  P  A  F  C  V  A  F
181     CATTGTACTTTATTACTGGCTTATGTATTAGCGTTTCGTACCATAGAAATCTTGCACATAAGAGTTTCAAACTACCAAAATCAATGGAAT
57      A  L  Y  F  I  T  G  L  C  I  S  V  S  Y  H  R  N  L  A  H  K  S  F  K  L  P  K  S  M  E
271     ACTTTCTCGCTTATTGTGCGGCACATGCTCTTCAGgtttattaacacgtgatcaacttctacttctttttttgtttatgaaatgttatata
87      Y  F  L  A  Y  C  A  A  H  A  L  Q
361     tctatttttaaaattatttgttatttatttatttatttagGGTGATCCAATCGATTGGGTGAGTACACATAGATGTCATCATCAATTTG
99                                              G  D  P  I  D  W  V  S  T  H  R  C  H  H  Q  F
451     TTGATACAGAAAAAGATCCACATAGCCCTATCGAAGGATTTGGTTTAGCCATATCACTTGGCTTTTTGATTCTTATAATTTGACCAAAA
115     V  D  T  E  K  D  P  H  S  P  I  E  G  F  W  F  S  H  I  T  W  L  F  D  S  Y  N  L  T  K
541     AAgtttgtccaaattattttaccgattttcaaaaagttgatagaagcatattcatatggttctccaagCATGGGAGACCCGATAACGTTC
145     K                                                                   H  G  R  P  D  N  V
631     GTGATTTGGAGAAGCAAACCTTCTATAGGTTTATTCATAAAACATATTTTCTTCATCACATTCTTCTTGCGATCCTACTCTATCAAGTTG
153     R  D  L  E  K  Q  T  F  Y  R  F  I  H  K  T  Y  F  L  H  H  I  L  L  A  I  L  L  Y  Q  V
721     GAGGACTTCCATTTCTTATATGGGGCACGgtaaaggtttcatttataaactaactttatataaactttcatgtattcctcttgataatta
183     G  G  L  P  F  L  I  W  G  T
811     acatatctaatatgtaacattattttttttattatatggaaagttgtgtttaaaataccaaaatttaagatataatcatgtaatgtctcac
901     gtccaagatttggagtcgatcagattcggtatctaaggactttaacattctcttatatttctagtagatgtggcttagtgacaaaccaag
991     ataaagaaaacgttggtggacaatgtgaccatggtagaataggttattatctataccaacaaatatatatatatatatatatatatttag
1081    tagctcaattgaagaaatcttatattggatgactttctatagatttttttaagaaacgtgggagtgaggacactaaaataactcaacatta
1171    gcttttgtagagataggacacaacactatcaataagttcaaaaaaagtatatctgatgatctaactagaggatttggaaaggatacgtga
1261    gagaatgtcaaagaatcatcatagttttaaatccaacgtttagaataatccaagtcataatcgttggacttgagacgttataatgatagt
1351    cataattctttctcttccatttaatcaagcatttcgattagataatagatatgcaaattttcattttgggctaacgaaaataatttctta
1441    actttaaattatgtagTGTGTAAGGATCGTAGTGCTCATGCACGTAACATTTATGGTGAACTCGGTATGTCATATATGGGGAAAACGTCG
193                     C  V  R  I  V  V  L  M  H  V  T  F  M  V  N  S  V  C  H  I  W  G  K  R  R
1531    ATGGAATACTAAAGATTTGTCAAGAAACAATTGgtaagtgcttctatatcattctttattttcattaatcaaatgtgagatattatttaa
218      W  N  T  K  D  L  S  R  N  N  W
1621    atagttgattttatccttaatctccatttcaatatactaaattggattaggtttgaaaggactatttaagtattaatgagaaactttctt
1711    tgacactttaagctcttaaaaaattattttaaatacgttctaagtttgaacaatcatgaaatagaaattctacactttgcttttaaatgt
1801    actattgttttatatacactctaagattgaaaaaaaaaagatcaaatgtggtcctcgaagcacatccaataaaattggaatgatacataa
1891    atattaccatggcttcatacgatgggaaaatggtacaaattgtttaaatagatcgaaaaaacaacgtgtggatgagaaaaaataaaaata
1981    atttctttaaaaaaaataaaaaagtaaaaataaaagttttgtttatctacactaacacttctcaaatagagatactcttcctatcaatt
2071    atattttttattattgacttagtgaattgtgattgtaagactttttttatggcattatttccaaccgttaccattcaaaacttgttgaaa
2161    gaattccccaaccatattatatagtaattgccaattaatatgtatgtctccttttttagctagcctaggcatatactatataattatgatt
2251    tactatttatttgttagttttcctttattcttttaaagGTTGGTAGGTTTGCTTGCATTCGGTGAAGGTTGGCACAACAACCATCATGC
229                                           L  V  G  L  L  A  F  G  E  G  W  H  N  N  H  H  A
2341    TTTTGAATATTCAGCAAGGTTTGGACTTCAATGGTGGCAAATTGATTTTGGTTGGTATACAATTAGGGTTCTTCAAGCCATTGGAGTTGC
246      F  E  Y  S  A  R  F  G  L  Q  W  W  Q  I  D  F  G  W  Y  T  I  R  V  L  Q  A  I  G  V  A
2431    CAAAAATGTTAAACTACCTTCTACAATTCACAAACAAAGGCTTTTTATAGACTATTGAGTCTGCAATAAAAAAATCTATTAATGTTTGGT
276      K  N  V  K  L  P  S  T  I  H  K  Q  R  L  F  I  D  Y  *
2521    ATTCTCAAGACGATTTGTTTGTTTTTATTTTGTGT
```

>CsFAD5.10

```
1     GGAAGTAGCCAAGGTGGGAAGGTTCTACCTATTGGCTAATTTCTTGTGCTTGTTTTCATCTATATGGAGGCAGCAAGAAAAGAGGTTGAA
91    GATAAACATGATAAGAAGAAGCAATTTTCAATGGTGAATCCTAAACGTGCATTTTTCAACAGAAAATGGACAACTTTAGACCGACGTACT
1                                          M  V  N  P  K  R  A  F  F  N  R  K  W  T  T  L  D  R  R  T
181   GCTGCTGGAATTTTGTTCTTGCATTTGCTTTCTCTCTTGGCCCCATTTCACTTCAATTGGGCTGCATTTTGGGTTGCTTTTGCACTATAT
21     A  A  G  I  L  F  L  H  L  L  S  L  L  A  P  F  H  F  N  W  A  A  F  W  V  A  F  A  L  Y
271   TTGATTACAGGTGCCTTAGGGATTACGCTCTCGTATCACAGGAATCTTGCACATAGAAGTTTTAAACTTCCTAAATGGCTTGAGTACTTT
51     L  I  T  G  A  L  G  I  T  L  S  Y  H  R  N  L  A  H  R  S  F  K  L  P  K  W  L  E  Y  F
361   TTTGCATATCTTGGAGTGCAAGGCTTTCAGgtttgctgtattattatggtattactgtgttgtttgttttataattaaagtcattactga
81     F  A  Y  L  G  V  Q  G  F  Q
451   caagggtggtattgattaagatttggtaattaattaaccttgttcgttgtatgatgtcttgagcttcgttctctgtagtcttgttctttg
541   tatgatctcttaagctcctctccggctcttcttttgtgtactcgccttttgtgatataaatataaggtagggtttgtaagaaatgtgtcc
631   aacatagttaacatatttggttgtacgtacaagcctactaatccttcaatactttattctcttctttaaaaaataaaaagttatttgtaa
721   cattttatctcttcgtttaattttgctatagGGTGATCCAATTGATTGGGTTAGTACGCATAGATATCATCATCAGTTTGTTGATACCGA
91                                          G  D  P  I  D  W  V  S  T  H  R  Y  H  H  Q  F  V  D  T  D
811   TAGAGATCCACACAGTCCCATGCAAGGATTTTGGTTTAGCCACATGACATGGATGTTTGACTCATATGGTTTGACTGAAAAGatttatcc
111     R  D  P  H  S  P  M  Q  G  F  W  F  S  H  M  T  W  M  F  D  S  Y  G  L  T  E  K
901   gaaaaatctcgaagatttcaaggaaagagaagcaaaacgaacactttctcgcaattggtttgtgggttttctaaagTATGGAAGGCCAAA
138                                                                                Y  G  R  P  K
991   GAATGTTGAGGATTTGCAGAAACAAGGTTTCTACAGGTTCCTTCGTAAAACATACCTTCTTCATCCCATAGCTCTTGCAATTGTTCTCTA
143     N  V  E  D  L  Q  K  Q  G  F  Y  R  F  L  R  K  T  Y  L  L  H  P  I  A  L  A  I  V  L  Y
1081  TAAATTTGGAGGACTTCCCTTTGTTGTTTGGGGAATGgtaagtattaacacaagggaaaaagatagaaaaatgcaattcttttgtttgcc
173     K  F  G  G  L  P  F  V  V  W  G  M
1171  ttcattattcttaacatgttagcttctccatctccctgtttattgtgggattgaaaagtttgaaaagttatctccaagtaaataaaataa
1261  aataaaataatttaaatgataaagcagttaaaaatatctataaatataataaaactttaccttcacaattaattataaatgcaatagat
1351  acaaataaacattcgtttatgtttgatctaaagcgatagatatccatttaagtctatcacggtctatcacatatatatttaaattttgtt
1441  catttattggtttgaactagaatttttatagtttttcttttaatacaaacatatataatttagtactttttttaaaacaaaacaaaatca
1531  aacctactcaaatttcacaaacattaagtggaaatttgaattttatgactttaattatatttaatctatcataaggttatttttaagtat
1621  aacaaaatgaatcaaaataattctaaaatatataaaaaattctatgaaattttgttatatttaaagggtttttcaaattgaaacaatt
1711  tattctttttatttattactttttttttttttttgttactaaggtattgcattcaattgttggctttgacaatttgacattcagaagga
1801  gatggtaaaataataaaaataataagagattgattgtgaattgtgatgggcagGGAGTGAGGCTGATTTGTGTTTTACATGCTGCACTCT
185                                                         G  V  R  L  I  C  V  L  H  A  A  L
1891  TCGTGAATTCAGCATGCCACATTTGGGGCTACAGAAAGTGGAACACCAATGACTTATCAAAGAACAATTGgtctcttctctcctcctttt
197   F  V  N  S  A  C  H  I  W  G  Y  R  K  W  N  T  N  D  L  S  K  N  N  W
1981  tcttatcatccatccatagttgtttttgttaaatttcctaaagttttaggtttttaacaattctgatcttattttgtttatactcctttt
2071  caaaagtaatatctatattcatttcaaattttaattatggttttcacatttcttaataataataataagtatttaaaaattgctaactg
2161  aattataaaaacaaaaatattaaaatatatagtttttaaaaaagaatttgaaacgttaaaagaaaatcaataacaagtaacaatatagat
2251  atagaatgttaggggtagaagtagattttatagacttaatttttaaaataaaaaacaaaagatagatttatatttctaagctaaggga
2341  gatccatggcttgtttaagatctctaacgtaagtttgatttatttgagattttacaagtttaaaagatacctgtttgacaaaggtcaagg
2431  agctattaattctttttgtttaaagtgtgtggtacacaaatatacatgtaaaagtttcattctctcttctaatatttaggatggttttg
2521  taaattaattaatgtttttgtgtatgtgtttgagagacagGATAATAGGATTGATTTCTTTTGGGGAAGGTTGGCACAACAACCACCATG
221                                                     I  I  G  L  I  S  F  G  E  G  W  H  N  N  H  H
2611  CTTTTGAATACTCAGCTAGACATGGTCTTCAATGGTGGGAATTTGACTTCACGTGGTGGGTTATCTTATTACTTCAAGCTCTTGGATTGG
237   A  F  E  Y  S  A  R  H  G  L  Q  W  W  E  F  D  F  T  W  W  V  I  L  L  L  Q  A  L  G  L
2701  CAACTGATGTCAAATTGCCCACTCAACTTCATAAGCAAAAGTTGGCCATCCAATATAAAATCACTTCACCATGTGATTCCTCTTAAGTAT
267   A  T  D  V  K  L  P  T  Q  L  H  K  Q  K  L  A  I  Q  Y  K  I  T  S  P  C  D  S  S  *
2791  TATTGTGATCTTTTTGCATTTATTTAGTTCTTGTTCTTGCTTGTTTTTTAAATAAAAAAGAAAGAAAAAGAAAATAAATATGTACATGGA
2881  GTCTTATATGACTTCTTAAATCTTTTTATGTCATTCGAGTATTATGTTTTCAAACATTATGGATTATTTTCTCTCCATAATATATTTATT
2971  AATGAGAATGATCTTTAGATTATA
```

>CsFAD5.11

```
1       GCAGTCTTTGCTTTTAAAACGTTCCTATACTACTCTTTATCGATCTTTGTTTTTTTATTTTGCCATTACTCTCTCTTACTCCTCAAATGG
91      AGGCCACAAGGAAAGAGCTGGAAGATAAAAATAGCGATCAGAAGGCTATGGCGGTTGAAGACAAACCTCGGGTTAGGTCCCGGCGAAGAC
1                                                       M  A  V  E  D  K  P  R  V  R  S  R  R  R
181     CATTTTTCCGAAGAAAATGGACAACTTTAGACAAACGTTCTGCTGGTGCTTTTCTTGTTATGCATTTTCTCTGTGTTTTCGCACCATTTT
15      P  F  F  R  R  K  W  T  T  L  D  K  R  S  A  G  A  F  L  V  M  H  F  L  C  V  F  A  P  F
271     ATTTCACATGGCCAGCCTTCTGGCTTGCCTTTGTACTCTACGTTATCACGGGCTTGTTCGGTTTGACACTCTCGTACCACCGGCATCTTA
45      Y  F  T  W  P  A  F  W  L  A  F  V  L  Y  V  I  T  G  L  F  G  L  T  L  S  Y  H  R  H  L
361     CACACAAGAGTTTCAAACTTCCCAAATGGCTTGAGTACACGTTTGCTTACATTGGAGTCCATACACTTCAAgtacaccttcactttttt
75      T  H  K  S  F  K  L  P  K  W  L  E  Y  T  F  A  Y  I  G  V  H  T  L  Q
451     tttctttttaattgttttcttgacaatgtttaaattataatctatttttcggtgtgtttatagGGCGATCCGATTGATTGGGTGAGTAC
99                                                                       G  D  P  I  D  W  V  S  T
541     GCATAGATATCACCATCAGTTTGTGGATACAAAAAGAGATCCACACACTCCGACACAAGGGTTTTTATTTGGACATATTGCTTGGATATT
108      H  R  Y  H  H  Q  F  V  D  T  K  R  D  P  H  T  P  T  Q  G  F  L  F  G  H  I  A  W  I  F
631     TGATTCATATGGTTTGACTAAAAAAgttagccctaaacatgttgaagatttcgaagcaaaagatgagaaaagaaagattagtagaaatat
138      D  S  Y  G  L  T  K  K
721     gtttgtgggatatttgaagTATGGAAGACCTAACAATGCTGAGGATTTACAAAATCAAGGCTTTTACAGGTTTCTTCGAAGAACATACAT
146                         Y  G  R  P  N  N  A  E  D  L  Q  N  Q  G  F  Y  R  F  L  R  R  T  Y  I
811     TCTTCATCATTTTGGTCTTGCAATAATCCTCTATGCTCTTGGAGGGCTTCCTTTTCTCACTTGGGGAATGgtaaacccagttattctagc
170      L  H  H  F  G  L  A  I  I  L  Y  A  L  G  G  L  P  F  L  T  W  G  M
901     actaattttgttatttttattttaagtcgagttgatccaaaaaaacatcattttcaaatgttaattttaatatatacttctaggtcag
991     aagatcacgtaagaattacaaatgtgtcaacttaattatataaatcaatctcctaaaataatttacttgtcatattttgttttagaaaaa
1081    caaaagtcaaatcaaacactcttaattagatctaaaaattttatacttgggtttgtcccggttacaatttaaaatcctattactattatc
1171    atgatcattagaagtaattttaaatatagtgaaatgaactaaaatatttacaaaatataataaaattttaactctattattgatagacag
1261    tcatatatttgtatctctcatcatcgatagaatatgaagatttagtatattttagaaatgttttctaatagttttatcatttacaatatt
1351    atttaatgttattattactactactacatgttagcttaaaatctaacgtacacaagttgaaatatatgtgtcaacctagttaacgtgtgt
1441    tctatatgtgtctaacttatttgacatatgctagtgtgtcgactaattgcacatacatccaaatgtttttagaaataaaaaacctaatat
1531    acaccgattattttgcaataaatccaagtttttaacctatatttcctaaaactaatcactgtcaactgacatataaaatatacgtcagg
1621    tacgtaattaaatatacatatatatatcttttatgcaagctttggaagtgaggttgaaaaacattatttatttgtaattttcagtacata
1711    aacatcatatatgttgatgttaacattacaaagaatgtttgaatggaagggttgaattgttgaataaatgcattgaaaatgcagGGTGTA
193                                                                                  G  V
1801    AGAACTGTATGCTTTCAACATGTCACTTTCGCTGTGAACTCTTTAGGCCACATGTGGGGAAACCAACCATGGAACACTGGAGATCAATCT
195      R  T  V  C  F  Q  H  V  T  F  A  V  N  S  L  G  H  M  W  G  N  Q  P  W  N  T  G  D  Q  S
1891    AGAAATAATTGgtgacgttcacacgttcctttcattttacattacagttcttttaagatttgtctagtcatttttctcggatattgga
225      R  N  N  W
1981    tttgttcttgtaacgtttcggttctagagacgtatataaatgaacctagatcacacacgatgaaatggatattatagtatattataatct
2071    aaaaatctataacgagtgtatttgatttgaaagaaaacaagaaaaaaaagtatataattgattttatatttccaactatgctttggcta
2161    aactaggatgacttttgaccatctttaatttttaagactttgccaccagttggactgatatagaatttgtttggattttagGGTGTTGGC
229                                                                                   V  L  A
2251    ATTACTCATCTTTGGAGAAGGATGGCACAACAACCACCATGCATTTGAGTATTCAGCTCGACATGGCCTAGAATGGTGGCAGTTGGATGT
232      L  L  I  F  G  E  G  W  H  N  N  H  H  A  F  E  Y  S  A  R  H  G  L  E  W  W  Q  L  D  V
2341    AACTTGGTGGGTTATTTTGTTTCTTCAAGCCATAGGGTTAGCCACTGATTTGAAGTTACCCTCTCAACATCACATGCACAAGTTGGCCAT
262      T  W  W  V  I  L  F  L  Q  A  I  G  L  A  T  D  L  K  L  P  S  Q  H  H  M  H  K  L  A  I
2431    CCAACCCAAAAGCGATTAATGATACTAAAACACTATTTAAAATGACTTTTTAAGGAATTAAAAATTAATTATTTTAAA
292      Q  P  K  S  D  *
```

>CsFAD5.12

```
1     CTGAGTTGAGACATCGGTGCTTTCAAAACCTCCCATACTACTGTTTATCTTTGTTCTTATTTTAATTTTCCATTAATGTCCCTTAGTCCT
91    CAAATGGAGGCCACAAGAAAAGAGCTGGAAGATAAAAATAATGATCACAAGGCTATGACGGTAGAAGGCAAAGCTCGAGTTAGGCCTCGA
1                                                                          M  T  V  E  G  K  A  R  V  R  P  R
181   AGAAGAAGGCCGCTTTTTCGAAGAAAATGGACACTTTTAGACAGACGCTCTTTTGGTGGTGTTATGGCTATGCATTTGCTTAGTCTCTTG
13     R  R  R  P  L  F  R  R  K  W  T  L  L  D  R  R  S  F  G  G  V  M  A  M  H  L  L  S  L  L
271   GCACCATTTTATTTCACATGGCCAGCCTTCTGGCTTGCTGTTGTACTCTACATTCTCACAGGCTTGTTCGGCGTGACGCTCTCATACCAC
43     A  P  F  Y  F  T  W  P  A  F  W  L  A  V  V  L  Y  I  L  T  G  L  F  G  V  T  L  S  Y  H
361   AGACAACTCTCGCACAAAAGTTTCAAACTTCCCAAATGGCTCGAATACACATTTGCATATATGGGAGTTCATGCTCTTCAAgttagtaaa
73     R  Q  L  S  H  K  S  F  K  L  P  K  W  L  E  Y  T  F  A  Y  M  G  V  H  A  L  Q
451   tattttactagtcaatgttcttgccattttcttttctttttccattcttttgtgatttatatttgtgtgttttatagGGCGATCCAATC
100                                                                                      G  D  P  I
541   GATTGGGCGAGCACACATAGAATTCATCATCAGTTTGTTGATACGGAAAGAGATCCACATAGCCCATCAGTAGAAGGGTTTTTCTTTGGA
104    D  W  A  S  T  H  R  I  H  H  Q  F  V  D  T  E  R  D  P  H  S  P  S  V  E  G  F  F  F  G
631   CATATTGCTTGGATATTTGATTCATATGGTTTGACAGAAAAAgttaatccaaaatatgttgaaagttttaaggaaagagatgaaaaaaga
134    H  I  A  W  I  F  D  S  Y  G  L  T  E  K
721   aagacatttagcaatatttttgtgggatatttgaagTATGGAAGACCTAAAAATGTTGAGGATTTACAAAAGCAAGCCTTTTATAGGTTT
148                                         Y  G  R  P  K  N  V  E  D  L  Q  K  Q  A  F  Y  R  F
811   ATTCGAAGCACATACATTCTTCATCATTTTGGCCTTGCAATTATTCTCTATGTTGTTGGAGGCCTTCCCTTTCTCGTTTGGGGAATGgta
166    I  R  S  T  Y  I  L  H  H  F  G  L  A  I  I  L  Y  V  V  G  G  L  P  F  L  V  W  G  M
901   agatatctgttcttctaccacactaatatttttactttactttttattaggtcgatttaatttaattaggttttagctagagaaattttat
991   tttagtttagtagtaacaaaaatatataattatagttaatctatagtaaaacaaactaaaatatttataaatacaaaaaaatatatatat
1081  cataaagtagtaccaaagtaatcttgatggaattcaaccacttacgaactttctatttagtgtacaaataatagatagtcacatataata
1171  gtatatattttgttatatttataaatattttcaactgttttatcatttaaaataacttttctaatggtaatagagatattttcgtataga
1261  gcaacatttagtcgttttgatataaatgtgataatagtttctaaactttgttatttaaaatagttaataatcataataataagtttacac
1351  tacatgttagcttgttattattttaaatgtaaaatagtttagaataataattgttataattaattttacacagcatgttagcttattcat
1441  ttcaccctatacaagtttgcaaaaaaaaaaaaaaaccaaagttttctaattttgttttgccaaataaggacgatagcacaattgattta
1531  tgtgtgtgtatatataatcaactaacatgtaaaggatttgaatcttgaagccacattattatggaatttaatatgtataagccacataat
1621  gtacatgtttaattttctaaaatgaaagcgggttaaaaggtaattattataaatgaagggtaaaatgagaggttgtaaaaatagcaaaag
1711  aaaaaaaaaacgataatataatattacagtgtatatcacttacttttcaaatttgtaaaaataataaatttaaaagtatattatcgtct
1801  gatttgtcaaatttacaatataaaaaaaaaagtgttacgaactgtttttttctaaactttttgtcatctaatacaattctcataataaa
1891  attgttgaaaatatttacaaaatattacaagattttatattctatttataaagtgttatagacatgttattagtgacattgatagaagtt
1981  tattaatttctcgtataaaatttagattttactatatttgtaaatattttatattattttatcatattcgaaaaaaaatctcgtcaac
2071  atgaaataattataattggtagtagttttgagaataataattacgtacaacttatttttcaaatagcaaatataacaaaatttattctc
2161  aacaaataattatgtatcatctttagactttttttaaaatatcgttgtatatttaattattttaaatctaaatactatatatatattttt
2251  tttttggtaagaagtgggagtgggtataaaaatggtaattaaaaataacaaagagtttaagacacattaattagcaactaattttttataa
2341  gtggatgacagctgtaattagtaacacaatatataattggttttaattattgggtccatttatttattactattttcttttgttgttaa
2431  caaaatatatagggggaagatggagtgttttgagtcacagggttaattagatatgaatacttaatttgctgcaaaatcattagttagatac
2521  acaataatctatattctctttttgtacaatatttgaagtggggataaataacattaattatttgttcattcatatgttaattacatcaga
2611  aagaatatttgaaaattgaaaggagaggggggtggtgagaaaagggtttcatgaattaattatattgaataaataatggcattgatttga
2701  ttatttgttgtgtgcagGGTGTAAGAATTGTATGCTTTCAACATGTCACTTTCTTCCTAAACTCTGCAGGCCACTTGTGGGGAAGCCAAC
195                     G  V  R  I  V  C  F  Q  H  V  T  F  F  L  N  S  A  G  H  L  W  G  S  Q
2791  AGTGGAACACTGGGGATCAGTCCAGAAATAATTGgtgatgtttaatgtttatctttctttttcatattttctctttaaagaaacttttag
219   Q  W  N  T  G  D  Q  S  R  N  N  W
2881  gaggattacatgcataaatataatattcatgatgttagatcacataccccgattgtatcaaagtatgaccaggaggaatgcttaaaataa
2971  tgaatgactagccattatccatatacactaataactctattttctttcaaattggacttgagatggaatctcatcctatcatataggttt
3061  tacctatttccatcaattgatatcaaagtcatagttggtgtggctaagacatgtaacaattttgaataaaggttgatagtctataataat
3151  ctaaatgaatattcaattgatatattttttattgaactagtagtttcacttttttagGATGGTGGCATTGTTTATGTTTGGAGAAGGATGGC
231                                                                          M  V  A  L  F  M  F  G  E  G  W
3241  ACAATAACCATCATGCATTTGAGTATTCAGCTCGACATGGCCTAGAATGGTGGCAGTTCGACGTAACTTGGTGGATCATTTTGTTTCTTG
242   H  N  N  H  H  A  F  E  Y  S  A  R  H  G  L  E  W  W  Q  F  D  V  T  W  W  I  I  L  F  L
3331  AAGCAATAGGGTTAGCCACTGATGTGAAGTTGCCCTCTCGAAATCACATGCAAAAGTTGGCCATTCAACCTAAGATCGAGTAAATAATAG
272   E  A  I  G  L  A  T  D  V  K  L  P  S  R  N  H  M  Q  K  L  A  I  Q  P  K  I  E  *
3421  TAAAACATGGTTGAGAAATGATTTTTTTAAAGGTT
```

>CsFAD5.13

```
1     AAATAGTTAAAGATTGTTAAAGATTTTGGGACCATAAACTAATTTTCTAACTTTAAATGTTTGTATATACTTCACAACTATAAATACCAA
91    CTATCCCCTACATGCATGCATGAACTCACCACTAGCTATACTATACGAAAGAAAGAATAAGAGGAAAGAAAAGAAAGAATAAGAGGAAAG
181   AAATGGATGAAGTATCCAAAGAAAAGCCAAAGGAGATGGTGATGAAATCTCCTCCAAGGCTCTTTAGGAGAAAAAAATGGACAAAGTTTG
1                                                 M  V  M  K  S  P  P  R  L  F  R  R  K  K  W  T  K  F
271   ATAGAAACGTTGCCTTTTACTTTCTCAGTTTGCATCTACTTTGTATTTTGGCACCCTTCTATTTTAGTTGGACCTGTTTGTTGCTCGCCT
19    D  R  N  V  A  F  Y  F  L  S  L  H  L  L  C  I  L  A  P  F  Y  F  S  W  T  C  L  L  L  A
361   TAGGCTTATCTTATTTAACAGGTATGGGAATAAGTGTCTCGTATCATCGAAATCTTGCACATCGAAGCTTCAAGCTTCCAAAATGGCTTG
49    L  G  L  S  Y  L  T  G  M  G  I  S  V  S  Y  H  R  N  L  A  H  R  S  F  K  L  P  K  W  L
451   AGTATTCTCTTGCTTATATTGCAACTCATGCAATCCAGgtttgatattttgaattctttgattttatatttagtttaagactattttgaa
79    E  Y  S  L  A  Y  I  A  T  H  A  I  Q
541   ttctttgattttatatttagtttaaaactatacatgatggataatacaaaatatttaaatttttttgtcacacattagtataaacttataa
631   atcaataagatgtgtaacatgatatatatgggagtaggtggttcattattgatttttacatatggtaaccctacaagtgaggatttatta
721   gtgttgccttagctactttggattcatttgtcatgccaaaatcatcattatttatggttatgccatattttgtgataatttgtatataca
811   ctacttaaaacaccaaaacattattaaagaatggtgtagtgtaattattgcctacatgtgatttagaagggtaaaactagtgacaaaacc
901   ctagtgcatatttttttatatctaccgtgttttgttaaaatttcgtttactttttttctttcttaattttagGGTGATCCAATTGATTGGG
92                                                                            G  D  P  I  D  W
991   TGAGTACACATAGATGTCACCATCGATTCGTTGATTCAGCACGAGATCCACATAGTCCTATTTATGGATTCTGGTTTAGTCAAATGACTT
98    V  S  T  H  R  C  H  H  R  F  V  D  S  A  R  D  P  H  S  P  I  Y  G  F  W  F  S  Q  M  T
1081  GGTTTTTTGATTCGTATAACTTGACGAGAAAAgtttgtccaaactattttaataattatcaaaaggtggagagaaacatcttcatgtttt
128   W  F  F  D  S  Y  N  L  T  R  K
1171  acatgaaaCATGGATATCCAGACAATGTGACAGATTTGGAGAAACAAAAGTTCTATAGGTTTATACATAAAACATATTGGCTTCATCCAC
139            H  G  Y  P  D  N  V  T  D  L  E  K  Q  K  F  Y  R  F  I  H  K  T  Y  W  L  H  P
1261  TTGCCCTTTCACTCTTGCTCTATGCCATTGGAGGACTACCTTTCATCATTTGGGGAATGgtataattattcccattctttttttagctttt
166   L  A  L  S  L  L  L  Y  A  I  G  G  L  P  F  I  I  W  G  M
1351  actcctacaatttgtaagttgtgattaaatttatttatgagtcctctaaaaattagtttacattcatcttttataaattctatttgtttg
1441  gatttttgacattttaacttaactttggtaaactagcaaatttaataatttttttttgaaggaaagactgtatgtttttgtattgacataa
1531  caaaggtagttgaattttatttttgactattttacttaaatgtttactcaaatattattgtttatcttttatgaactgtgacagaccaaa
1621  ataaactattatttaagttctattgtgatagacacatgtaatagtctatattttgatccgtcatacctaaattgtgctattttaatatat
1711  ttgtagtagttttgtctttttttattattatttattttacaattaatcattgattaacattatttatttggttaaactataaaatttaat
1801  tagtcttcaacgtttgaaacttgttcatgtctaattaagtctaagtctttataaaagatctagaagttatactgtgtatttagttaaatt
1891  caatcttatatttaagaagtttgtgaattttaggcttctttgataataattaatttgttatttttttcttctcttttttgtgtaaaactt
1981  atatatactttttttctccccatttttaatctatggtttcaaattgtttgcaaataataatatttttagtcaattccaatcaacaaccaa
2071  aatttgtaagatatatgtatttttttatttacttttcaaaacttgatcactgtttaatatgattagccataaatataagtagaaaaggt
2161  agttgaaatgtattttctaaatgatgtaagttcctttttcttcataaatagttttaccatctagttaattattctagatttaatgattata
2251  aaataataattataattttattaattattttcattgaaatattttactaaactaagtgttttgtgtttattaaacttgcgtatatagTGC
186                                                                                          C
2341  GTGAGGTTCGTATACAGCTTGCATGTGACATCTATGGTCAATTCAGTATGTCATATATGGGGAAATCAACCATGGAATACTGGTGATTTA
187    V  R  F  V  Y  S  L  H  V  T  S  M  V  N  S  V  C  H  I  W  G  N  Q  P  W  N  T  G  D  L
2431  TCCAAGAATAATTGgtatgacatttattttattttactttcctttgaatccctcaaaattcaattttactccctacattttcaaaatact
217    S  K  N  N  W
2521  tttcacaaccctaatttagataaaatcatatttgaataatatatgttctcttttcgttgttcttttcttcttttatagGATGGTAGCTTT
222                                                                                 M  V  A  L
2611  ATTAACCTTTGGAGAAGGATGGCACAATAACCACCATGCTTTTGAGTATTCAGCGAGACATGGTCATGAGTGGTGGCAAATTGATTATGG
226     L  T  F  G  E  G  W  H  N  N  H  H  A  F  E  Y  S  A  R  H  G  H  E  W  W  Q  I  D  Y  G
2701  TTGGTATCTTATTAGGTTCCTTCAGGTCATTGGATTAGCAACCAATGTGAAATTACCTTCTGAAAAGCAAAAATTGGCTGCTTTAAACAA
256     W  Y  L  I  R  F  L  Q  V  I  G  L  A  T  N  V  K  L  P  S  E  K  Q  K  L  A  A  L  N  K
2791  ATCAAAACCCATCTTTCTTAAGCTTATGATTGATTGA
286     S  K  P  I  F  L  K  L  M  I  D  *
```

>CsFAD5.14

```
1     ATTGTGGGACCATATCTAATTTTTTAACTTTAAATATTTGTACAATTCACGAGTATAAATACAAATGTGCCCTAGACTGAAGTCACAACT
91    ATACCAAAGAAAGAACAAGGAAAGAAATGGAAGGAGCATCCAAAGAAGACCCAAAGGAGATGGTGATGAGCTCTCCTTCAAGGCTCTTTA
1                                                                           M  S  S  P  S  R  L  F
181   AGAGAAAAAAGTGGACAAAGTTTGATAAAAACGTTGCCTTTTACTTTCTTCTTTTACATCTACTTAGTATTTTGGCACCATTATTCTATT
9     K  R  K  K  W  T  K  F  D  K  N  V  A  F  Y  F  L  L  L  H  L  L  S  I  L  A  P  L  F  Y
271   TTAGTTGGGGTTGTTTGTTGCTTTCCTTGGGATTAGCCAATCTAACCGGGATGGCAATTAGTGTTTCGTATCATAGAAATCTTGCACATC
39    F  S  W  G  C  L  L  L  S  L  G  L  A  N  L  T  G  M  A  I  S  V  S  Y  H  R  N  L  A  H
361   GAAGCTTCAAACTTCCAAAATGGCTCGAGTATTCTCTTGCTTATATTGCAGCTCATGCAATTCAGgtttgatactttgaatcccttatgc
69    R  S  F  K  L  P  K  W  L  E  Y  S  L  A  Y  I  A  A  H  A  I  Q
451   tctaactaaaaatggtctaaactacgaataaaacatgaattacacattttgattttatactaaatttgaggttggtactctatgacacat
541   tatttaattttacttcacctttttaattaatcaataatttaatatgatattagaataaatgtttcacagggttcttttgcattcaaaac
631   cattgcaatgtcattacctccttaatataattaatattgattctcaccttttggattttgtacacacatttcaatcccacaaaagttaga
721   gttaaatgattttgaatctaaaaacgaacagtgttgatcattacattatacagtgttgatcattacattatacatgaaagaatgattttt
811   atacacattacttagacatccaatcataaaaggattgagtagtgtatagtaattgcctacgtactattaaaaaagattaaattagagact
901   aatcctatcatatttttttatagcggtgttttgttaacatttggttggtttcttcaattttagGGTGATCCAATTGATTGGGTGAGCACAC
91                                                                        G  D  P  I  D  W  V  S  T
991   ACAGATGTCACCATCGATTTGTTGATTCAGAACGAGATCCACATAGTCCTATTTATGGATTTTGGTTCAGCCAAATGACGTGGTTTTTTG
100   H  R  C  H  H  R  F  V  D  S  E  R  D  P  H  S  P  I  Y  G  F  W  F  S  Q  M  T  W  F  F
1081  ATTCGTATAACTTGACGAGAAAAgtttgtccaaactattttaataattatcaaaaggtggagagaaacatcttcatgttttacatgaaaC
130   D  S  Y  N  L  T  R  K
1171  ATGGATATCCAGACAATGTGACAGATTTGGAGAAACAAAAGTTCTATAGGTTTATACATAAAACATATTGGCTTCATCCACTTGCTCTTT
138   H  G  Y  P  D  N  V  T  D  L  E  K  Q  K  F  Y  R  F  I  H  K  T  Y  W  L  H  P  L  A  L
1261  CACTCTTGCTCTATGCACTTGGAGGACTCCCTTTCATCATTTGGGGAGTGgtaaaatataattattattccctttccatttactcttta
168   S  L  L  L  Y  A  L  G  G  L  P  F  I  I  W  G  V
1351  attattaaggttataaactcctatttgtttgaatgttcaaaattttctttcaacttttctaaaagaaggaaattttaatctttttcatta
1441  ataaattaagttgttgatttttttacattgttatatttgatttgttcattacatgtttttaggcaatctacacaacagacaatttaagtg
1531  tttttttaaaaaataaatgaaagaaaaataatatgtatacatgtataatacttttttgtagattttcattatcgtaaataaaccctgaaat
1621  ctaaaagttttgtttttctttttacataaaggtaaaagatttatttgacaattaattattgatttatattatatataaagttaaaattat
1711  atataagtacgtaaacctaaaccctgaattctaaaatttatactaatctaattcttcatcttttaaaaatatttagtaggtttttggact
1801  atttgtatgtttaaacaagtcatactcaaatttcttttatcgagaagatttatgaatttaaaaataaataaattttatagtctcattcga
1891  taatgatttgttttaatttttttataatggttttcattttcttcaaataaacattaaaacctaattttcaagtaaaacactagatcgttg
1981  ataatcagtctaaagtattgtgtttgattatgattataattattagattatactagagagatggatgatctttccaccatatatttgttt
2071  aattatatatgtttttcattgaaatatatattttatttatgaagtgtattttattaaactattgacagTGCGTGAGGTTCGTACTGAGCT
185                                                                              C  V  R  F  V  L  S
2161  TGCATTTCACATCTATGATAAATTCAGTATGTCATATTTGGGGAAACCAACCATGGAACACTGGGGATTTATCTAAGAACAATTGgtaag
192   L  H  F  T  S  M  I  N  S  V  C  H  I  W  G  N  Q  P  W  N  T  G  D  L  S  K  N  N  W
2251  acatacacttttcttattatatctcttttctttttttattcttttcttttaccatttgctagtgaaaaatgggttttatattaattaatta
2341  atgttgatacatcaattttcatatttatatcatcaatccagttacataaaatttgaatattctatatcaaccctttaccatttgtttcga
2431  aataattttgctttttaaagtaggaagtaaatttttttaaaccttcaggacaattacaaagtttaaatagtttgttttaccactgtcaaac
2521  tattatagtatttagataatctttgacttgacaaagtttaaatagttcattgtttcatttttaattagGATTGTTGCTATATTTACCTTT
221                                                                               I  V  A  I  F  T  F
2611  GGAGAAGGATGGCACAATAACCACCATGCTTTTGAGTATTCAGCACGACATGGTCATGAATGGTGGCAAATTGATAATGGTTGGTATATT
228    G  E  G  W  H  N  N  H  H  A  F  E  Y  S  A  R  H  G  H  E  W  W  Q  I  D  N  G  W  Y  I
2701  ATCAAGTTCCTTCAAGTCATTGGATTAGCCACCAATGTTAAATTACCTTCTGAAAAGCAAAAAATGGCTGCTTTAAACAAATCAAAACCC
258    I  K  F  L  Q  V  I  G  L  A  T  N  V  K  L  P  S  E  K  Q  K  M  A  A  L  N  K  S  K  P
2791  ACCTTTCTTAGGCCTATGATTGATTGA
288    T  F  L  R  P  M  I  D  *
```

>CsFAD6

```
1      TTTTGTTTTTGCTTACAATTTCGTTGTGGGGGCAGAAAAAAAATGAGTGATAACAACGCCTACTTGAACACTAGTGGCGCAAGCTCGAAA
91     GGGCTAACCCCTTTCAGAGGATTCTATCCACATCTTAAAGAACGATTGGTGAAAGATTTATTCCCAATTTCCCATTTCAAATTGCAAACT
181    TCCATACTTAAGGGGATACCCATTAAGAAATTTCCCTCTTTCCCCCCTCTGTTGCCTCTTATTCGAATAGCCCAAGCAAGCAAGCTAAGG
271    GGGCCTGCTTAATTGGGATTTCAGTAATTCTTCACATAATGGCTTGCAGACTCGTAGATTCCATGGCTCTTTTCAATGTATGTTTTCTTC
1                                             M  A  C  R  L  V  D  S  M  A  L  F  N
361    TTTTTAATCTCTCCCTGCTATTGTACTTGTGTTCTTCTTTTCTCTTATTATTTTGGTTTGATTATCAGGGCCCCCATCATAATTTTGCTC
14                                                                           G  P  H  H  N  F  A
451    GAACCCAGAAAATTGCATCCCACTGTTCTCCAGgtatgttttagtatcttccttttgagaattatttgaagctgtttaagaatttgtaat
21     R  T  Q  K  I  A  S  H  C  S  P
541    tacttgataaactgaatcgtctagcggttgtcttattacagtgtgacacagttagagtatattcattgaagattcaatctcatttccttt
631    gtcactccggaagaaaccttgctgtttcagacatggtttcttttaataaaatattgagtaaaattggaaattagatagatttagagtcta
721    gctcgttctggaagtttagattttcaatcacattgataaattttcatatcctcattcaacttaagcatggattttgatatgccataaaat
811    gaattaattgactaggaaccaacatagctttgatttccattatcaatttagacagttcaaacatgcttattccgatagacagGTATATT
32                                                                                        G  I  L
901    GCAAGTGAAGGGGGAAATATTATTTCAGAAGAAAATCAAGCGACAAAACTATTTACTTCCTGTGAAGAGGGCTGCAATTCTACGAGCTAT
35       Q  V  K  G  E  I  L  F  Q  K  K  I  K  R  Q  N  Y  L  L  P  V  K  R  A  A  I  L  R  A  I
991    TGCTGTTCCAGCTGCGCCACCATCTTCTTCAGCAGATAGTGCTGAATATAGAAAACAATTATCAGAAAGCTATGGATTTGAGCAAATTGG
65       A  V  P  A  A  P  P  S  S  S  A  D  S  A  E  Y  R  K  Q  L  S  E  S  Y  G  F  E  Q  I  G
1081   AGAACCTCTTCCTGATAATGTTAGGTTAAAAGATGTTGTTGAGTCCCTTCCAAAAGAGgtactttattttgttgcaacatactggtgttt
95       E  P  L  P  D  N  V  R  L  K  D  V  V  E  S  L  P  K  E
1171   aacatttgtccttgaggcagtttcattttcttttcttctgtcagataaaattttgatgatgctatctagttcatgcatacgatcgtgttt
1261   cttttacttattatatcttatttttcattttttaaagGTGTTTGAGATCAATGATGTGAAAGCATGGAAATCTGTTTTAGTATCTGTA
114                                             V  F  E  I  N  D  V  K  A  W  K  S  V  L  V  S  V
1351   GCTTCCTACTCATTAGGCCTTGTCATGATTGCAAAAGCTCCATGGTATCTACTACCTTTGGCTTGGGCATGGACTGGAACAGCAATAACT
131      A  S  Y  S  L  G  L  V  M  I  A  K  A  P  W  Y  L  L  P  L  A  W  A  W  T  G  T  A  I  T
1441   GGGgttagtctcaatattctttctggttgcttccctttccctttctcttcttctcatccttatcagacttgcaactctagccactgaagt
161      G
1531   ttctttcctcctcaagTTCTTTGTTATAGGTCATGATTGTGCTCACAAATCATTTTCAAGGAACAAGTTAGTGGAAGACATTGTGGGTAC
162                     F  F  V  I  G  H  D  C  A  H  K  S  F  S  R  N  K  L  V  E  D  I  V  G  T
1621   TTTGGCTTTCATGCCTCTAATATACCCATATGAGCCTTGGCGGTTTAAGCATGATCAACATCATGCAAAGACAAACATgtattaataatc
187      L  A  F  M  P  L  I  Y  P  Y  E  P  W  R  F  K  H  D  Q  H  H  A  K  T  N  M
1711   tttcctctatctctctctctctttgaaatacttgtgtgagctctatcttcctgcagaagcattgttcagtaatatgtaaagaaatgtctt
1801   attgaggttggatagcatctagttaagcaatgcaaaagccttgataagtaatatgagcagaacaatacggatggatgttagtgtgttatt
1891   gtaagggacctaatgagagatatatgagggttattagtagaatattagtatggttattagaaggggccaattagtaaatagctagtaagc
1981   tggttagagtttttagttataaattcagggaatgggttgagagaaaggtgcgaagaattttgtgggatttccttgtgaggaatttgggaa
2071   agtctagccctctgggggttacgtttttttacttttctgtctaattagttcccttacacttataaatttataatcagtacctcgatagtac
2161   taggtttgttcacatgacgtcttatttacttgtcatggttagttctccttgtatcttgggatcaagttaacagaatttctccatatgttt
2251   tgcattttgcctagagtcaaatattgctgtgttaattaagtttgaaatactccacatatgaccaaataggaatccatattttataagttt
2341   ggtttgttacttgcatcttaacgatttatgcctttcatcgtctagttttagctaacctttcattttattcttatccacatattatagGC
2431   TGGAAGAAGATACAGCTTGGCATCCTGTGTGGAAAGAGGAATTTGATTCAGCTCCTCTTTTGCGTAAAGCAATCATATATGGTTATGGTC
213    L  E  E  D  T  A  W  H  P  V  W  K  E  E  F  D  S  A  P  L  L  R  K  A  I  I  Y  G  Y  G
2521   CATTTCGACCGTGGATGTCTATAGCACATTGgtaagtttaccttggatggttactttgctagaatagtttgactttctatgaaaatttat
243    P  F  R  P  W  M  S  I  A  H  W
2611   tgtacgtgatttgaccaagttgtctgtttcattaatatgcaccaatttgctcttatgcatgatagGTTGTTGTGGCACTTTGATTTGAAG
254                                                                        L  L  W  H  F  D  L  K
2701   AAGTTCAGACCAAATGAAGTAAAAAGAGTGAAGATAAGCTTAGCTTGTGTTTTTGCATTTATGTTTATTGGATGGCCATTGATCATCTAC
263     K  F  R  P  N  E  V  K  R  V  K  I  S  L  A  C  V  F  A  F  M  F  I  G  W  P  L  I  I  Y
2791   AAGACTGGGATTCTTGGATGGGTCAAGTTCTGGTTAATGCCATGGCTGGGATACCACTTTTGGgtaacgtcatttgcttgcctcttctta
293     K  T  G  I  L  G  W  V  K  F  W  L  M  P  W  L  G  Y  H  F  W
2881   ttacagtttcttctatagtcctatgatcaattcaattgatgatctgcgttggatagtgatttgtcatggttcttcatgatgtgacaatgg
2971   attaagaaactagttaacaatcagaattagaggatatatggcagggtagactttaatgaaaatgaaccatgtagcttagccggtggtatc
3061   caagtatatcttgatattgttgaaacatcaatcttaatgaagcttacatcttgcactaatattcttgaagcaaataaaagaatgtgaata
3151   ccatgctttcgagatcatgagacaatgatacgatgtttgtgccacttttcttcattcatagagggacgatatttattgttcttgttctgc
3241   ataactttgaaattgtcccagataaaatttgttggttttgcttttgtaaacttaacttctatttaaattaccttttgtttctttcttagA
3331   TGAGCACTTTCACCATGGTCCATCACACAGCTCCTCATATACCGTTTAAGTCATCAGAAGAGTGGAACGCAGCTCAAGCACAGCTCAATG
314    M  S  T  F  T  M  V  H  H  T  A  P  H  I  P  F  K  S  S  E  E  W  N  A  A  Q  A  Q  L  N
3421   GAACAGTTCACTGTGCTTATCCTCAATGgtactgaatcattcatttcagtgtctttttttccttttcttttcttttctcttttcttgctt
344    G  T  V  H  C  A  Y  P  Q  W
3511   aattcgactctaagccgataaaaaggcacatcggaaaaagattcaggcactgttcatgagattctctagtcttgaaattgctagataatt
```

```
3601  catgttaactaagatttattaccataagtcacattgacttagcttctaagtttatcctctcccatctacagGATCGAGATTCTCTGCCAT
354                                                                           I  E  I  L  C  H
3691  GACATCAATGTGCACATTCCCCACCATATTTCTCCAAGAATACCGAGTTACAACTTACGGGCAGCTCATAAGTCTCTTCAAGAAAACTGG
360    D  I  N  V  H  I  P  H  H  I  S  P  R  I  P  S  Y  N  L  R  A  A  H  K  S  L  Q  E  N  W
3781  GGAAAGgtaatggggttaaaccaaatgcatatgtacatagttcgatgttcagatcttgcaaaatctaatggttgccttgtttattgcata
390    G  K
3871  tattttcccccattgatgctaataatctagttcttttcttccactttgttacacattctgtagCATCTGAATGAGGCTTCATGGAATTGG
392                                                                  H  L  N  E  A  S  W  N  W
3961  CGATTAATGAAGACGATCATGACGATATGCCATGTATACAACAAAGAGGAAAATTATGTCGCATTTGACCGACTCAATAAAGAAGATTCT
21     R  L  M  K  T  I  M  T  I  C  H  V  Y  N  K  E  E  N  Y  V  A  F  D  R  L  N  K  E  D  S
4051  TATCCCATTACATTCCTCAAGAAAGTCATGCCTGATCTTGCTTGATTTAATCTTTACCTCAACCTTCGGGAACACAGTCCACAGACACTT
41     Y  P  I  T  F  L  K  K  V  M  P  D  L  A  *
4141  GCTGCTTTCTTTCTCTGTGCAATGTTTTCTCCACAGATTTTGAAGAACGAATGGAAGGAAAGCTTGGTTTAGTTGACTGCCATATATTGA
4231  GTTAAAGAATTTCTCTATTCCATTTTCCAGTCTCTCTCTCCTCAAATAGCAATATTATGTAACCTTTTCTTTTAAGGTAGTTGTGATCTA
4321  AAGAGGGAAAACAGACCTTTGGTGTTTTATGTTGCCAACTTCTTAAAAAGGTGGAATGGAATATGGAAACATTCTCTTTATCAAAGACAC
4411  CTACTGACAAAGATCAATACAACACAACCATTATCATTGCAAGCGAA
```

>CsFAD7

```
1       GGGGGGGCCAAGTTGGAGGACAATCAACCAGAAACACAAGTTGGGTTTTTATGGTTGTAAGTGAATGAAGAGATTTCTGGCTGTGTTATC
91      TCATATCTGCTTCTGATTTCCCTTTTTATTTTAGGGTTTTGTTTGTTACCCATCTTTTCTTTTTCTTTATCTTAAGCTGTTTGAGGCTTA
181     AAAACTTGAGGCTACTTGTTTTTTCTCATCTGGGTCTCTAATGGCGAGGTGGGTCTTATCTGAATGTGGTGTTAAACCACTCCCTAGGAT
1                                               M  A  R  W  V  L  S  E  C  G  V  K  P  L  P  R  I
271     CTATCCTCTGCCATCTAGGAATGGGTTTGCTTCTAAAACTTTTATGGTTTCTGAGATTAGGCAGCCATTGTTGGCCTCTAAAGCTCATGT
18       Y  P  L  P  S  R  N  G  F  A  S  K  T  F  M  V  S  E  I  R  Q  P  L  L  A  S  K  A  H  V
361     TCTTCGCTCTTCTTTGGGCCTTTCAACTAAAGGGAAGAGTTGGGCATTGAATGTGAGTACTCCTGTGGCAGTTGCTTCTGTTGATGAGGA
48       L  R  S  S  L  G  L  S  T  K  G  K  S  W  A  L  N  V  S  T  P  V  A  V  A  S  V  D  E  D
451     TAATGAGAGGGAGAGAGTTAATGAGGTTAATGGGGTTGAGGATGAGGGTAGTTTCGACCCGGCTGCTCCTCCGCCGTTTCGGTTGGCTGA
78       N  E  R  E  R  V  N  E  V  N  G  V  E  D  E  G  S  F  D  P  A  A  P  P  P  F  R  L  A  D
541     TATTCGTGCTGCCATTCCAAAGCATTGTTGGGTTAGGGATCCTTGGAGATCAATGAGTTATGTTGTGAGGGATGTGGTGGTGGTATTTGG
108      I  R  A  A  I  P  K  H  C  W  V  R  D  P  W  R  S  M  S  Y  V  V  R  D  V  V  V  V  F  G
631     CTTGGCTGCTGTTGCTGCTTACTTCAACAATTGGGTTGTTTGGCCTCTTTACTGGTTTGCTCAAGGAACAATGTTTTGGGCTCTTTTTGT
138      L  A  A  V  A  A  Y  F  N  N  W  V  V  W  P  L  Y  W  F  A  Q  G  T  M  F  W  A  L  F  V
721     TCTTGGTCATGATTGgtaatttccactcttttggaaccattgaatatctttacctgctttttgtcttggaattcccttgcctgatttgat
168      L  G  H  D  C
811     agtgtgttttcttaatggttagtttgattttgtttttgaatgtgcagTGGCCATGGCAGTTTCTCTAATGACCCAAATTTGAATAGTGTT
173                                                     G  H  G  S  F  S  N  D  P  N  L  N  S  V
901     GTTGGCCATATCCTTCATTCCTCAATCCTAGTTCCATATCATGGATGgttagttcttgattatctttctgaatcccgtttggtcaaatta
187      V  G  H  I  L  H  S  S  I  L  V  P  Y  H  G  W
991     ttctttttgtagctttaactttctatgacaacataaactatagtttcatatcatgttgttcttgaacgcagGAGAATTAGCCACAGAAC
203                                                                              R  I  S  H  R  T
1081    TCATCACCAGAACCATGGTCATGTCGAAAATGATGAATCGTGGCATCCTgtaaattccttgtacttctttcccataaaacaaagaaatga
209      H  H  Q  N  H  G  H  V  E  N  D  E  S  W  H  P
1171    tcatcttgttgaatctaattcaatagcaatatgttcatatctgctttgcagTTGTCTGAGAAAATTTATAGGAGTTTGGATAAAGCAACA
225                                                         L  S  E  K  I  Y  R  S  L  D  K  A  T
1261    AGAACACTAAGGTTCGCTCTGCCGTTCCCCATGCTTGCATATCCTGTCTACTTGgttagtgaactctgtttcttcaacatccttctttgt
238      R  T  L  R  F  A  L  P  F  P  M  L  A  Y  P  V  Y  L
1351    gaacttcaatgttatgccaattctttgatggattctaatgaaaaatgttggtgatattgcagTGGAGTAGAAGTCCTGGAAAGAAGGGCT
256                                                                    W  S  R  S  P  G  K  K  G
1441    CTCATTTTGACCCTAACAGTGATTTGTTTGTACCGAGTGAGCGGAATGATATCATTACATCCACTGCTTGTTGGACTGCAATGCTTGCTC
265      S  H  F  D  P  N  S  D  L  F  V  P  S  E  R  N  D  I  I  T  S  T  A  C  W  T  A  M  L  A
1531    TGCTTGGATGTCTATCCGTAGTGATGGGTCCCCTCCAAATCATCAAACTCTACGGTATTCCTTACATGgtgagtttggttttgatcatga
295      L  L  G  C  L  S  V  V  M  G  P  L  Q  I  I  K  L  Y  G  I  P  Y  M
1621    attcacgatgaatatttcatgttttaaagttcaactactacgctcgattgacttctttttcaaagtgcactgctctgaatgtattcctt
1711    atgtagATGTTTGTCATGTGGCTGGATGCCGTCACTTACTTGCATCATCATGGTCACGATGATAAACTTCCTTGGTATCGTGGAGAGgta
318            M  F  V  M  W  L  D  A  V  T  Y  L  H  H  H  G  H  D  D  K  L  P  W  Y  R  G  E
1801    taccgttcaaatctttttgagttttggtgtggaatttccaagaacatcacaccataaaatgatctgttttacaccattttattaacttct
1891    ctgcttctgcacttgatgatcaaacagGAATGGAGTTACCTAAGAGGAGGATTAACGACCCTTGATCGTGATTACGGATGGATCAACAAC
345                                 E  W  S  Y  L  R  G  G  L  T  T  L  D  R  D  Y  G  W  I  N  N
1981    ATCCATCATGACATTGGAACTCACGTCGTTCACCATCTCTTCCCTCAAATCCCACACTATCACTTAATAGAAGCAgtgagtattcttctc
366      I  H  H  D  I  G  T  H  V  V  H  H  L  F  P  Q  I  P  H  Y  H  L  I  E  A
2071    agccctgcatctgaaattcaaacttaattttcttctatatattcgaaatcctcatcctaacaagtgcaaacttctcgtgttagACCGAGG
391                                                                                         T  E
2161    CAACGAAGCCGATATTCGGGAAGTATTATAGGGAACCAGAGAAATCTTGGCCTCTCCCATTTCACTTATTAGGAGTTCTTGTAAGAAGCC
393     A  T  K  P  I  F  G  K  Y  Y  R  E  P  E  K  S  W  P  L  P  F  H  L  L  G  V  L  V  R  S
2251    TAAGAAAGGATCACTTCGTGAGCGATTCTGGAGATATCGTATATTATCAAACAGACCCCGACCTGTCTTGATCAAAGCAACACAATTAAG
423     L  R  K  D  H  F  V  S  D  S  G  D  I  V  Y  Y  Q  T  D  P  D  L  S  *
2341    TTATGTTCATTGTCTTTCAACTATTACACATCCAAATTCTTTGCGAATAGCTCGCGAGCCACTCGAGTTTAAAATATGATCAGCTGCCAA
2431    AGAAGCCGTTGTTCACAATGATAGTATTCATGCTTTTAGGGTCTTTAGAGTTGCATATTATTATAGCCACATGAAAGAAAAAGTTGGCAT
2521    TGTTTTTGCTTGAAGTACGTTTAATAAACTACTAGTGAACCACAATGCTATTAATTTATATGGGATGAGTTGAATTTTTTTTTATTGTT
2611    CTTAAAT
```

S1 Fig. Nucleic acid sequences and the deduced amino acid sequences of 23 cucumber *FAD* genes.
