# Supplementary material for: Characterization of the Fatty Acid Desaturase Genes in Cucumber: Structure, Phylogeny, and Expression Patterns
Source: PLoS One. 2016 Mar 3;11(3):e0149917. doi: 10.1371/journal.pone.0149917 (PMC4777478; doi:10.1371/journal.pone.0149917)
Supplement: S2 Fig — The alignment was performed using Clustal X, followed by shading with Boxshade 3.21. The gaps are indicated as dashes. The conserved His-boxes are indicated with blue boxes, and the ER-retention signal is marked with red box. (DOCX) [file pone.0149917.s004.docx]

**Supporting Information**


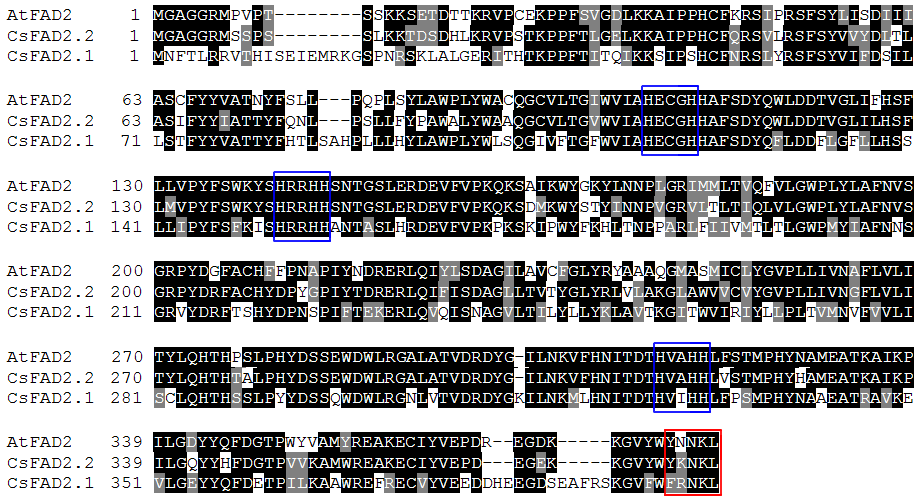


**S2 Fig. Sequence alignment of AtFAD2 and CsFAD2 proteins.**

The alignment was performed using Clustal X, followed by shading with Boxshade 3.21. The gaps are indicated as dashes. The conserved His-boxes are indicated with blue boxes, and the ER-retention signal is marked with red box.
